# Oligodendrocyte dysfunction in human age-related cognitive decline

**DOI:** 10.1038/s41591-026-04608-y

**Published:** 2026-08-25

**Authors:** Georgina A. Craig, Emeric Merour, Luise A. Seeker, Annette J. Haughian, Keon Arbabi, Jamie Rose, Alana Hoffmann, Jessica Thapar, Andrea Corsinotti, Stephen Mitchell, Janie Corley, Susan D. Shenkin, Colin Smith, Tara L. Spires-Jones, Lu O. Sun, Simon R. Cox, Anna Williams, Shreejoy J. Tripathy, Brian Popko, Véronique E. Miron

**Affiliations:** 1 Keenan Research Centre for Biomedical Science, St. Michael’s Hospital, Unity Health Toronto, Toronto, Ontario Canada; 2 Department of Immunology, The University of Toronto, Toronto, Ontario Canada; 3 UK Dementia Research Institute at The University of Edinburgh, Edinburgh, UK; 4 Department of Neurology, Northwestern University, Feinberg School of Medicine, Chicago, IL USA; 5 Department of Bioengineering, Stanford University, Stanford, CA USA; 6 Centre for Addiction and Mental Health, Toronto, Ontario Canada; 7 Institute for Neuroscience and Cardiovascular Research, The University of Edinburgh, Edinburgh, UK; 8 Wellcome Trust Centre for Cell Biology at King’s Buildings, The University of Edinburgh, Edinburgh, UK; 9 Lothian Birth Cohorts, Department of Psychology, The University of Edinburgh, Edinburgh, UK; 10 Ageing and Health, Usher Institute, The University of Edinburgh, Edinburgh, UK; 11 Department of Molecular Biology, The University of Texas Southwestern Medical Center, Dallas, TX USA; 12 Centre for Regenerative Medicine, Institute of Regeneration and Repair, The University of Edinburgh, Edinburgh, UK

**Keywords:** Oligodendrocyte, Cellular neuroscience

## Abstract

Cognitive decline in aging impacts quality of life, for which therapeutic interventions are lacking. Why some individuals experience more severe cognitive decline with aging is unclear. Here we uncover a role for the brain’s myelin-forming cells—oligodendrocytes—in explaining this variance. By investigating neuropathological and transcriptomic changes in human white matter associated with individual rates of cognitive decline in aging, we find that worse cognitive trajectories unexpectedly associate with smaller myelinated axon size, thicker myelin and more oligodendrocytes with downregulation of NRF2. Aged oligodendrocyte-specific NRF2-knockout mice showed attenuated cognitive improvement over time and mirrored the white matter pathology of human cognitive decline in aging. These findings place the oligodendrocyte as a contributor to cognitive decline and highlight the NRF2 pathway as a therapeutic target to preserve cognition in human aging.

## Abstract (web summary)

This study identifies oligodendrocytes as active contributors to cognitive decline in aging, by leveraging a unique human brain bank with more than 70 years of associated cognitive data combined with experimental modeling of oligodendrocyte dysfunction.

## Main

Cognitive decline, defined by impaired memory, reasoning and processing speed over time, is a major socioeconomic burden, rising in prevalence due to the aging population. The underlying mechanisms driving cognitive decline in aging remain unclear. Learning in humans in youth and adulthood is associated with increased central nervous system (CNS) myelin^1^, the insulating membrane produced by oligodendrocytes that surrounds neuronal axons to facilitate rapid electrical impulse conduction and provide metabolic support. Furthermore, it is now well established that, in mice, learning requires an increase in oligodendrocytes through de novo generation^2–6^. Cognitive impairment in aged mice and non-human primates is associated with reduced oligodendrocytes and myelin^6,7^; however, the white matter pathologies driving human cognitive decline remain unclear. Indeed, although magnetic resonance imaging studies associate white matter abnormalities with cognitive decline in aged humans^8–12^, the underpinning oligodendrocyte and myelin changes are unknown. In the present study, we identified the myelin and oligodendrocyte pathology specific to human age-related cognitive decline and unexpectedly discovered that an increase in dysfunctional oligodendrocytes has a functional contribution.

## Results

### Severity of cognitive decline in aging humans is associated with a shift in myelinated axon profiles and thicker myelin

**Fig. 1:**
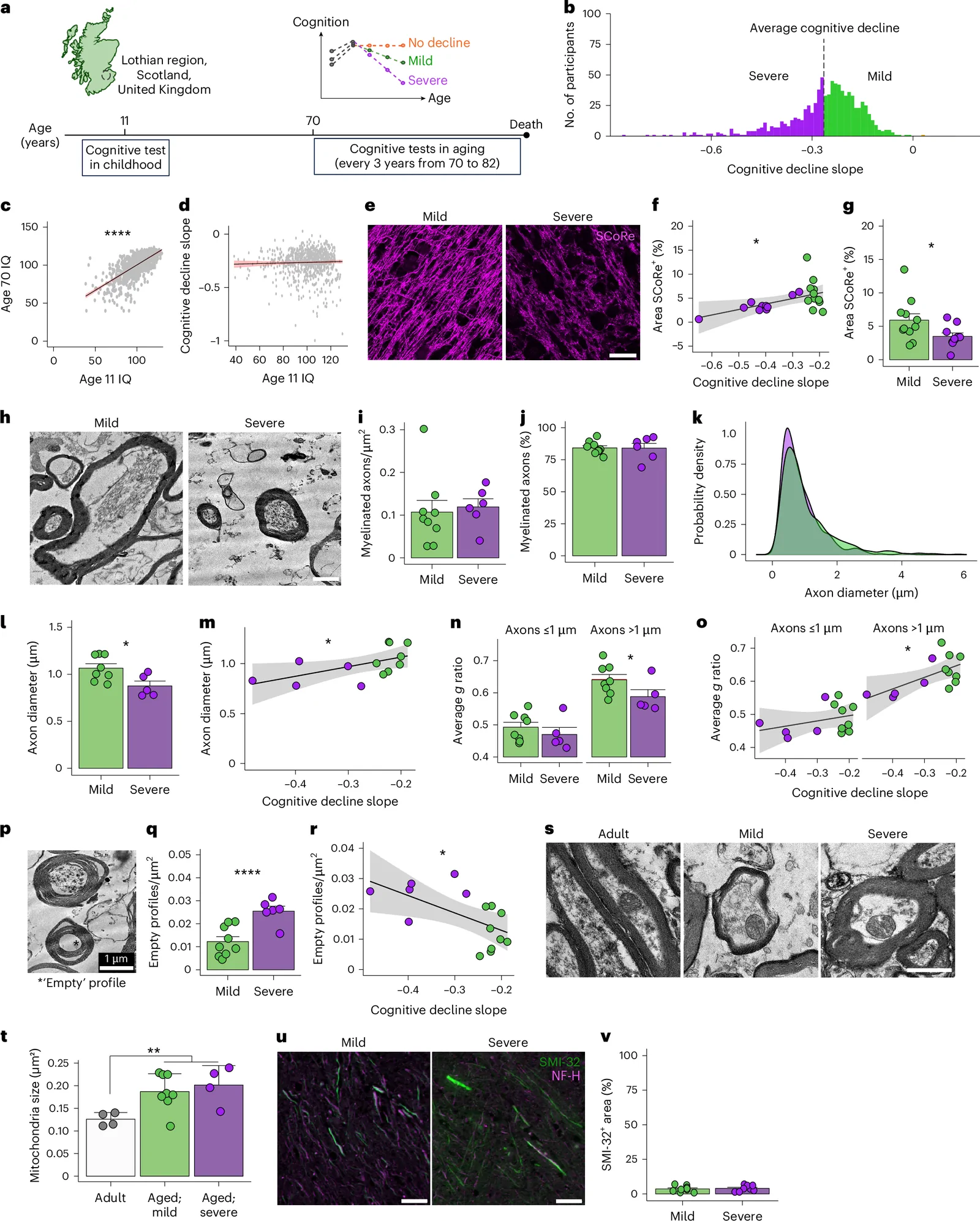
Severity of cognitive decline in human aging is associated with a shift in myelinated axon profiles and thicker myelin. **a**, Schematic of cognitive testing regime over life. Cohort members resided in the Lothian region of Scotland, United Kingdom, and were tested for cognition at age 11, then again at age 70 and every 3 years until age 82. Plotting individual cognition trajectories with aging stratifies individuals into those with no age-related decline (orange), mild decline (green) or severe decline (purple) beyond age 70. **b**, Number of individuals within the LBC1936 with no, mild or severe cognitive decline (*n* = 866 individuals). **c**, Age 11 IQ scores versus age 70 IQ scores. Two-sided Pearson’s correlation (with red shaded area representing the 95% confidence interval), *R* = 0.69, *P* = 2.2 × 10^−16^, *n* = 1,017 individuals. **d**, Slope of cognitive decline beyond age 70 versus age 11 IQ scores. Two-sided Pearson’s correlation (with red shaded area representing the 95% confidence interval), *R* = 0.036, *P* = 0.31, *n* = 816 individuals. **e**, Representative zoom-in images of SCoRe microscopy of compact myelin from individuals with mild versus severe cognitive decline. Scale bar, 20 µm. The experiment was carried out with samples from 21 individuals. **f**, Relationship between the percentage area positive for SCoRe signal in corpus callosum white matter from each individual and their cognitive decline slope. Two-sided Pearson’s correlation, *R* = 0.5, *P* = 0.02, *n* = 21 individuals. Shaded area represents the 95% confidence interval. **g**, Average percentage area positive for SCoRe signal ± s.e.m. from individuals with mild or severe cognitive decline. Two-sided unpaired Student’s *t*-test, *P* = 0.041, *n* = 21 individuals. **h**, Representative electron micrographs of corpus callosum from individuals with mild or severe decline. Scale bar, 1 µm. The experiment was carried out with samples from 15 individuals. **i**, Average density of myelinated axons (per µm^2^) in the corpus callosum ± s.e.m. in individuals with mild or severe decline. Two-sided unpaired Student’s *t*-test, *P* = 0.73, *n* = 15 individuals. **j**, Average percentage of myelinated axons in the corpus callosum ± s.e.m. of individuals with mild or severe decline. Two-sided unpaired Student’s *t*-test, *P* = 0.96, *n* = 15 individuals. **k**, Probability density functions describing myelinated axon diameter distributions for individuals with mild cognitive decline (green) versus severe cognitive decline (purple) (*n* = 13 individuals). **l**, Average axon diameter in the corpus callosum ± s.e.m. in individuals with mild versus severe cognitive decline. Two-sided unpaired Student’s *t*-test, *P* = 0.024, *n* = 13 individuals. **m**, Relationship between an individual’s average axon diameter and their slope of cognitive decline. Two-sided Pearson’s correlation, *R* = 0.57, *P* = 0.042, *n* = 13 individuals. Shaded area represents the 95% confidence interval. **n**, Average *g* ratio of myelinated small (≤1 µm in diameter) versus large (>1 µm in diameter) corpus callosum axons ± s.e.m. in individuals with mild versus severe cognitive decline. Two-sided Wilcoxon rank-sum test for small-diameter axons (*P* = 0.44) and large-diameter axons (*P* = 0.045) (*n* = 13 individuals). **o**, Two-sided Pearson’s correlation between average *g* ratio in myelinated axons of small (≤1 µm; *R* = 0.33, *P* = 0.28) or large (>1 µm; *R* = 0.62, *P* = 0.025) diameter and slope of cognitive decline (*n* = 13 individuals). Shaded area represents the 95% confidence interval. **p**, Representative image of an ‘empty’ myelinated axon profile (asterisk), classified as degenerating. Scale bar, 1 µm. The experiment was carried out with samples from 15 individuals. **q**, Average density of degenerating axonal profiles (per µm^2^) in corpus callosum ± s.e.m. in individuals with mild versus severe cognitive decline. Two-sided unpaired Student’s *t*-test, *P* = 0.00094, *n* = 15 individuals. **r**, Average density of degenerating axonal profiles (per µm^2^) per individual plotted against the slope of cognitive decline. Two-sided Pearson’s correlation, *R* = −0.60, *P* = 0.019, *n* = 15 individuals. Shaded area represents the 95% confidence interval. **s**, Representative images of mitochondria in myelinated axons from adult white matter versus mild or severely declined aged white matter. Scale bar, 1 µm. The experiment was carried out with samples from 16 individuals. **t**, Axonal mitochondrial size (µm^2^) ± s.e.m. in adult white matter versus mild or severely declined aged white matter. Two-way ANOVA for age (*P* = 0.0079) and cognitive status (*P* = 0.53) (*n* = 16 individuals). **u**, Representative zoom-in images of SMI-32 and NF-H staining in corpus callosum from individuals with mild versus severe cognitive decline. Scale bar, 50 µm. The experiment was carried out with samples from 17 individuals. **v**, Percentage area positive for SMI-32 signal ± s.e.m. in individuals with mild versus severe cognitive decline. Two-sided unpaired Student’s *t*-test, *P* = 0.71, *n* = 17 individuals. Schematic in **a** created in BioRender; Miron, V. https://biorender.com/ydlh70a (2026). Area SCoRe^+^ (%), percentage area positive for SCoRe signal; SMI-32^+^ area (%), percentage area positive for SMI-32 signal.

**Extended Data Fig. 1:**
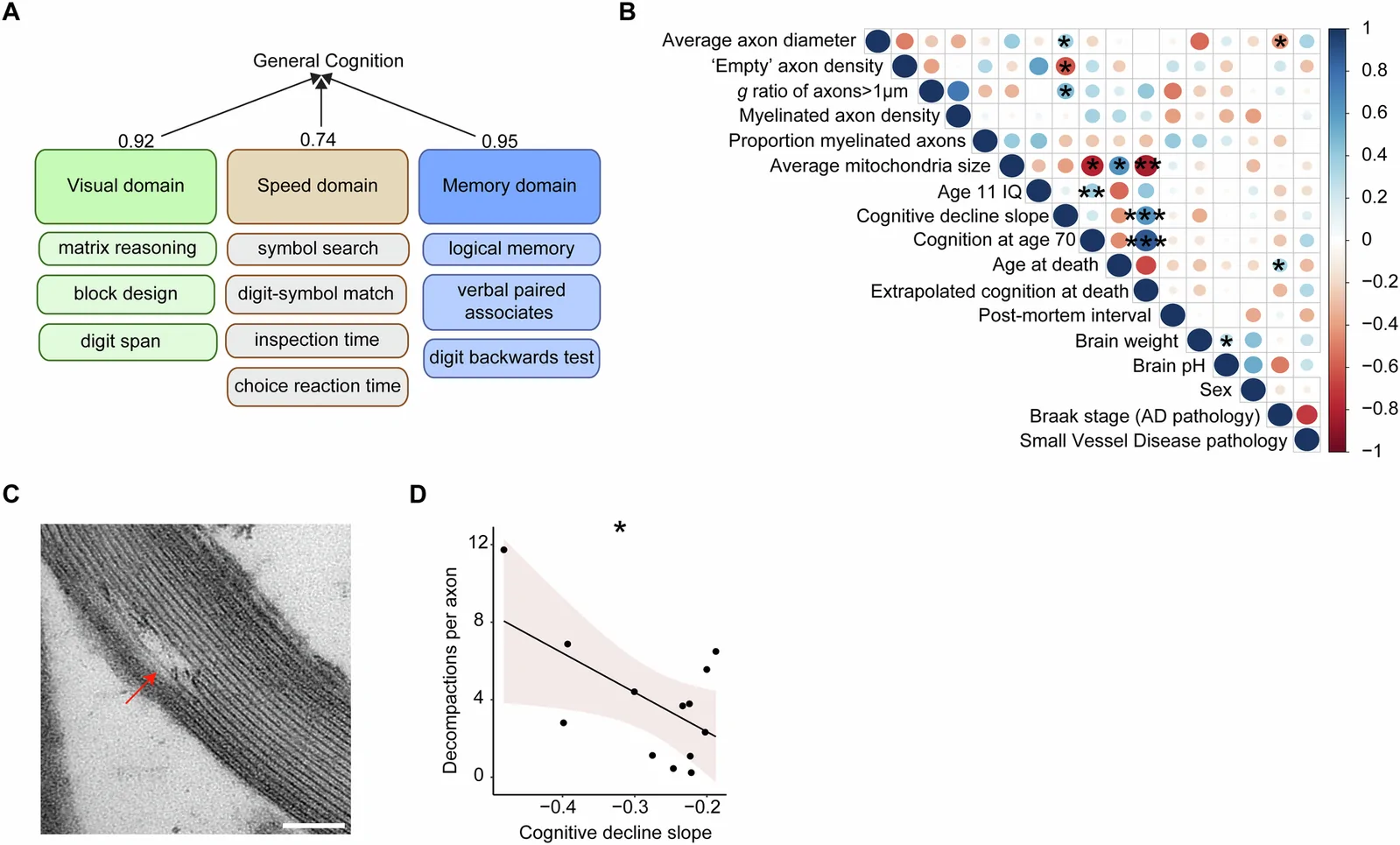
Cognitive decline severity associates with neuropathology but not clinical or technical variables. **A**. Structural equation modelling of generalized cognition beyond age 70, using 10 standardized tests across 3 cognitive domains. n=866 individuals. **B**. Correlation Matrix (Pearson’s) of cognitive and clinical variables against neuropathological correlates of myelin and axon health. Red indicates negative associations, blue indicates positive associations, and size of circle indicates strength of association. All significant correlations are indicated with asterisks. n=13-1,017 individuals. **C**. Representative image of decompacted fibres (arrow) in the myelin sheath. Scale bar, 100 nm. The experiment was carried out on samples from 13 individuals. **D**. Quantification of the average number of decompacted fibre bundles in large-diameter (>1 µm) axons with cognitive decline slope, with shaded area representing the 95% confidence interval. Correlation tested for via Pearson’s correlation, R=-0.58, *P*=0.038, n=13 individuals.

We first aimed to investigate white matter health in association with cognitive decline in aging. We examined samples from the Lothian Birth Cohort 1936 (LBC1936), the longest-running study of human cognition. Individuals from this cohort were born in 1936 and living in the Lothian regions of Scotland at recruitment, tested for IQ at age 11, then followed up at age 70 and every 3 years thereafter^13^; this allowed us to associate tissue pathology with individual lifelong trajectories of cognitive decline (Fig. 1a). To comprehensively assess cognitive decline with aging, cognition from 70 years to 82 years was measured with a series of standardized tests across visuospatial, processing speed and memory domains (Weschler Adult Intelligence Scale III/Memory Scale III) (Extended Data Fig. 1a). Cognitive level and decline were modeled using latent growth curve modeling (factor of curves) on cognitive data^14–16^. Individual rates of cognitive decline were defined as the slope change over time (annual change in cognitive level) for all individuals who had follow-up testing beyond age 70 (*n* = 866/1,017). Almost all individuals with follow-up testing beyond age 70 exhibited cognitive decline (*n* = 865/866). The average rate of cognitive decline from age 70 was −0.26; individuals with steeper than average rates of change were classed as ‘severe’, whereas those with less negative than average rates were classed as ‘mild’ (Fig. 1b). Whereas age 11 IQ was predictive of age 70 IQ (Fig. 1c), cognitive decline trajectories beyond age 70 did not relate to age 11 IQ (Fig. 1d), as previously shown^15,16^.

We investigated the corpus callosum given that it is a large white matter region involved in cognitive processing^17–19^ and is vulnerable to age-related pathology^20,21^ (cohort characteristics in Supplementary Table 1). To assess gross changes in myelin, we used spectral confocal reflectance (SCoRe) microscopy to specifically detect compacted myelin^22^, which showed a significant decrease in individuals with severe cognitive decline (Fig. 1e–g). To assess myelin integrity and myelinated axonal health on an ultra-structural level, corpus callosum samples from a subset of LBC1936 individuals were processed for transmission electron microscopy (Fig. 1h). No differences were observed in the density or percentage of myelinated axons (Fig. 1i,j). However, individuals with more severe cognitive decline had a shift in myelinated axon profiles toward smaller axon diameters, as indicated by probability plots of the frequency distribution and average myelinated axon size (Fig. 1k−m). Myelin thickness was surprisingly increased with worse cognitive decline, measured by a reduced *g* ratio (ratio of axon diameter: axon + myelin diameter) but only for large-diameter axons (>1 µm) (Fig. 1n,o). Consistent with decreased SCoRe signal, cases with severe cognitive decline had increased myelin decompactions in large-diameter axons (Extended Data Fig. 1c,d); however, these did not artificially impact myelin thickness measurements as there was no significant relationship to *g* ratio (*R* = −0.36, *P* = 0.22).

We also noted several instances where myelinated profiles were present but had no apparent axon (labeled ‘empty’; Fig. 1p), suggestive of the early stages of Wallerian degeneration^23^, which increased in frequency with worse cognitive decline (Fig. 1q,r). The degenerating and smaller axon profiles alongside thicker myelin could be indicative of a primary axonopathy, such as axon shrinkage. However, we did not observe any myelinated axons with established features known to precede axonopathy, such as axonal spheroids or condensed (dark) axoplasm^24,25^. In addition, average mitochondrial size, known to increase with axonal stress especially in large-diameter myelinated axons^26,27^, was not correlated with severity of cognitive decline yet was increased generally with aging, as expected^28^ (Fig. 1s,t). Furthermore, no difference was observed in the percentage of area positive for the axon pathology marker SMI-32 (ref. ^29^) between cases with mild and severe cognitive decline (Fig. 1u,v).

We acknowledge that many clinical parameters and technical artifacts from tissue processing are often confounding factors in human tissue analyses. Nonetheless, neither axon diameter, myelin thickness nor the density of degenerating myelinated axon profiles was associated with age at death, sex, age 11 IQ, technical variables (such as postmortem interval, brain weight, brain pH) or neuropathological indices of small vessel disease (Extended Data Fig. 1b). Moreover, we did not see a significant relationship between myelinated axon changes and absolute cognition (either at age 70 or extrapolated at death; Extended Data Fig. 1b). Therefore, it is worsened cognitive decline over time, rather than absolute cognition, that is associated with myelinated axon pathology. Interestingly, there was a significant negative association between Braak stage and axon diameter (Extended Data Fig. 1b), suggesting that smaller axons may be a shared pathological feature between age-associated cognitive decline and Alzheimer’s disease pathology. Together, these findings uncover a selective vulnerability of large-diameter myelinated axons to pathology in human aging, in association with worse cognitive decline.

### Oligodendrocytes deficient in NRF2 are increased in severe cognitive decline in humans

**Fig. 2:**
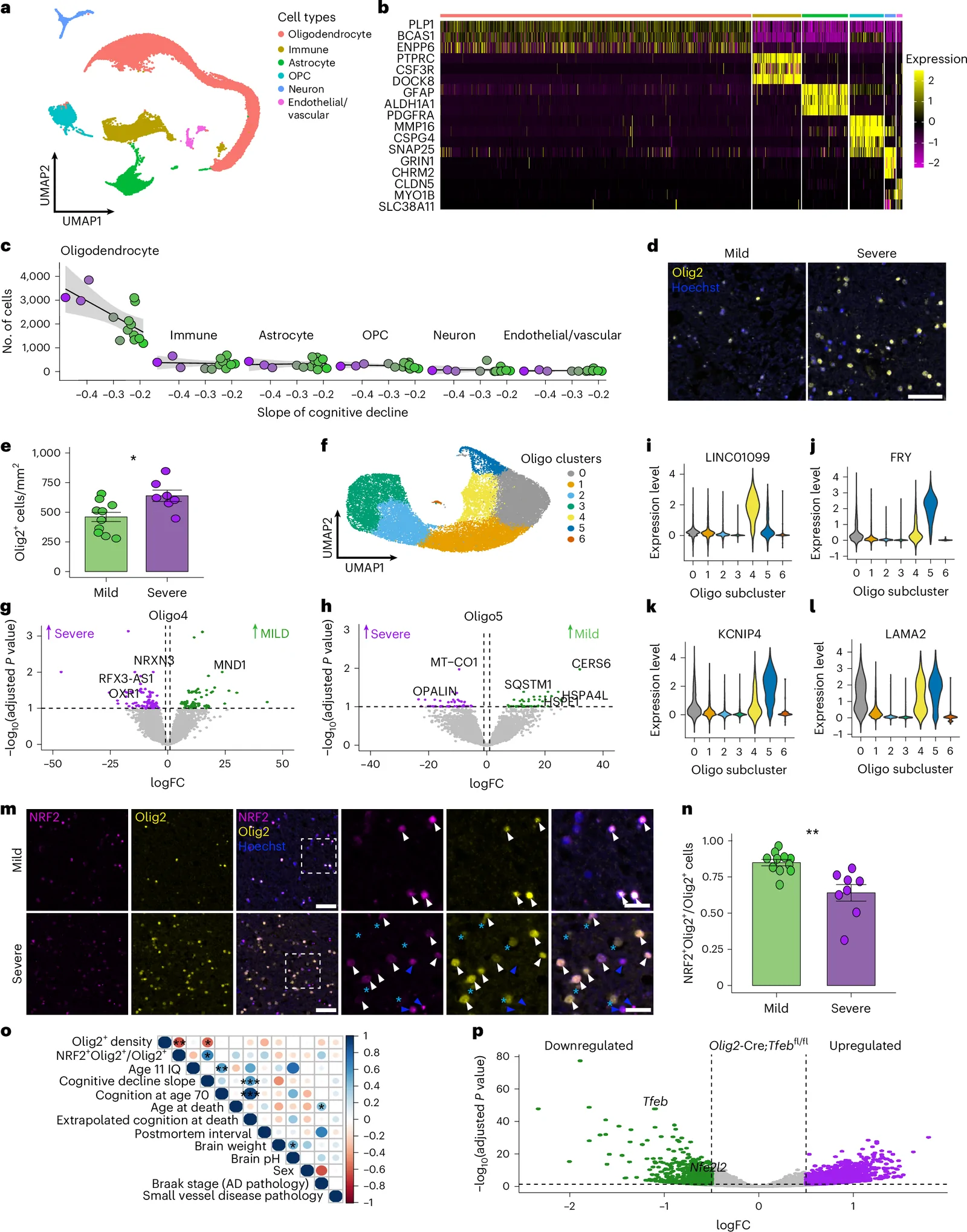
Oligodendrocytes with reduced NRF2 accumulate in severe cognitive decline in humans. **a**, UMAP of nuclei sequenced from LBC corpus callosum, colored by annotated cell type. **b**, Heatmap showing expression of canonical genes associated with annotated cell types. **c**, Nuclei per cell type cluster versus slope of cognitive decline. Lines represent two-sided Pearson’s correlations with 95% confidence intervals shown in gray. For all analyses, *n* = 14 individuals. **d**, Representative zoom-in images of Olig2 (yellow) and Hoechst (blue) in mild versus severe cognitive decline. Scale bar, 50 µm. The experiment was carried out with samples from 18 individuals. **e**, Density of Olig2^+^ oligodendrocytes per mm^2^ ± s.e.m. in mild versus severe cognitive decline cases. Two-sided unpaired Student’s *t*-test, *P* = 0.012, *n* = 11 mild cases, *n* = 7 severe cases. **f**, UMAP of oligodendrocyte subclusters. **g**, Volcano plot of DEGs (two-sided moderated *t*-tests with Benjamini−Hochberg multiple comparisons adjustment) enriched in Oligo4 in severe cognitive decline (purple) or mild cognitive decline (green). Dotted lines represent the threshold cutoffs of an adjusted *P* < 0.1 and an absolute log_2_ fold change greater than 1. **h**, Volcano plot of DEGs (two-sided moderated *t*-tests with Benjamini−Hochberg multiple comparisons adjustment) enriched in Oligo5 in severe cognitive decline (purple) or mild cognitive decline (green). Dotted lines represent the threshold cutoffs of an adjusted *P* < 0.1 and an absolute log_2_ fold change greater than 1. **i**, Violin plot of *LINC01099* expression across distinct oligodendrocyte subclusters. **j**, Violin plot of *FRY* expression across distinct oligodendrocyte subclusters. **k**, Violin plot of *KCNIP4* expression across distinct oligodendrocyte subclusters. **l**, Violin plot of *LAMA2* expression across distinct oligodendrocyte subclusters. **m**, Representative zoom-in images of protein expression of NRF2 (purple) and Olig2 (yellow), with NRF2^+^Olig2^+^ cells (white arrows), NRF2^−^Olig2^+^ cells (asterisks) and NRF2^+^Olig2^−^ cells (blue arrows) in mild (top panel) versus severe (bottom panel) cognitive decline. Scale bars, 50 µm (left) and 20 µm (right). The experiment was carried out on samples from 18 individuals. **n**, Proportion of NRF2^+^ oligodendrocytes ± s.e.m. in mild versus severe cognitive decline. Two-sided unpaired Student’s *t*-test, *P* = 0.0076, *n* = 11 mild cases, *n* = 8 severe cases. **o**, Correlation matrix (Pearson’s, two-sided) of cognitive and clinical variables against oligodendrocyte and NRF2^+^ oligodendrocyte densities. Red indicates negative associations, blue indicates positive associations and size of circle indicates strength of association. All significant correlations are indicated with asterisks. **p**, Volcano plot of increased (purple) or decreased (green) genes (two-sided moderated *t*-tests with Benjamini−Hochberg multiple comparisons adjustment) in *Olig*2-Cre;*Tfeb*^fl/fl^ compared to floxed-only littermates. Dotted lines represent the threshold cutoffs of an adjusted *P* < 0.05 and an absolute log_2_ fold change greater than 0.5. FC, fold change; OPC, oligodendrocyte progenitor cell.

**Extended Data Fig. 2:**
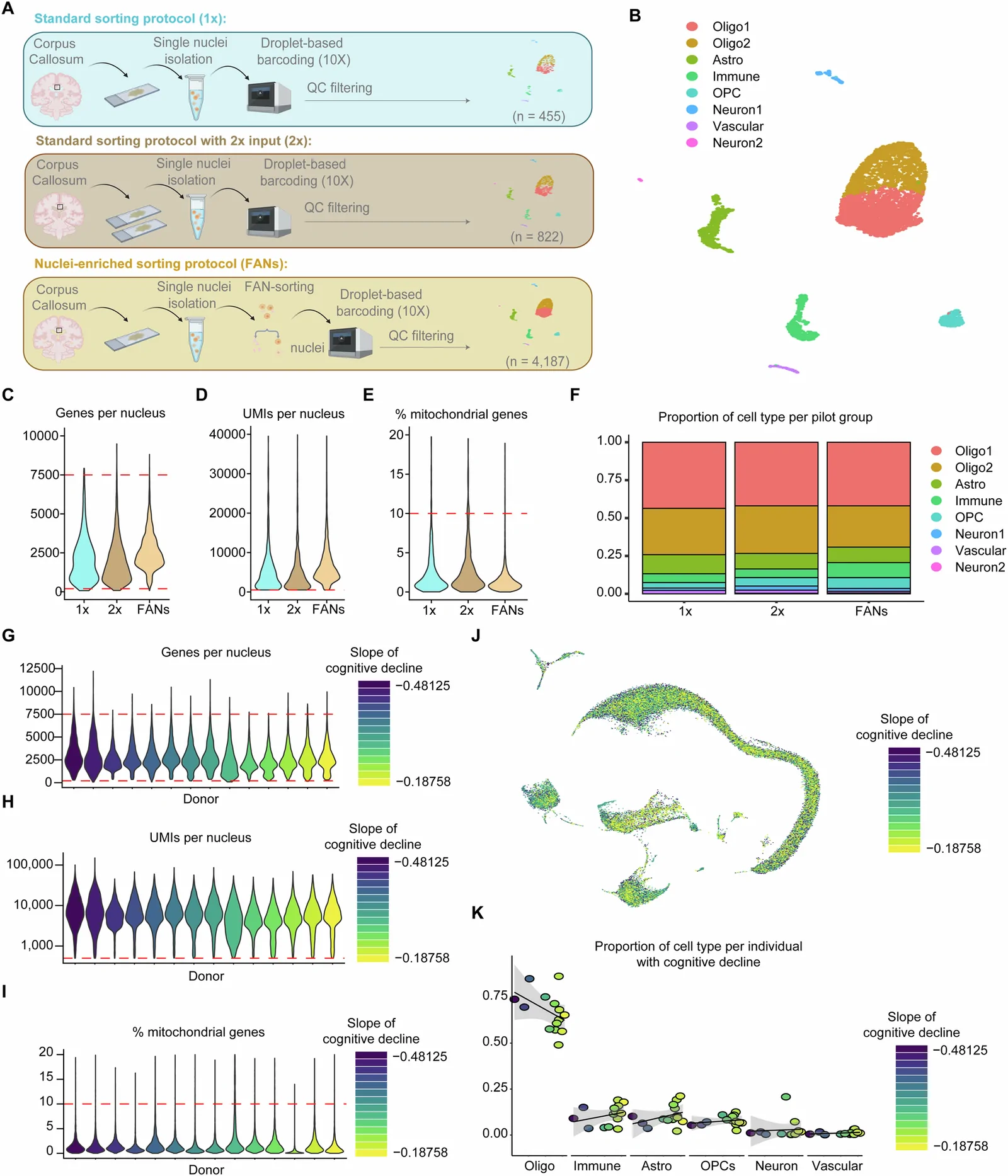
Optimization and quality control of single-nuclei RNA sequencing dataset. **A**. Schematic of pilot experiments to optimize the yield and quality of nuclei extracted from fresh-frozen human corpus callosum samples (created with BioRender.com): nuclei were isolated from aged human white matter tissue according to a standard protocol using the standard level of input material (1x), the same protocol with double the input material (2x), or including a nuclei sorting step (FANs). Libraries from each isolation method were then sequenced to assess differences in yield, cluster composition, and nuclei quality, and UMAPs of each method are shown with yield in parentheses. **B**. Uniform Manifold Approximation and Projection (UMAP) plot of the merged clustering of 3 pilot samples from (A). **C**. Genes per nucleus retrieved from pilot experiments showing a greater number of genes per nucleus after FAN-sorting of nuclei. Red dashed line represents quality control cutoff. **D**. Unique molecular identifiers (UMIs) per nucleus from the pilot experiment, with FAN-sorting improving nuclei quality. Red dashed line represents quality control cutoff. **E**. Percentage of mitochondrial genes expressed in pilot experimental datasets. Red dashed line represents quality control cutoff. **F**. Proportion of pilot experiment nuclei belonging to each cell cluster identified by preliminary clustering in (B). **G**. Genes per nucleus from sequenced LBC individuals, colored by slope of cognitive decline. Red dashed line represents quality control cutoff. **H**. UMIs per nucleus from sequenced LBC individuals, colored by slope of cognitive decline. Red dashed line represents quality control cutoff. n=14 individuals. **I**. Percentage of mitochondrial genes expressed in sequenced LBC individuals, colored by slope of cognitive decline. Red dashed line represents quality control cutoff. n=14 individuals. **J**. UMAP of LBC sequencing data colored by slope of cognitive decline. **K**. Proportion of nuclei belonging to each cell-type cluster in LBC individuals, correlated with and colored by slope of cognitive decline. Shaded area represents the 95% confidence interval. n=14 individuals.

To elucidate potential mechanisms underlying the myelin and axonal pathology associated with human cognitive decline, we optimized a single-nucleus RNA sequencing (snRNA-seq) protocol previously applied to adult and aged human white matter^30,31^ to enhance yield and nuclei quality while preserving relative abundance of cell types and clusters; fluorescence-activated nuclei sorting (FANS) improved yield tenfold without compromising nuclei quality or cell type proportions (Extended Data Fig. 2a−f). Implementing this pipeline on fresh-frozen corpus callosum samples from 14 individuals (Supplementary Table 1), we retained 45,437 high-quality nuclei after quality control (97% of all nuclei), with an average of 2,740 genes per nucleus and 7,706 unique molecular identifiers (UMIs) per nucleus (Extended Data Fig. 2g−i). After unbiased clustering, major cell types were identified through expression of canonical markers for oligodendrocytes (*PLP1*, *BCAS1*, *ENPP6*), immune cells (including microglia, macrophages and infiltrating cells; *PTPRC*, *CSF3R*, *DOCK8*), astrocytes (*GFAP*, *ALDH1A1*, *AQP1*), oligodendrocyte precursor cells (*PDGFRA*, *MMP16*, *CSPG4*), neurons (*SNAP25*, *GRIN1*, *CHRM2*) and endothelial/vascular cells (*CLDN5*, *MYO1B*, *SLC38A11*) (Fig. 2a,b). Mapping by cognitive status showed no emergence of novel clusters with cognitive decline (Extended Data Fig. 2j).

**Extended Data Fig. 3:**
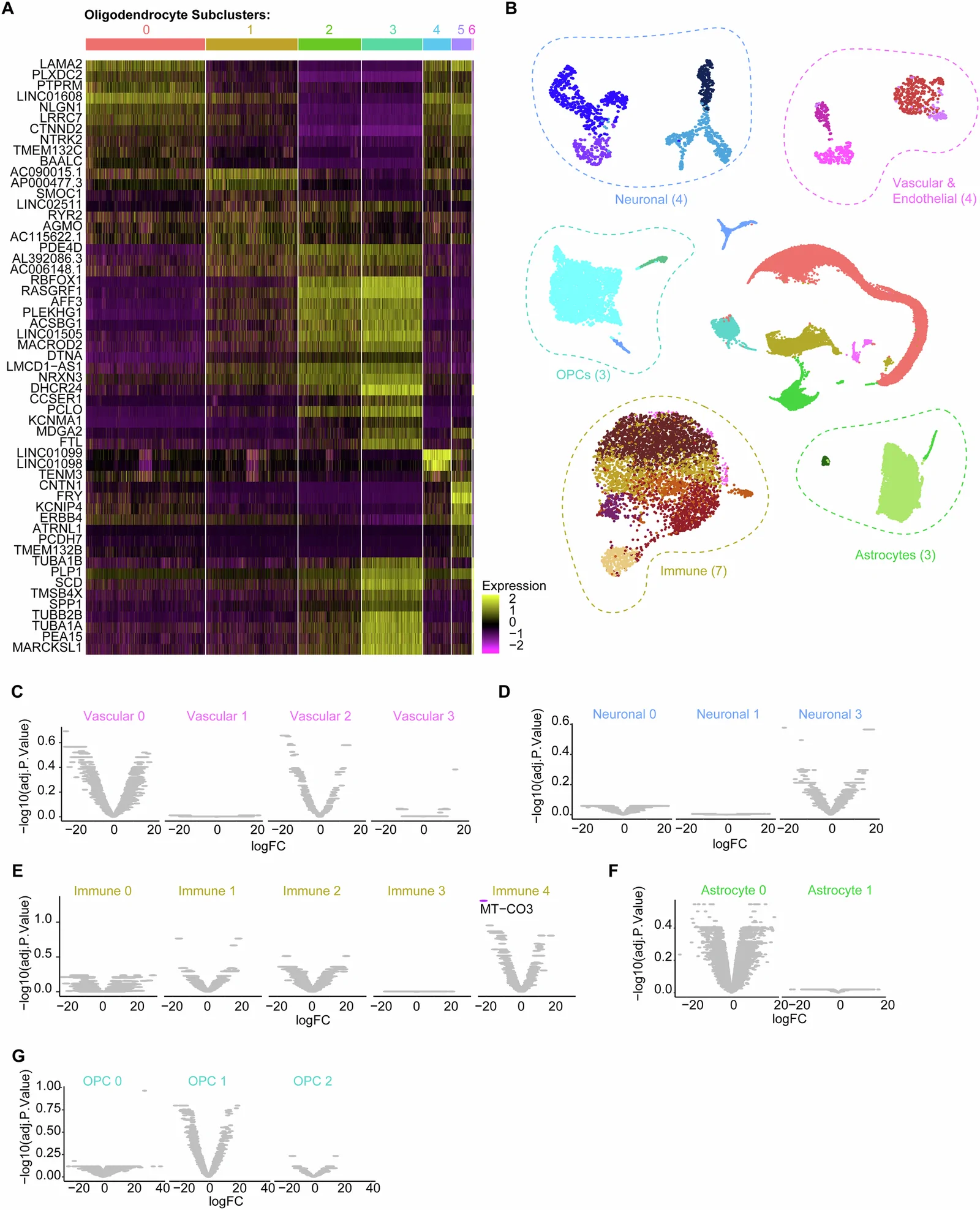
Cell-type sub-clustering transcriptional analysis of aged human white matter. **A**. Heatmap of top 3 genes enriched in each oligodendrocyte subcluster (0-6) across all sequenced LBC individuals. Genes are coloured according to their log-normalized, scaled, expression values, such that 0 represents mean expression across all cells and variance is 1. Yellow indicates high expression and purple indicates low expression. n=14 individuals. **B**. UMAPs of non-oligodendroglial cell type subclusters, showing subpopulations within each cell type. **C**. Volcano plots of vascular subclusters differentially expressed genes (DEGs; two-sided moderated *t*-tests with Benjamini-Hochberg multiple comparisons adjustment) by slope of cognitive decline across all sequenced LBC individuals. n=14 individuals. **D**. Volcano plots of neuronal subclusters DEGs (two-sided moderated *t*-tests with Benjamini-Hochberg multiple comparisons adjustment) by slope of cognitive decline across all sequenced LBC individuals. n=14 individuals. **E**. Volcano plots of immune subclusters DEGs (two-sided moderated *t*-tests with Benjamini-Hochberg multiple comparisons adjustment) by slope of cognitive decline across all sequenced LBC individuals. n=14 individuals. **F**. Volcano plots of astrocyte subclusters DEGs (two-sided moderated *t*-tests with Benjamini-Hochberg multiple comparisons adjustment) by slope of cognitive decline across all sequenced LBC individuals. n=14 individuals. **G**. Volcano plots of OPC subclusters DEGs (two-sided moderated *t*-tests with Benjamini-Hochberg multiple comparisons adjustment) by slope of cognitive decline across all sequenced LBC individuals. n=14 individuals.

Unexpectedly, we found that oligodendrocyte abundance was increased with more severe cognitive decline (Fig. 2c and Extended Data Fig. 2k). Although snRNA-seq of white matter is biased toward capturing oligodendrocytes, no other cell types showed associations of number or proportion of total cells with cognitive decline severity (Fig. 2c and Extended Data Fig. 2k) and were captured at frequencies expected from previous studies of human white matter^31,32^. Given that cell abundance from sequencing alone cannot reliably indicate altered numbers, immunofluorescence staining for Olig2 was performed, which validated an increased oligodendrocyte density with more severe cognitive decline (Fig. 2d,e). As shifts in oligodendrocyte heterogeneity have previously been associated with neurodegenerative disease^31,33–36^ and myelin thickening^37^, we next subclustered oligodendrocytes into seven subpopulations with distinct transcriptional signatures (Fig. 2f and Extended Data Fig. 3a). To understand how oligodendrocyte subclusters were altered with cognitive decline, pseudobulking and differential gene expression analyses were performed using edgeR and limma packages; genetic variance associated with technical factors (library size) and/or biological factors already known to influence cognition (age, sex, IQ at age 70 and histopathological evidence of Alzheimer’s disease or small vessel disease) were regressed out as covariates. Subclusters Oligo4 and Oligo5 showed the highest number of differentially expressed genes (DEGs) between mild and severe cognitive decline (167 and 113 genes, respectively; Supplementary Data 1), whereas all other oligodendrocyte subclusters showed either zero (Oligo1, Oligo2, Oligo3, Oligo6) or two (Oligo0) DEGs. With more severe cognitive decline, Oligo4 upregulated genes implicated in neurodevelopmental disorders with cognitive impairment (*RFX3-AS1*) and expressed by oligodendrocytes in the context of demyelination (*NRXN3*) as well as those involved in responses to DNA damage (*MND1*) and oxidative stress (*OXR1*) (Fig. 2g). In Oligo5, genes increased with more severe cognitive decline included those related to mitochondrial function (*MT-CO1*) and oligodendrocyte differentiation (*OPALIN*), whereas downregulated genes related to lipid production (*CERS6*), heat shock responses (*HSPA4L*, *HSPE1*) and autophagy (*SQSTM1*) (Fig. 2h). Moreover, in comparison to other oligodendrocyte subclusters, Oligo4 and Oligo5 were enriched for genes previously detected in oligodendrocytes in Alzheimer’s disease, Parkinson’s disease or multiple sclerosis, including *LINC01099*, *FRY*, *KCNIP4* and *LAMA2* (refs. ^33,38–40^) (Fig. 2i−l). Of note, subclustering and differential gene expression analysis revealed *MT-CO3* to be the only DEG enriched in a non-oligodendroglial cluster with cognitive decline, in an immune subcluster (Extended Data Fig. 3b−g). Ingenuity Pathway Analysis (IPA) of DEGs altered with cognitive decline in Oligo4 and Oligo5 was then performed to identify upstream regulators. Of all the upstream pathway regulators identified as significant by IPA (|*Z*| > 2), the only one shared across both Oligo4 and Oligo5 was NRF2 (*Z* = 2.217 and *Z* = 2.183 for Oligo4 and Oligo5, respectively), which was predicted to be downregulated with more severe cognitive decline in both subpopulations (Supplementary Data 1). This was of interest as global knockout of the gene encoding NRF2 (*Nfe2l2*) worsens cognitive impairment in mouse models of aging^41–43^, Alzheimer’s disease^44–47^, vascular dementia^48^ and infection^49^, yet the contributing cell types are unclear. We next assessed NRF2 expression by oligodendrocytes via immunostaining, which validated that the proportion of Olig2^+^ cells expressing NRF2 protein was decreased with severe cognitive decline (Fig. 2m,n).

We found no statistical relationship between oligodendrocyte densities nor NRF2^+^ oligodendrocyte proportions and postmortem interval, age, sex, age 11 IQ, cognition at age 70, extrapolated cognition at death or neuropathological indices of Alzheimer’s disease or small vessel disease (Fig. 2o). However, there was a significant correlation between oligodendrocyte densities and the proportion of NRF2^+^ oligodendrocytes (Fig. 2o). To assess whether these responses are functionally linked, we asked whether oligodendrocyte accumulation alone would be sufficient to induce downregulation of NRF2. We data-mined a transcriptional dataset in a mouse model where oligodendrocytes accumulate in excess (*Olig2-*Cre;*Tfeb*^fl/fl^) consequent to blocked apoptosis^50^. *Nfe2l2* was found to be significantly downregulated in *Olig2*-Cre;*Tfeb*^fl/fl^ oligodendroglia versus floxed control mice (*P* = 2.6 × 10^−6^). These data indicate that accumulation of oligodendrocytes is sufficient to downregulate NRF2 gene expression (Fig. 2p). Altogether, these results indicate that aged humans with more severe cognitive decline have an enrichment of oligodendrocytes with reduced NRF2 expression.

### Reduced NRF2 expression in aged mouse oligodendrocytes attenuates cognitive improvement over time and recapitulates neuropathology associated with human cognitive decline

**Fig. 3:**
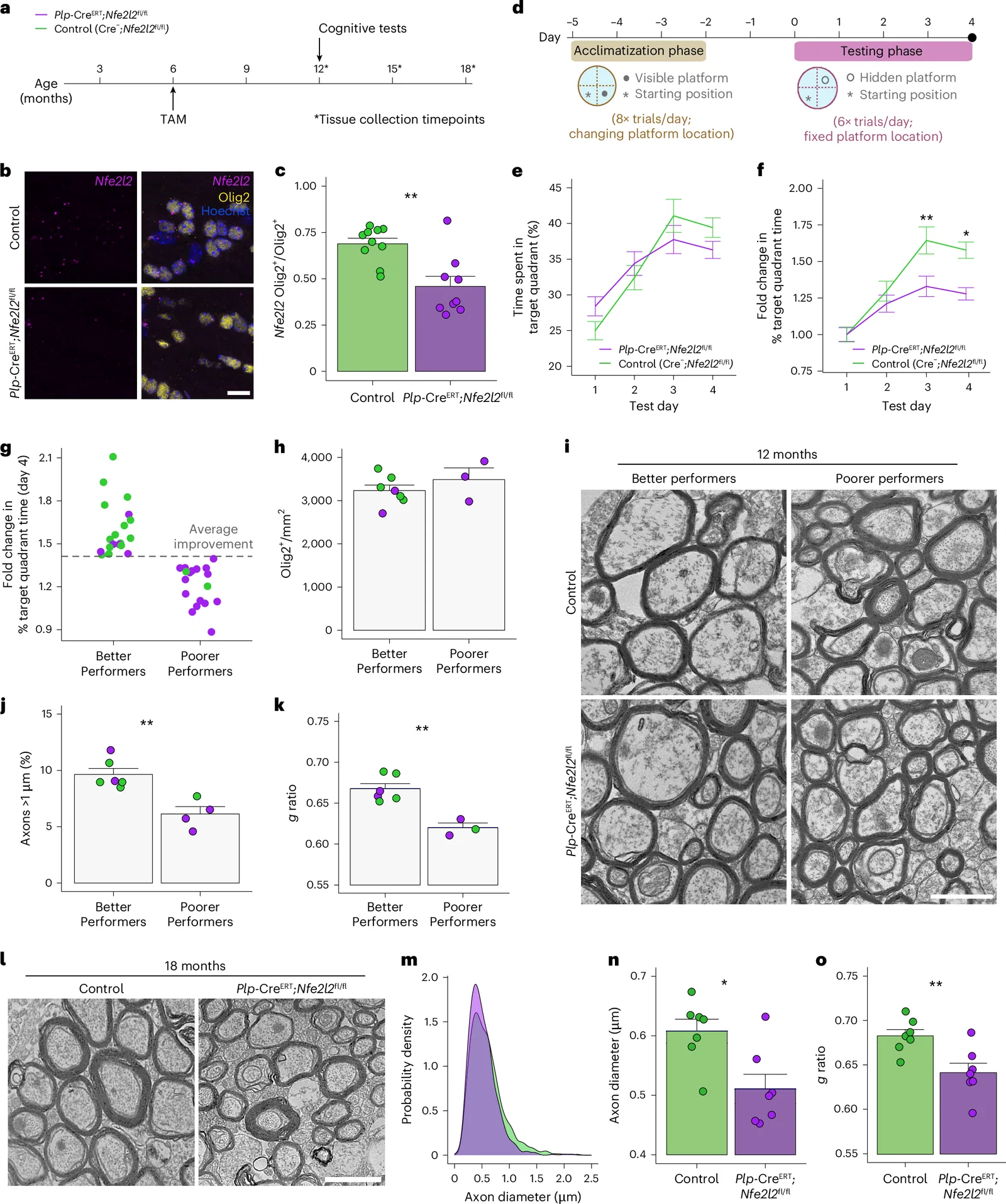
Loss of oligodendroglial NRF2 blunts cognitive improvement over time and exacerbates neuropathology associated with poor cognitive performance in aging. **a**, Experimental timeline of *Plp-*Cre^ERT^;*Nfe2l2*^fl/fl^ animals. **b**, Representative images of *Nfe2l2* probe (RNAscope) and Olig2 staining in a 12-month-old *Plp-*Cre^ERT^;*Nfe2l2*^fl/fl^ conditional knockout and control animal. Scale bar, 10 µm. The experiment was carried out on 19 animals. **c**, Average proportion of Olig2^+^ nuclei with *Nfe2l2* puncta in 12−15-month-old *Plp-*Cre^ERT^;*Nfe2l2*^fl/fl^ conditional knockout and control animals ± s.e.m. Two-sided unpaired Student’s *t*-test, *P* = 0.0029, *n* = 19 animals. **d**, Schematic of the MWM acclimatization and testing phases. **e**, Percentage of time spent in the target (platform-containing) quadrant of the MWM ± s.e.m. at 12 months of age. Two-way ANOVA with Sidak’s correction for multiple comparisons, with significant main effect of time (*P* = 2.32 × 10^−12^) and a significant interaction between time and genotype (*P* = 0.043) but no main effect of genotype (*P* = 0.86) or any significant differences on any individual day (*n* = 38 animals). **f**, Individual fold change in the percentage time spent in the target quadrant over the test duration ± s.e.m. at 12 months of age. Two-way ANOVA with Sidak’s correction for multiple comparisons, with a significant main effect of time (*P* = 1.02 × 10^−12^), a significant main effect of genotype (*P* = 0.0032) and a significant interaction between the two (*P* = 0.0091). Days 3 and 4 showed significant differences between genotypes (*P* = 0.042 and *P* = 0.0006, respectively) (*n* = 38 animals). **g**, Raw data of 12-month animals’ fold change in percentage time spent in target quadrant on day 4, showing animals split between higher than average fold change performance (‘better performers’) versus animals with lower than average fold change performance (‘poorer performers’), with average fold change (1.41) denoted with a dotted line (*n* = 38 animals). **h**, Average Olig2^+^ density (per mm^2^) at 12 months ± s.e.m., based on performance metrics. Purple dots indicate conditional knockout animals; green dots indicate controls. Two-sided unpaired Student’s *t*-test, *P* = 0.46, *n* = 10 animals. **i**, Representative images of corpus callosum at 12 months in control versus conditional knockout animals, split by performance. Scale bar, 1 µm. The experiment was carried out on 10 animals. **j**, Percentage of large-diameter axons (>1 µm) ± s.e.m. in better versus poorer performers tested at 12 months of age. Purple dots indicate conditional knockout animals; green dots indicate controls. Two-sided unpaired Student’s *t*-test, *P* = 0.0048, *n* = 10 animals. **k**, Mean *g* ratio ± s.e.m. in better versus poorer performers tested at 12 months of age. Two-sided unpaired Student’s *t*-test, *P* = 0.0013. Purple dots indicate conditional knockout animals; green dots indicate controls (*n* = 9 animals). **l**, Representative electron micrographs of myelinated axons in the corpus callosum from 18-month-old control versus *Plp-*Cre^ERT^;*Nfe2l2*^fl/fl^ animals. Scale bar, 1 µm. The experiment was carried out on 14 animals. **m**, Probability density distribution of the frequency histogram for myelinated axon diameters in the corpus callosum of 18-month-old control *Nfe2l2*^fl/fl^ (green) versus *Plp-*Cre^ERT^;*Nfe2l2*^fl/fl^ (purple) animals (*n* = 14 animals). **n**, Average axon diameter ± s.e.m. in the corpus callosum of 18-month-old control *Nfe2l2*^fl/fl^ (green) versus *Plp-*Cre^ERT^;*Nfe2l2*^fl/fl^ (purple) animals. Two-sided unpaired Student’s *t*-test, *P* = 0.011, *n* = 14 animals. **o**, Average myelin *g* ratio ± s.e.m. in the corpus callosum of 18-month-old *Nfe2l2*^fl/fl^ control (green) versus *Plp-*Cre^ERT^;*Nfe2l2*^fl/fl^ (purple) animals. Two-sided unpaired Student’s *t*-test, *P* = 0.0079, *n* = 14 animals. TAM, tamoxifen.

To elucidate the functional consequences of NRF2 downregulation in oligodendrocytes on myelin and axonal health in aging, we generated an oligodendrocyte-specific inducible conditional knockout of NRF2 (*Plp-*Cre^ERT^;*Nfe2l2*^fl/fl^) (Fig. 3a). Recombination was induced at 6 months of age, by which age the majority of oligodendrogenesis has occurred^51,52^, and was confirmed by RNAscope in 12−15-month-old aged animals (Fig. 3b,c). Behavioral tests were performed at 12 months of age, when wild-type mice begin to show impairment in learning and memory^6^.

**Extended Data Fig. 4:**
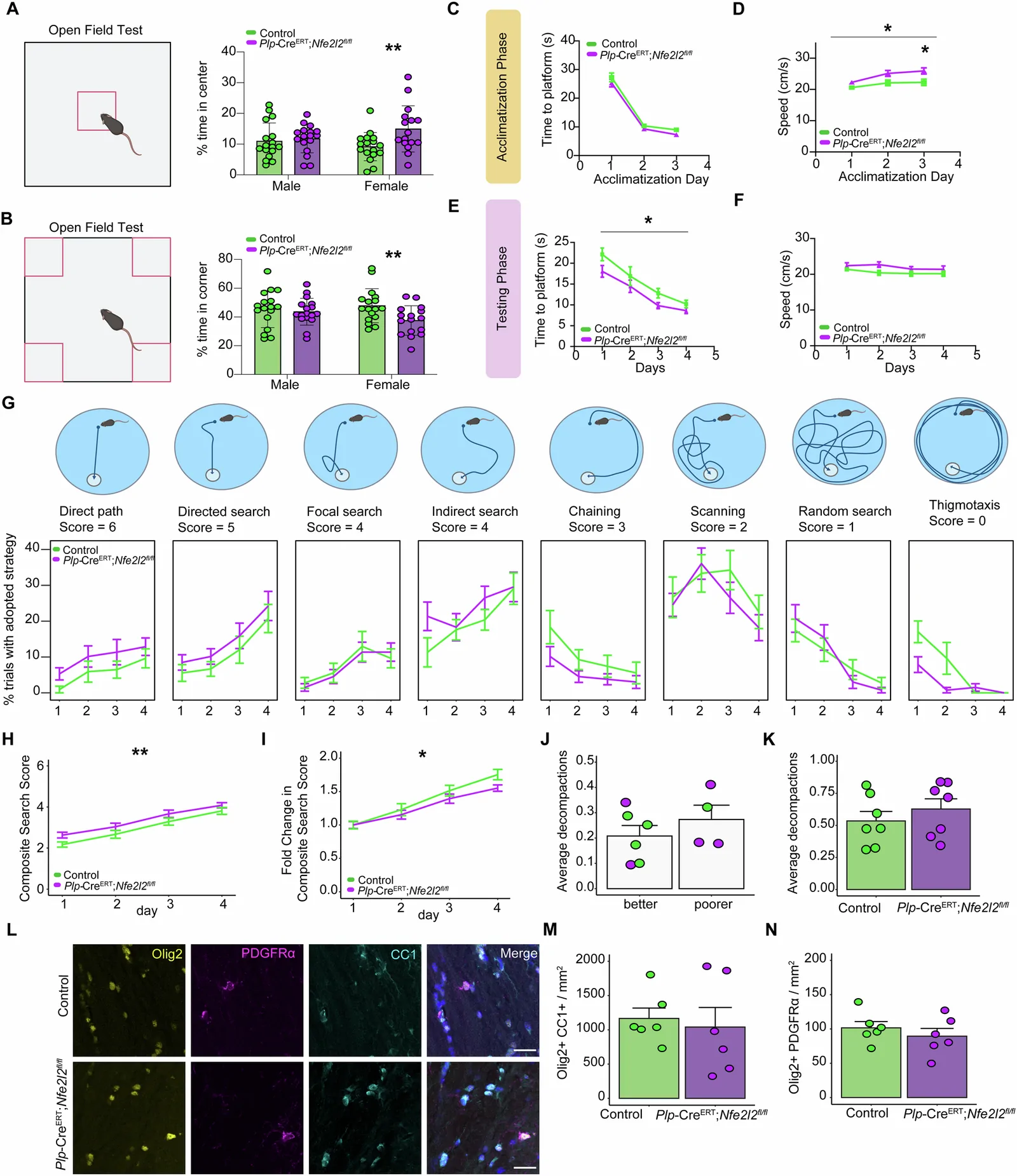
Oligodendrocyte-specific NRF2 conditional knockout cognitive testing and neuropathological assessments in aging. **A**. Percentage of time spent in the center of the Open Field test ± SEM by *Nfe2l2*^fl/fl^ (green) vs *Plp*-Cre;*Nfe2l2*^fl/fl^ (purple) animals. Two-Way ANOVA tested multiple comparisons (effect of genotype was significant; *P*=0.022) and Fisher’s LSD test tested within-sex comparisons, showing female conditional knockout mice spent significantly more time in center than controls (*P*=0.0054), with no difference between males (*P*=0.68). n=67 animals. Schematic was created with BioRender.com. **B**. Percentage of time spent in the corner areas of the Open Field test ± SEM by *Nfe2l2*^fl/fl^ (green) vs *Plp*-Cre;*Nfe2l2*^fl/fl^ (purple) animals. Two-Way ANOVA tested for multiple comparisons (effect of genotype was not significant; *P*=0.0714), and Fisher’s LSD test tested within-sex comparisons, showing female conditional knockout mice spent significantly less time in corners (*P*=0.0090), with no difference between males (*P*=0.64). n=67 animals. Schematic was created with BioRender.com. **C**. Latency to platform ± SEM for male control *Nfe2l2*^fl/fl^ (green) and *Plp*-Cre;*Nfe2l2*^fl/fl^ (purple) animals in the acclimatization phase of the Morris Water Maze. Two-Way ANOVA with Sidak’s post-hoc tested for multiple comparisons: effect of time was significant (p<1x10^−15^) but no main effect of genotype (*P*=0.089). No significant differences between genotype on any individual day of testing. n=38 animals. **D**. Swimming speed (cm/s) ± SEM for male control *Nfe2l2*^fl/fl^ (green) and *Plp*-Cre;*Nfe2l2*^fl/fl^ (purple) animals in the acclimatization phase of the Morris Water Maze. Two-Way ANOVA with Sidak’s post-hoc tested for multiple comparisons: effect of time was significant *(P*=5.41x10^−6^), effect of genotype was significant (*P*=0.0157). Conditional knockouts swam significantly faster on day 3 (*P*=0.0453). n=38 animals. **E**. Latency to platform (s) ± SEM for male control *Nfe2l2*^fl/fl^ (green) and *Plp*-Cre;*Nfe2l2*^fl/fl^ (purple) animals in the testing phase of the Morris Water Maze. Two-Way ANOVA with Sidak’s post-hoc tested for multiple comparisons: effect of time was significant (*P*=2.55x10^−12^), effect of genotype was significant (*P*=0.036). No significant differences between genotype on any individual day of testing. n=38 animals. **F**.Swimming speed (cm/s) ± SEM for male control *Nfe2l2*^fl/fl^ (green) and *Plp*-Cre;*Nfe2l2*^fl/fl^ (purple) animals in the testing phase of the Morris Water Maze. Two-Way ANOVA with Sidak’s post-hoc tested for multiple comparisons: effect of time was significant (*P*=0.0178), effect of genotype was not significant (*P*=0.15). No significant differences between genotype on any individual day of testing. n=38 animals. **G**. Search strategy categorization and quantification of percentage time with adopted strategy ± SEM in *Plp*-Cre;*Nfe2l2*^fl/fl^ animals (purple) versus *Nfe2l2*^fl/fl^ control animals (green) on each test phase day. n=38 animals. **H**. Composite search score (indicating directness of path taken) ± SEM on each day in the test phase by *Plp*-Cre;*Nfe2l2*^fl/fl^ animals (purple) versus control *Nfe2l2*^fl/fl^ animals (green). Two-Way ANOVA with Tukey’s post-hoc tested multiple comparisons: effect of age was significant (*P*<1x10^−15^), effect of genotype was significant (*P*=0.00162). n=38 animals. **I**. Fold-change in the composite search scores on each day in the test phase ± SEM by *Plp*-Cre;*Nfe2l2*^fl/fl^ animals (purple) versus *Nfe2l2*^fl/fl^ control animals (green). Two-Way ANOVA with Tukey’s post-hoc tested multiple comparisons: effect of age was significant (*P*<1x10^−15^), effect of genotype was significant (*P*=0.0409). n=38 animals. **J**. Average number of decompacted fibre bundles in myelinated axons ± SEM between better versus poorer performers at 12 months. Purple dots represent *Plp*-Cre;*Nfe2l2*^fl/fl^ animals and green dots represent *Nfe2l2*^fl/fl^ control animals. Student’s *t*-test, *P=*0.39. n=10 animals. **K**. Average number of decompacted fibre bundles in myelinated axons ± SEM between *Plp*-Cre;*Nfe2l2*^fl/fl^ animals and *Nfe2l2*^fl/fl^ control animals at 18 months. Two-sided, unpaired Student’s *t*-test, *P*=0.41. n=14 animals. **L**. Representative zoom-in images of oligodendrocyte precursors (PDGFRα+Olig2+) and mature oligodendrocytes (CC1+Olig2+) in the corpus callosum of aged *Nfe2l2*^fl/fl^ (control; top) versus *Plp-*Cre^ERT^*;Nfe2l2*^fl/fl^ (bottom) animals. Scale bar, 50 µm. The experiment was repeated on 12 animals. **M**. Density of mature oligodendrocytes (CC1+Olig2+ cells) ± SEM in the corpus callosum of aged control (green) versus *Plp-*Cre^ERT^*;Nfe2l2*^fl/fl^ (purple) animals. Two-sided, unpaired Student’s *t*-test, *P*=0.71, n=12 animals. **N**. Density of immature oligodendrocytes (PDGFRα+Olig2+ cells) ± SEM in the corpus callosum of aged control (green) versus *Plp-*Cre^ERT^*;Nfe2l2*^fl/fl^ (purple) animals. Two-sided, unpaired Student’s *t*-test, *P*=0.42, n=12 animals.

First, we performed the open field test to screen for any major motor or anxiety effects that may influence cognitive test readouts. Female conditional knockout mice showed confounding altered anxiety levels (Extended Data Fig. 4a,b) and were, thus, excluded from cognitive assay analysis. Next, the Morris water maze (MWM) was used to assess cognitive performance and learning over time, facilitating comparison to the longitudinal cognitive testing in the human cohort. Notably, the MWM has previously been shown to be sensitive to age-related cognitive impairment concomitant with alterations in corpus callosum myelin^5,6^. Mice underwent an acclimatization period of eight training trials per day for 3 days (four trial patterns, repeated twice), where they swam to a visible platform that changed quadrants between trials (Fig. 3d and Extended Data Fig. 4c,d). To then test development of spatial learning and memory, the mice underwent a testing phase of six trials per day for 4 days (three trial patterns, repeated twice), where visual cues enabled localization of the hidden platform in a fixed location (Fig. 3d). Over the course of the testing phase, all mice showed an increase in the percentage of time spent in the target quadrant containing the platform, indicating spatial learning and memory encoding (Fig. 3e). Although conditional knockout animals initially spent more time in the target quadrant in the first 2 days of the testing phase relative to controls, they spent relatively less time in the target quadrant by days 3 and 4 (Fig. 3e). Accordingly, when percentage time spent in the target quadrant was plotted as a fold change relative to testing day 1, controls showed an average improvement of 57.75%, whereas conditional knockouts improved by an average of only 27.82% (Fig. 3f). Conditional knockouts surprisingly had a shorter latency to reach the target quadrant versus controls over all test days (Extended Data Fig. 4e). As this did not reflect faster swim speed in the testing phase (Extended Data Fig. 4f), we next scored search strategy using an established rubric^53^, where more direct pathways to the platform are associated with higher scores (Extended Data Fig. 4g). Although conditional knockouts showed a more direct spatial strategy from day 1 (Extended Data Fig. 4h), they did not improve their spatial strategy over time as much as the controls (Extended Data Fig. 4i); notably, this did not represent reaching a ceiling level of improvement, as both conditional knockout and control animals only ever reached the maximum score of 6 in approximsately 10% of test trials (Extended Data Fig. 4g).

Although conditional knockouts showed attenuated cognitive improvement over time, we noted a large degree of individual variation in performance across both genotypes. To correlate individual cognitive performance with white matter histology as was done in humans, we binned animals based on whether performance over time was better or poorer than average (Fig. 3g). No significant difference was observed in oligodendrocyte densities between better versus poorer performers at 12 months (Fig. 3h). However, when tissue was assessed by electron microscopy, there were clear differences in ultra-structural features of myelinated axons between better and poorer performers (Fig. 3i). Both axon diameter and *g* ratio were significantly reduced in poorer performers (Fig. 3j,k), indicating that smaller axons and thicker myelin are correlates of worse cognitive performance in aging mice. In late aging (15−18 months), oligodendrocyte density and myelin compaction were unchanged (Extended Data Fig. 4j−n); however, myelinated axon diameter and *g* ratio were significantly reduced in conditional knockouts versus controls (Fig. 3l−o). This suggests that loss of NRF2 in oligodendrocytes exacerbates the neuropathological features associated with age-related cognitive decline. These findings demonstrate that NRF2 downregulation in oligodendrocytes is sufficient to blunt cognitive performance over time in aging and induce thicker myelin and a shift to smaller myelinated axon diameters, mirroring observations in severe cognitive decline in human aging. Altogether, these results indicate a novel role for oligodendroglial NRF2 in contributing to cognitive impairment in aging.

## Discussion

Here we identify the oligodendrocyte as a contributor to the neuropathology of cognitive decline with aging. Using a unique human brain bank from a cohort with lifelong individual trajectories of cognitive status complemented by functional modeling in transgenic mice, we reveal that oligodendrocyte dysfunction contributes to myelinated axon pathology and cognitive impairment over time in aging. This study suggests that oligodendrocytes are not always supportive of CNS function and cognition, revealing that they can become actively detrimental in aging.

We found that individuals with severe cognitive decline have a loss of myelin integrity, including abnormally thicker myelin on large-diameter axons and a shift toward smaller myelinated axon profiles. These may directly influence cognitive ability, as 85% of variance in human axon conduction velocity is predicted to be controlled by myelin thickness and axon size^54^. Nonetheless, our association of more myelin with worse cognitive decline is surprising, given that, in youth/adulthood, learning involves myelin production^1,55^, and cognitive deficits are typically associated with myelin loss^56,57^. This could reflect that aging-associated cognitive decline involves surpassing the amount of additional myelin which is beneficial. Indeed, excess myelin thickening is known to precede CNS neurodegeneration in young adults with multiple sclerosis^58^, in aged non-human primates^59^ and in mouse models of amyloidopathy^37,60–62^. Consistent with these findings, myelinated axons are recently recognized to be more vulnerable to degeneration than non-myelinated axons in human^24^ and mouse white matter^63^, indicating that pathological myelin is more deleterious for axons than no myelin at all. We build upon this by now identifying a particular vulnerability of myelinated axons of large diameter (>1 µm) in human aging, which preferentially show thicker myelin and decreased frequencies in more severe cognitive decline. This contrasts with previous work documenting decreased small-diameter myelinated axons in human white matter in aging versus youth^64–66^; we did not detect this phenomenon in association with cognitive decline, suggesting that small axon pathology may occur at an equal rate across aged individuals yet could have less impact on cognitive decline than large axon pathology.

We uncovered that oligodendrocyte dysfunction can be driven by NRF2 deficiency. Decreased expression of NRF2 in oligodendrocytes was observed in individuals with severe cognitive decline; modeling this in mice was functionally sufficient to cause thicker myelin and a shift toward myelination of smaller-diameter axons, and to blunt cognitive improvement over time in aging. NRF2 regulates the activation of hundreds of genes in a cell-type-specific manner, in turn influencing a wide range of cellular functions, including autophagy, antioxidant responses, proliferation and differentiation; here we identify a novel role for NRF2 in oligodendrocytes in regulating myelin thickness and myelinated axon health. Although the exact mechanism by which NRF2 regulates myelin thickness is unknown, the established interplay between NRF2 and autophagy in other cell and tissue systems suggests that this could contribute and would be consistent with autophagy disruption in oligodendrocytes leading to hypermyelination and subsequent neurodegeneration^67–69^. This complements the well-established role of NRF2 in cognition, where global NRF2 knockout exacerbates age-related cognitive deficits in several mouse models^41–49^; drug-induced NRF2 activation (using dimethyl fumarate) improves cognitive function in rodents and in multiple sclerosis^70–74^, yet the contributing cell type was undefined. Together with our findings, this highlights the possibility of repurposing NRF2 activators to mitigate oligodendrocyte dysfunction associated with cognitive decline in human aging.

There are likely several triggers of oligodendrocyte dysfunction and consequent myelin pathology in aging. Our data suggest that an increase in oligodendrocytes may occur upstream of NRF2 downregulation. Whether the increased oligodendrocytes in more severe cognitive decline reflects new generation in aging or enhanced generation earlier in life is unknown, although limited oligodendrogenesis in human aging^75^ may support the latter possibility. Our finding of a deleterious association of oligodendrocyte densities with cognitive status is unexpected, as new oligodendrocyte generation is required for learning during development and adulthood in mice^2–5^, and supraphysiological boosting of new oligodendrocytes in aged mice can attenuate age-related memory deficits^4,6,76^. Nevertheless, increased oligodendrocyte densities have been previously documented in human pathology—in the gray matter in Alzheimer’s disease^77^ and in the white matter after developmental insults^78^— although the functional status of these oligodendrocytes and the consequences of this increase remain unexplored in these contexts. Our findings suggest that, at least in the context of human aging, an increase in oligodendrocytes is associated with adoption of a state that is dysfunctional. There are likely also other upstream drivers of oligodendrocyte dysfunction and myelin pathology in aging; changes in the aging brain, such as inflammation and vascular pathology, are known modulators of NRF2 (refs. ^79,80^) and oligodendrocyte/myelin biology^81,82^ and may, therefore, contribute to cognitive decline. In addition, an established driver of increased myelin thickness is neuronal activity, which drives experience-dependent adaptive myelin remodeling to support learning^83,84^. Neuronal activity is suggested to increase in aging in some individuals with detrimental outcomes for lifespan^85^, and we speculate that this may also support maladaptive (excessive) myelination^86^ to contribute to cognitive decline. Aging humans often show greater activity in certain brain regions during cognitive tasks compared to younger individuals^87–89^, which may reflect an increase in neuronal activity in an attempt to compensate for cognitive decline. Investigation of other brain regions beyond the corpus callosum could, therefore, reveal additional cellular and molecular contributors to cognitive decline in aging.

Altogether, our study reveals the oligodendrocyte to be a contributor to the white matter neuropathology associated with cognitive decline in aging. This work, therefore, highlights oligodendrocytes and their NRF2 expression as novel therapeutic targets to help support cognitive health in aging.

## Methods

### LBC1936

Cognitive data and brain samples were obtained from LBC1936 (Medical Research Council Brain Bank, The University of Edinburgh), with ethics permission from the Multi-Centre Research Ethics Committee for Scotland (MREC/01/0/56), the Lothian Research Ethics Committee (LREC/2003/2/29) and the Scotland A Research Ethics Committee (07/MRE00/58). Participants are Scottish, of majority white European ancestry and include both sexes (gender data not requested). Clinical history and neuropathological examinations were provided by C. Smith (The University of Edinburgh). The use of LBC samples was in accordance with the terms of use for donor tissue and information.

### Cognitive testing

In 1947, participants of mean age 11 years underwent a group-administered 45-minute general intelligence test (Moray House Test (MHT) No. 12), which indicated concurrent validity with the standardized Stanford−Binet test of intelligence (*R* = 0.8)^90^. Individuals consented to re-testing at age 70 onwards^13^; of 1,091 participants, 1,017 had viable age 11 and age 70 test scores, enabling correlation between IQ in childhood and aging. In total, 866 participated beyond wave 1, of whom 816 had viable age 11 test scores, enabling correlation between cognitive decline in aging and age 11 IQ. Data from waves 1−5 were used in this study, where standardized cognitive tests were administered at each wave (approximately every 3 years), in addition to the MHT (No. 12) (Fig. 1a and Extended Data Fig. 1a). Of these, 10 tests were input to a latent growth curve model in a structural equation modeling framework to indicate latent general cognitive ability^14–16^, including matrix reasoning, block design, digit−symbol match and symbol search (Weschler Adult Intelligence Scale III); logical memory, digit span, verbal paired associates and digit backwards test (Weschler Memory Scale III); choice reaction time (UK Health and Lifestyle Survey); and inspection test (constructed using E-Prime software). Raw test results were scaled so that variances were in similar ranges across tests. Growth curve models for individual tests were specified and fit to the data using the lavaan (version 0.6.21), haven (version 2.5.5) and psych (version 2.6.5) packages in R (version 4.4.2), with slope weightings as the mean test interval (years) and using full information maximum likelihood estimation. A hierarchical model was fit in which cognitive test scores were assigned to cognitive domains (Extended Data Fig. 1a), and high correlations between domains were modeled as a hierarchical general cognitive factor (*g*)^14–16^, showing excellent fit to the data (Tucker−Lewis index (TLI) of 0.945, comparative fit index (CFI) of 0.946, standardized root mean square residual (SRMR) of 0.066 and root mean square error of approximation (RMSEA) of 0.033). Intercept and slope scores were extracted for each individual using the lavPredict function (lavaan package, version 0.6.21); the intercept represented *g* at age 70, and the slope represented annual decline from 70 to 82 years. All but one individual showed a negative rate of change in *g* (*S*_*g*_ < 0) beyond age 70, with the average decline per year being −0.26 s.d. *g* units. Trajectories declining more steeply than this average (that is, *S*_*g*_ < −0.26) were classed as ‘severe’, whereas those above average were classed as ‘mild’.

### Human brain tissue

Brain tissue was collected from a subset of consenting individuals^91^. Postmortem intervals ranged between 1 day and 4 days, during which cadavers were stored at 4−6 °C^92^. LBC1936 samples (79−85 years) and adult samples (34−49 years) were acquired from the LBC tissue bank or the Sudden Death Brain Bank under the Medical Research Council Edinburgh Brain and Tissue Bank (21/ES/0097) and approved by local ethical review boards in Edinburgh and at Unity Health Toronto (REB 23-088). For LBC1936, one hemisphere was drop fixed into 10% (w/v) formalin fixative for paraffin embedding and histochemistry, and the remaining hemisphere was sliced into 1-cm-thick coronal sections; 1-cm^3^ regions of interest were collected as tissue punches^91^ and processed for electron microscopy. Adjacent samples were snap frozen for transcriptomic analyses.

### Electron microscopy of human white matter

Fresh human postmortem brain tissue (1 cm^3^)^91^ was placed in 0.1 M phosphate buffer. Tissue was dissected (1 × 1 mm^3^) and fixed for 48 hours in 4% paraformaldehyde (PFA; Electron Microscopy Sciences, cat. no. 15710) and 2.5% (w/v) glutaraldehyde (Electron Microscopy Sciences, cat. no. 16310) in 0.1 M electron microscopy-grade phosphate buffer (pH 7.4) at 4 °C and then washed and stored in 0.1 M phosphate buffer for a minimum of 24 hours^92^. Samples were incubated in 1% (w/v) osmium tetroxide (Electron Microscopy Sciences, cat. no. 19152) in 0.1 M phosphate buffer for 30 minutes at room temperature, washed in 0.1 M phosphate buffer and then ddH_2_0 and dehydrated in graded ethanols, finishing in propylene oxide (Sigma-Aldrich, cat. no.56671-5ML-F). Sections were embedded in durcupan resin (Sigma-Aldrich, cat. no.44610-1EA) for 24 hours and polymerized for 48 hours at 56 °C. Ultra-thin sections (50 nm) were cut on an ultra-microtome (Leica, EM UC7) and stained with uranyl acetate and lead citrate, and grids were imaged on a JEOL JEM 1400 Plus transmission electron microscope. A minimum of 100 myelinated axons were traced for axon diameter and myelin thickness using Fiji ImageJ, assuming circularity (axon diameter = 2 × sqrt(axon area / Π)). Myelin thickness (*g* ratio) was calculated by (inner axon diameter / outer myelinated axon diameter). Large-diameter axons were categorized as being >1 µm, at which myelin is thickest under homeostasis^93^ and more vulnerable to increased thickness in disease^37^. Myelinated axons were quantified in an average area of 1,500 µm^2^. Mitochondrial area was measured from a minimum of 100 myelinated axons per donor and traced using the polygon tracing tool (Fiji ImageJ). Degenerating axons were defined as lacking visible pallor with intensities equivalent to cytoplasmic space. Seventeen lateral corpus callosum samples were available for electron microscopy analysis; due to poor tissue quality, two cases were excluded from myelinated axon density and four from myelin thickness analysis. Investigators were blinded to case history and cognitive trajectories throughout analysis.

### snRNA-seq

A pilot study optimized retrieval of high-quality nuclei for sequencing (Extended Data Fig. 2a−f), testing a previously established protocol (1)^30,31^, including twice the input material (2) or using FANS to isolate nuclei and exclude myelin debris (3). Fresh-frozen human tissue sections (20 µm) were cut at −20 °C and placed into ultra-low binding Eppendorf tubes on ice. Either five sections (protocols 1 and 3) or 10 sections (protocol 2) of 1 × 1 cm^3^ were utilized. Then, 200 µl of lysis buffer (1,700 µl of PURE buffer (Sigma-Aldrich, NUC-201-1KT), 17 µl of 0.1 M DTT (Thermo Fisher Scientific, cat. no. 15846582), 17 µl of 10% Triton-X (BioShop Canada, cat. no. TRX777.100) and 34 µl of SUPER RNAse inhibitor (Thermo Fisher Scientific, cat. no. AM2696)) was added to each tube and tissue homogenized on ice with needles of ascending gauge until a smooth consistency was achieved. Lysates were mixed with 360 µl of sucrose cushion medium (PURE 5 M sucrose solution (Sigma-Aldrich, NUC-201-1KT)) containing 11% PURE sucrose cushion buffer (Sigma-Aldrich, NUC-201-1KT), 0.11% DTT (Thermo Fisher Scientific, cat. no. 15846582) and 0.02% RNAse inhibitors (Thermo Fisher Scientific, cat. no. AM2696) and then passed through a 30-µm strainer (Wolf Laboratories, 04-004-2326). The filtrate was gently plated on 200 µl of the sucrose cushion medium, and samples were centrifuged for 45 minutes at 16,100*g* at 4 °C. The pellet was resuspended in 100 µl of elution buffer, which, for protocols 1 and 2, constituted 7 ml of PURE storage buffer (Sigma-Aldrich, NUC-201-1KT) containing 70 µl of RNAse inhibitor (Thermo Fisher Scientific, cat. no. AM2696) and, for protocol 3, constituted sterile PBS (Thermo Fisher Scientific, cat. no. BP399-20) with 1% fatty-acid-free BSA (Sigma-Aldrich, cat. no. A8806-1G), 2 mM EDTA and 1% RNAse inhibitor (Thermo Fisher Scientific, cat. no. AM2696). Resuspended samples were moved into a fresh tube, centrifuged for 5 minutes at 1,000*g* at 4 °C and pellet resuspended in 200 µl of elution buffer. DAPI (1:1,000) was added in to protocol 3. Samples were centrifuged for 5 minutes at 1,000*g* at 4 °C and resuspended in 50−150 µl of elution buffer. For protocols 1 and 2, nuclei were counted by mixing with trypan blue (1:1) using the Bio-Rad Cell Counter TC200. Nuclei concentration was standardized to 100,000 nuclei per milliliter using the elution solution and processed for snRNA-seq. Protocol 3 was FAN-sorted using a BD FACSAria II cytometer running BD FACSDiva software (version 8.0.1), gating on DAPI^+^ nuclei; 60,000 events were captured into elution solution, the sample centrifuged at 750*g* for 5 minutes, pellets resuspended in 50 µl of elution solution and standardized to 100,000 nuclei per milliliter. As sorting nuclei provided the best results, protocol 3 was utilized when running the LBC samples as singular cases. Eight cases were processed as separate samples per day, with 2 days of processing (total 16 cases), filling a 10x Genomics Chromium Single Cell 3′ chip. Best efforts were taken to balance each batch for sex, age and degree of cognitive decline. In total, 52,000−60,000 nuclei were collected per case during the FANS step, with final 10× loading of 20,000 nuclei per case.

### Library preparation and next-generation whole-transcriptome sequencing

Approximately 20,000 nuclei per sample were loaded onto a 10x Genomics Chromium Single Cell 3′ chip. Then, 10x Genomics v3 beads, gel beads-in-emulsion (GEM) kits and library kits were used as per the manufacturer’s instructions to generate Chromium Single Cell 3′ Gene Expression (GEX) libraries. Library concentrations and quality were tested with an Agilent TapeStation (HS D5000). Two of 16 samples had cDNA libraries with a concentration lower than 6 ng µl^−1^ and were, therefore, of poor quality; all other samples had libraries between 6.37 ng µl^−1^ and 13.8 ng µl^−1^. Libraries were assessed for size distribution on the Agilent Bioanalyzer (Agilent Technologies, G2939AA) with the DNA HS Kit (5067-4626) and quantified using the Qubit 2.0 Fluorometer (Thermo Fisher Scientific, Q32866) and the Qubit dsDNA HS assay kit (Q32851). Library molarity for sequencing was calculated using the Qubit dsDNA quantification results and the fragment size information from the Bioanalyzer results. Sequencing was performed on the NextSeq 2000 platform (Illumina, SY-415-1002) using the NextSeq 1000/2000 P3 Reagents (100 cycles) v3 kit (20040559). Libraries were pooled in equimolar quantities, and PhiX Control v3 (FC-110-3001) library was spiked into the three runs at a concentration of approximately 1% to help with cluster resolution and facilitate troubleshooting. All 16 libraries were initially run on one P3 flow cell, and resulting nuclei quality was confirmed. Two of 16 samples with significantly lower nuclei counts were flagged as having poor library quality and removed from further analysis. The remaining 14 libraries were sequenced on an additional two P3 flow cells. The three flow cells generated 180 GB per run (approximately 540 GB total) with approximately 80% of clusters passing filter during sequencing and 93% of the data in each run being >Q30. Base call data (generated by NextSeq 1000/2000 Control Software version 1.5.0.42699) were transferred to a computer running Ubuntu (version 20.04.2 LTS), and Cell Ranger (version 7.1.0) generated FASTQ files via demultiplexing raw base call files. FASTQ files were demultiplexed and aligned to the human reference genome GRCh38 using 10x Genomics’ Cell Ranger pipeline (version 7.1.0) on a high-performance computing cluster. Prior to any quality control, 46,988 nuclei were retrieved with an average of 2,715 genes per nucleus, 7,750 UMIs per nucleus and an average mitochondrial percentage per nucleus of 2.22%. The quality of nuclei retrieved did not significantly differ across samples (Extended Data Fig. 2g−i).

### snRNA-seq analysis

Filtered feature matrices resulting from the alignment of each sample with Cell Ranger (version 7.1.0) were read into R (version 4.4.2) using the Read10X function (Seurat package, version 5.2.1), and Seurat objects for each sample were created with the CreateSeuratObject (SeuratObject package, version 5.1.0). Each Seurat object was visually inspected for number of features, counts and mitochondrial gene percentages. Filtering thresholds across Seurat objects were chosen such that only nuclei with a feature number between 200 and 7,500 and mitochondrial gene percentages less than 10% were included, as recommended for human nuclei^94^. No nucleus had a UMI less than the recommended cutoff of 500, with the average UMI count per nucleus being 7,750 before filtering. After quality control filtering, we retained 45,437 nuclei, with an average of 2,740 genes and 7,705 UMIs per nucleus. Seurat objects were normalized, and the 2,000 most variably expressed features across objects were selected as integration anchors to combine the samples into one Seurat object. The resulting integrated object was scaled, and linear dimensional reduction via principal component analysis (PCA) was performed. Elbow plots and JackStraw plots visualized the contributions of each principal component, and the top 11 were chosen as input to cluster the nuclei in a uniform manifold approximation and projection (UMAP) space. For primary cell type annotation, a resolution value of 0.01 was used.

### Cell type annotation and subsetting

Canonical features of expected populations (*PLP1*, *BCAS1* and *ENPP6* for oligodendrocytes; *GFAP*, *ALDH1A1* and *AQP1* for astrocytes; *PTPRC*, *CSF3R* and *DOCK8* for immune cells; *PDGFRA*, *MMP16* and *CSPG4* for oligodendrocyte precursor cells; *SNAP25*, *GRIN1* and *CHRM2* for neurons; and *CLDN5*, *MYO1B* and *SLC38A11* for endothelial/vascular cells) were visualized in the combined UMAP by feature plots. Cell type identities were confirmed via inspection of top-expressed genes within each cluster. To analyze cell type subpopulations, we subset the combined Seurat object by each cell type cluster, and the resulting objects underwent scaling and linear dimensional reduction via PCA. An appropriate dimensionality of the data was chosen via inspection of the elbow plots. For all subset populations, a dimensionality of 8 was chosen for further processing. UMAPs were run with the input dimension set to 8, and single-nucleus neighbors and clusters were found via *k*-means clustering. Resolution was determined through trialing progressively higher resolutions for each cell type and inspecting subcluster separation (visualized via heatmaps of the top-expressed genes using Model-based Analytics of Single-cell Transcriptomics (MAST)) and alignment with known biological variation in cell types. Oligodendrocyte subclusters were well separated transcriptionally (Extended Data Fig. 3a), recapitulating the approximate number seen previously^30,31,34,95^, including in human^33^. The final resolutions used for each cell type subset were 0.2 for oligodendrocytes (seven clusters), 0.07 for oligodendrocyte precursor cells (three clusters), 0.1 for neurons (four clusters), 0.02 for astrocytes (three clusters), 0.2 for immune cells (seven clusters) and 0.05 for endothelial/vascular cells (four clusters) (Extended Data Fig. 3b).

### Differential gene expression analysis

To analyze differential gene expression, pseudobulk count matrices of the sequencing data were generated using the limma and voom pipeline within the limma (version 3.56.2) and edgeR (version 3.42.4) packages in R (version 4.4.2). For each cell type, pseudobulk groups corresponding to cell type subclusters for each individual were defined. The pseudobulk count matrix was normalized to counts per million per gene and filtered such that only genes with at least one read in at least 80% of samples were retained. The library sizes were reset to reflect the refined dataset, and raw library sizes were converted into normalized effective library sizes via the calcNormFactors function in edgeR (version 3.42.4). Only clusters found in more than half of the sequenced cases were analyzed for DEGs, excluding four subpopulations (Neuron2, Astro2 and Immune 5/Immune6). To analyze DEGs in oligodendrocyte subclusters, contrast matrices were defined to compare each enriched subpopulation against all other oligodendrocyte subpopulations. These design matrices were used as input when readying count data for linear modeling using the voom function of the limma (version 3.56.2) package, and resulting voom plots were inspected to check that gene filtering thresholds were stringent enough to remove poorly predictive data. Voom output on the filtered dataset was fit to a linear model (lmFit function, ‘stats’ package (version 4.3.1)) adhering to the design matrix defined, and estimated coefficients were computed (contrasts.fit function, limma package (version 3.56.2)). Log-odds of differential expression were generated via empirical Bayes moderation of the standard errors toward a global value (eBayes function, limma package (version 3.56.2)). To analyze differential gene expression in cell type subclusters across cognitive decline scores, design matrices were generated that included cognitive decline slopes and the following covariates: absolute cognitive score at age 70, neuropathological indications of Alzheimer’s disease or small vessel disease, age, sex and size of the cluster per donor; this regressed out variability associated with biological processes distinct from the rate of cognitive decline, which drastically reduced the number of DEGs passing the threshold of adjusted *P* < 0.05.

### Pathway analysis

Pathway analysis was implemented using IPA software. For analysis of Oligo4 and Oligo5 across the spectrum of cognitive decline, a filtration cutoff of *P* < 0.1 was applied in order to include a sufficient number of genes required for statistical confidence. Predicted upstream regulators were only considered for follow-up analysis if they were not included as chemical drugs or toxicants and had an ‘activated *z*-score’ (indicative of significant pathway activity) greater than absolute 2.

### SCoRe imaging

SCoRe imaging was carried out using a Leica Stellaris 5 confocal microscope with an RT85/15 beamsplitter set to capture reflectance. Laser wavelengths were set to 488 nm, 561 nm and 638 nm, and detectors centered around these wavelengths at the minimum physical opening threshold of 5 nm. For the first image in each sample, laser power and gain were increased until the first positive saturated pixel was detected, and then image settings were fixed. For the human tissue, formalin-fixed paraffin-embedded (FFPE) sections were deparaffinized, and eight technical replicate images were taken with a ×63 oil objective (numerical aperture 1.4; HC PL APO CS2) at systematic random sampling intervals across the section. The readout from each channel was merged into one composite image and converted to an RGB 8-bit image, and then the percentage area of signal above threshold background was quantified using a fixed threshold (value 9, based on the average default threshold across the first three samples imaged) in Fiji ImageJ.

### Immunofluorescent staining of human white matter

FFPE sections (6 µm) of lateral corpus callosum were dewaxed at 60 °C for a minimum of 2 hours and then rehydrated in xylene (2 × 10 minutes) and 5-minute incubations in ethanol (100%, 100%, 95%, 80%, 70%). Samples were microwaved in hot (approximately 95 °C) 0.1 M sodium citrate buffer (pH 6; Vector Laboratories, cat. no. H-3300-250, diluted in ddH_2_O) for 5−10 minutes, placed in a 60 °C oven for 30 minutes and then cooled at room temperature. Endogenous peroxidase and alkaline phosphatase blocking solution (Bloxall; Novus Biologicals, cat. no. Sp-6000-NB) was applied for 10 minutes, before a 1-hour block in 10% normal horse serum with 0.3% Tween 20 (Thermo Fisher Scientific, cat. no. BP337100) in 0.1 M TBS (Thermo Fisher Scientific, cat. no. BP24711). Primary antibodies were applied in a humid chamber overnight at room temperature. Tissue was washed (3 × 5 minutes in 0.1 M TBS), incubated for 1 hour with an HRP-conjugated secondary antibody (Vector Laboratories) and then washed again and incubated with a tyramide Opal-conjugated secondary antibody diluted in amplification diluent for 10 minutes (Akoya Biosciences, cat. no. FP1135). Tissue was washed, and Hoechst (1:1,000; Thermo Fisher Scientific, cat. no. H1399, lot 2936197) was added into the penultimate TBS wash. Sections were dried, mounted with Fluoromount G (Thermo Fisher Scientific, cat, no. 00-4958-02) and coverslipped (Thermo Fisher Scientific, cat. no. 12541033CA). For oligodendrocyte detection, a rabbit primary antibody against Olig2 (1:100; Sigma-Aldrich, cat. no. AB9610, RRID: AB_570666, lot 3857662), a secondary horse anti-rabbit HRP-ImmPress antibody (Vector Laboratories, cat. no. MP-7401, RRID: AB_2336529, lot ZK0830; used neat) and a tyramide Opal-570 fluorophore (1:100; Akoya Biosciences, cat. no. FP1488001KT, lot 231016009) were applied. For NRF2 and Olig2 double staining, mouse anti-Olig2 (1:100; Sigma-Aldrich, MABN50; lot 421151), horse anti-mouse HRP-ImmPress secondary antibody (Vector Laboratories, cat. no. MP-7402-15, lot ZL1001) and tyramide Opal-570 fluorophore (1:100; Akoya Biosciences, cat. no. FP1488001KT, lot 231016009) were applied first, followed by incubation with a rabbit primary against NRF2 (1:100; Abcam, cat. no. AB31163, RRID: AB_881705, lot 1030320-17) diluted in 10% normal horse serum and 0.3% Tween 20 (Thermo Fisher Scientific, cat. no. BP337100) in 0.1 M TBS overnight at room temperature and a 1.5-hour incubation in donkey anti-rabbit Alexa647-conjugated secondary antibody (1:200; Thermo Fisher Scientific, cat. no. A-31571, RRID: AB_162542, lot 3219281). For neuronal staining, mouse primary antibody against SMI-32 (1:500; BioLegend, cat. no. 801701, RRID: AB_2564642, lot B448462), horse anti-mouse HRP-ImmPress secondary antibody (Vector Laboratories, cat. no. VECTMP740215, lot ZL1001) and tyramide Opal-520 fluorophore (1:100; Akoya Biosciences, cat. no. FP1487001KT, lot 230919042) were applied, with all washes including 0.3% Tween 20. Sections were washed, and remaining tyramide signal amplification was heat stripped via hot citrate buffer. Slides were cooled, and then chicken primary antibody against neurofilament (1:500; BioLegend, cat. no. 822601) was incubated overnight at room temperature, followed by washing and 1.5-hour incubation with donkey anti-chicken Alexa647 secondary antibody (1:200; Thermo Fisher Scientific, cat. no. 78952, lot 2622383).

### Immunofluorescent imaging and quantification of human white matter

Tile-scanned images across human FFPE sections were generated by a Zeiss AxioScan.Z1 slide scanner with a ×20 Plan Apochromat objective (numerical aperture 0.8). For Olig2 density analysis, the entirety of each FFPE section imaged was analyzed, corresponding to approximately 1.5 × 1.5 cm^2^ of tissue surveyed and a minimum of 62,000 nuclei per individual. Olig2^+^ cell densities were quantified using the cell detection application in QuPath (version 0.5.1). Thresholds were chosen (between 1,200 and 3,400 for Hoechst; between 2,700 and 4,700 for Olig2), which optimized the cell detection algorithm without losing positive cells or introducing background signal. Fixed thresholds were not applied across samples as these resulted in false-positive and false-negative detections. Autofluorescent blood vessels were manually traced and removed. For NRF2 and SMI-32 analyses, at least 3 × 1 mm^2^ randomly sampled technical replicate images were exported and analyzed with Fiji/ImageJ (Fiji.sc version 2.1.0/1.53c). For NRF2 analysis, NRF2^+^Olig2^+^ cells were manually quantified with the Cell Counter plugin in ImageJ. For SMI-32 analysis, any SMI-32^+^ cell body staining co-localizing with Hoechst was circled using the oval tool in Fiji and manually excluded to specifically assess axonal SMI-32. Fixed threshold analyses unreliably yielded false-positive and false-negative threshold detection across samples, so the automatic threshold detector in ImageJ was used to detect SMI-32 signal. For all stains, the average of technical replicates was used as one biological replicate. Case cognitive scores, sex, age and IQ values were blinded to the investigator during the analysis. Results were exported from QuPath/ImageJ and graphed using R (version 4.4.2).

### *Nfe2l2* oligodendrocyte-specific conditional knockout mice

*Plp*-Cre^ERT^ mice^96^ on a C57BL/6J background (Popko laboratory, Northwestern University) were crossed with *Nfe2l2*^fl/fl^ mice (The Jackson Laboratory, 025433)^97^ on a C57BL/6J background to obtain a tamoxifen-inducible conditional knockout mouse (*Plp*-Cre^ERT^;*Nfe2l2*^fl/fl^). Animals (both sexes) were housed in pathogen-free, temperature-controlled conditions (maintained at an ambient temperature of 20−24 °C and 25−50% humidity) in accordance with the guidelines set forth by the Northwestern University Institutional Animal Care and Use Committee. Animals had ad libitum access to food and water and were on a 12-hour per day light on/off cycle. At 6 months, animals were intraperitoneally injected with 1 mg d^−1^ 4-hydroxy-tamoxifen (4-OHT; Sigma-Aldrich, H6278) diluted in sunflower oil for five consecutive days. At 12 months, animals underwent cognitive testing, first on the large open field (LOF) test and then the MWM. After cognitive testing, a subset of the 12-month-old animals were either intracardially perfused with 4% PFA (Thermo Fisher Scientific, cat. no. T353-500) and snap frozen in optimal cutting temperature (OCT) compound for immunofluorescence or intracardially perfused with 0.1 M cold PBS (Thermo Fisher Scientific, cat. no. BP399-20) followed by 4% PFA (Thermo Fisher Scientific, cat. no. T353-500) with 2.5% glutaraldehyde (Electron Microscopy Sciences, cat. no. 16220) in 0.1 M sodium cacodylate (Electron Microscopy Sciences, cat. no. 11652) for electron microscopy. Separate cohorts of *Plp*-Cre^ERT^;*Nfe2l2*^fl/fl^ animals (which did not receive behavioral testing) were collected at 15 months and 18.5 months for immunohistochemistry and electron microscopy, respectively. Mice from both sexes were used at each timepoint; however, female mice showed genotype-specific differences in the LOF test (Extended Data Fig. 4a,b) and, thus, were excluded from further analysis.

### RNAscope

RNAscope was performed using an RNAscope v2 Multiplex Fluorescence Kit (ACDBio, cat. no. 323136) with a probe against murine *Nfe2l2* (ACDBio, cat. no. 475571), following the ACDBio protocol. Coronal sections from 12−15-month-old *Plp*-Cre^ERT^;*Nfe2l2*^fl/fl^ and age-matched controls were thawed for 30−60 minutes at room temperature and then dehydrated by 5-minute incubations of increasing ethanol concentrations (50%, 70%, 95%, 100%). Hydrophobic barriers were drawn around tissue (ImmEdge barrier pen; ACDBio, cat. no. 323136), and sections were incubated in hydrogen peroxide for 10 minutes. Slides were placed in hot antigen retrieval buffer at 95 °C for 5 minutes and then cooled. Manual Pretreat Pro (RNAscope v2 Multiplex Fluorescence Kit; ACDBio, cat. no. 323136) was incubated at 40 °C for 30 minutes, and the *Nfe2l2* probe was hybridized at 40 °C for 2 hours. Separate sections were hybridized for 3-plex positive (Pol2RA; PPIPB; UBC) or negative (DapB) control probes. A series of amplification probes (AMP1,2,3) were applied in 15−30-minute incubations, after which secondary antibodies were added (Akoya Biosciences, FP1496001KT). HRP blocker was added for 15 minutes at 40 °C, and tissue was incubated overnight with primary antibody against Olig2 (goat anti-Olig2 at 1:200; R&D Systems, cat. no. AF2418). Sections were washed, and donkey anti-goat Alexa568 secondary antibody (1:200; Thermo Fisher Scientific, cat. no. 11057) and Hoechst (1:10,000) were applied for 2 hours at room temperature before being washed and mounted.

### Behavioral assays

Twelve-month-old (±10 days) male and female *Plp*-Cre^ERT^;*Nfe2l2*^fl/fl^ and age-matched control mice were tested at the Northwestern University Behavioral Phenotyping Core. Mice were moved to the behavioral testing facilities 30 minutes prior to testing for habituation. Between active testing, animals had ab libitum access to water and food. A maximum of 25 animals per day were tested. Animals underwent testing on the LOF (54.6-cm^2^ arena) in a soundproof room under white light, with four animals tested simultaneously in four separate closets. Arenas of the LOF were defined as 18-cm^2^ squares at the center or corners of the arena. Animals were allowed to explore freely for 10 minutes. LimeLight5 software from Actimetrics recorded time spent in the center or corners. LOF arenas were cleaned with 70% ethanol between each animal. Female *Plp*-Cre^ERT^;*Nfe2l2*^fl/fl^ animals showed significantly altered anxiety-like behavior and were, thus, excluded from further analysis. The MWM consisted of a circular water bath (diameter 1.5 m) filled with water and a non-toxic white dye that discolored the water, and was divided into four quadrants corresponding to ordinal directions (NE/SE/SW/NW) with visible cues (91.4 cm wide) on the wall at each direction of the maze (N/E/S/W). MWM testing took place over 9 days, consisting of a 3-day acclimatization phase, a 2-day rest period and then a 4-day testing phase. During acclimatization, a visible platform (20 × 3 × 25.4 cm) was submerged but identifiable via projecting pillars. On each day, animals were tested in two sessions, with four trials per session (total of eight trials per day per animal). In each trial, mice were allowed to navigate to the platform for up to 1 minute and removed upon successful navigation to the platform. If a mouse ceased swimming and floated on its back, the mouse was removed and the trial was ended. At the end of the trial, the platform changed location; the order of which quadrant the mouse was placed in, to where the visible platform was located, was NE to NW; SE to SW; SW to NE; and NE to SE. Next, during testing, a hidden platform (19.05 cm in diameter) was submerged without visible pillars and remained in the same location in the NE quadrant. On each day, animals were tested in two sessions, with three trials per session (total of six trials per day per animal), and were placed in different starting quadrants corresponding to the following order: SE, NW, SW. As above, in each trial, animals were allowed to navigate to the platform for up to 1 minute, removed upon successful navigation to the platform and removed/trial ended if an animal ceased swimming and floated on its back. If an animal failed to reach the platform after 1 minute (in either phase), they were placed on the platform for another minute. During all trials, investigators remained visibly hidden to the animals by stepping behind a curtain. WaterMaze6 software (Actimetrics) recorded mouse location, and experimenters measured time to reach the platform with a manual timer. The daily average of trials in either acclimatization or testing phases was averaged to compute the time spent in the target quadrant, average swimming speed (cm s^−1^) and time taken to reach the platform (latency). Total path traces for each trial in the test phase were manually categorized by an experimenter blinded to genotype into search strategies^53^: direct (6), directed search (5), focal search (4), indirect search (4), chaining (3), scanning (2), random search (1) and thigmotaxis (0). The percentage of trials adopting the various search strategies on each testing day, the composite score across trials per day and the fold change in composite score (relative to the day 1 within-genotype average) were calculated per animal. To correlate performance in the MWM with neuropathological features, animals were split into two groups based on the average fold change in the percentage of time spent in the target quadrant on day 4, with ‘better performers’ having an above-average fold change and ‘poorer performers’ having a below-average fold change.

### Immunofluorescent staining, imaging and analysis of murine white matter

Coronal sections (25 µm) from 15-month-old *Plp*-Cre^ERT^;*Nfe2l2*^fl/fl^ animals were thawed for 10 minutes and then blocked for 1 hour with 5% normal horse serum and 0.5% Triton-X (BioShop Canada, cat. no. TRX777.100) in 0.1 M PBS (Thermo Fisher Scientific, cat. no. BP399-20). Primary antibodies against Olig2 (1:100; Sigma-Aldrich, cat. no. AB9610, RRID: AB_570666), CC1 (1:200; Abcam, cat. no. AB16794, RRID: AB_443473) and PDGFRα (1:200; R&D Systems, cat. no. AF1062, RRID: AB_2236897) were incubated overnight at room temperature in a humid chamber. Tissue was washed in 0.1 M PBS three times for 5 minutes. Secondary antibodies donkey anti-goat Alexa488 (1:100; Thermo Fisher Scientific, cat. no. A-11055, RRID: AB_2534102), donkey anti-rabbit Alexa568 (1:100; Thermo Fisher Scientific, cat. no. A10042, RRID: AB_2534017) and donkey anti-mouse Alexa647 (1:100; Thermo Fisher Scientific cat. no. A-31571, RRID: AB_162542) were applied for 2 hours, alongside Hoechst (1:10,000; Thermo Fisher Scientific, cat. no.H1399). Slides were washed in 0.1 M PBS three times for 5 minutes and coverslipped with Fluoromount G (Thermo Fisher Scientific, cat. no. 00-4958-02; Thermo Fisher Scientific, cat. no. 12541033CA). For each animal, at least three 10-µm *z*-stack tiled images through the corpus callosum were taken on a Zeiss LSM 900 with a ×20 magnification lens (numerical aperture 0.8). Maximum projections were manually counted for density of oligodendrocyte lineage cells using ImageJ (version 2.1.0/1.53c), and the average of the three technical replicates was used as one biological replicate.

### Electron microscopic sample processing, imaging and analysis of murine white matter

Twelve-month-old and 18-month-old mice were perfused with 4% PFA (Thermo Fisher Scientific, cat. no. T353-500) and 2.5% glutaraldehyde (Electron Microscopy Sciences, cat. no. 16220) in a 0.1 M sodium cacodylate solution (Electron Microscopy Sciences, cat. no. 11652). Corpus callosa were dissected and post-fixed in the above fixative for 2 weeks at 4 °C and then embedded in epoxy resin (Epon; Electron Microscopy Sciences, cat. no. 14900). Resin blocks were cut to ultra-thins (approximately 80 nm) at the Cellular and Molecular Electron Microscopy (CMEM) facility at SickKids Research Institute/Lunenfeld-Tanenbaum Research Institute and stained with uranyl acetate and lead citrate, and grids were imaged on a Hitachi HT7800 transmission electron microscope. For each animal, at least 200 myelinated axons were traced for axon diameter, myelin thickness (*g* ratio) and inner tongue thickness using Fiji ImageJ and assuming circularity, as above.

### Statistical analysis

Statistical analyses were computed using R (version 4.4.2) or GraphPad Prism (version 10.2.3). Pearson’s correlation tests were performed for correlation analyses. For testing the difference between means of two groups, two-sided unpaired Student’s *t*-tests were performed when there was equal variance between groups, and Wilcoxon rank-sum tests were performed when variance was unequal. For analysis over two variables, two-way ANOVAs were used with Tukey’s post hoc multiple comparisons tests or Sidak’s/Fisher’s least significant difference (LSD) multiple comparisons correction. Grubbs testing on group means identified a statistically significant outlier to exclude from *g* ratio analysis at 12 months. Two animals were excluded from behavioral data analysis due to corrupted video files. For correlational graphs, the 95% confidence interval was calculated using the ggplot2 package (version 4.0.3) in R (version 4.4.2). As per General Data Protection Regulation (GDPR) requirements for the European Union, individual-level data correlating human neuropathological characteristics to identifiable demographic or clinical information could not be displayed; however, correlations among cohort-level clinical, technical and neuropathological variables were displayed via correlation matrices (Pearson’s correlations). For all correlational analyses, categorical variables of sex (male/female) and neuropathological evidence of small vessel disease (yes/no) were classed as 1 or 0, respectively, and severity of Alzheimer’s disease pathology was classified on a numeric scale of Braak pathology ranging from 0 to 6. Power calculations were performed using OpenEpi software (https://openepi.com) and reached greater than 80% power for all experiments.

### Reporting summary

Further information on research design is available in the Nature Portfolio Reporting Summary linked to this article.

## Online content

Any methods, additional references, Nature Portfolio reporting summaries, source data, extended data, supplementary information, acknowledgements, peer review information; details of author contributions and competing interests; and statements of data and code availability are available at https://doi.org/10.1038/s41591-026-04608-y.

## Supplementary information

Supplementary Information Table 1 and Legend.

Reporting Summary

Peer Review File

Supplementary Data 1

## Data Availability

Individual-level LBC1936 participant data are not publicly available because they contain potentially identifiable genomic information and are protected under participant consent agreements and the UK GDPR. Access to LBC1936 data may be requested through the Lothian Birth Cohorts at The University of Edinburgh under a data usage agreement, subject to approval by the Lothian Birth Cohorts Access Committee. Raw snRNA-seq data have been deposited in the European Genome-Phenome Archive (EGA) (study ID EGAS50000001978; dataset ID EGAD50000002862) and are available through the EGA after approval of a data access request via the Lothian Birth Cohorts Access Committee. Aggregated cohort-level summary data derived from the human samples, together with all data generated from the mouse experiments, are available from the corresponding authors upon reasonable request.

## Code availability

Cognitive modeling is publicly available at https://edwebcontent.ed.ac.uk/sites/default/files/atoms/files/r_factor_of_curves_g_txt.txt. Sequencing analysis pipelines are not publicly available because they contain potentially identifiable genomic information and are protected under participant consent agreements and the UK GDPR and are available upon request to the corresponding authors after approval of a data usage agreement with the Lothian Birth Cohort at The University of Edinburgh, subject to approval by the Lothian Birth Cohorts Access Committee.

## References

[1] Scholz, J., Klein, M. C., Behrens, T. E. J. & Johansen-Berg, H. Training induces changes in white matter architecture. *Nat. Neurosci.* **12**, 1370–1371 (2009). 10.1038/nn.2412 PMC2770457 19820707

[2] McKenzie, I. A. et al. Motor skill learning requires active central myelination. *Science* **346**, 318–322 (2014). 10.1126/science.1254960 PMC6324726 25324381

[3] Xiao, L. et al. Rapid production of new oligodendrocytes is required in the earliest stages of motor skill learning. *Nat. Neurosci.* **19**, 1210–1217 (2016). 10.1038/nn.4351 PMC5008443 27455109

[4] Pan, S., Mayoral, S. R., Choi, H. S., Chan, J. R. & Kheirbek, M. A. Preservation of a remote fear memory requires new myelin formation. *Nat. Neurosci.* **23**, 487–499 (2020). 10.1038/s41593-019-0582-1 PMC7213814 32042175

[5] Steadman, P. E. et al. Disruption of oligodendrogenesis impairs memory consolidation in adult mice. *Neuron* **105**, 150–164 (2020). 10.1016/j.neuron.2019.10.013 PMC7579726 31753579

[6] Wang, F. et al. Myelin degeneration and diminished myelin renewal contribute to age-related deficits in memory. *Nat. Neurosci.* **23**, 481–486 (2020). 10.1038/s41593-020-0588-8 PMC7306053 32042174

[7] Phillips, K. A. et al. Age-related changes in myelin of axons of the corpus callosum and cognitive decline in common marmosets. *Am. J. Primatol.* **81**, e22949 (2019). 10.1002/ajp.22949 PMC6685070 30620098

[8] Lu, P. H. et al. Age-related slowing in cognitive processing speed is associated with myelin integrity in a very healthy elderly sample. *J. Clin. Exp. Neuropsychol.* **33**, 1059–1068 (2011). 10.1080/13803395.2011.595397 PMC3269444 22133139

[9] Lu, P. H. et al. Myelin breakdown mediates age-related slowing in cognitive processing speed in healthy elderly men. *Brain Cogn.* **81**, 131–138 (2013). 10.1016/j.bandc.2012.09.006 23195704

[10] Gong, Z. et al. Lower myelin content is associated with more rapid cognitive decline among cognitively unimpaired individuals. *Alzheimers Dement.* **19**, 3098–3107 (2023). 10.1002/alz.12968 PMC10387505 36720000

[11] Cox, S. R., Ritchie, S. J., Fawns-Ritchie, C., Tucker-Drob, E. M. & Deary, I. J. Structural brain imaging correlates of general intelligence in UK Biobank. *Intelligence* **76**, 101376 (2019). 10.1016/j.intell.2019.101376 PMC6876667 31787788

[12] Ritchie, S. J. et al. Coupled changes in brain white matter microstructure and fluid intelligence in later life. *J. Neurosci.* **35**, 8672–8682 (2015). 10.1523/JNEUROSCI.0862-15.2015 PMC4452562 26041932

[13] Taylor, A. M., Pattie, A. & Deary, I. J. Cohort profile update: the Lothian Birth Cohorts of 1921 and 1936. *Int. J. Epidemiol.* **47**, 1042–1042r (2018). 10.1093/ije/dyy022 PMC6124629 29546429

[14] Ritchie, S. J. et al. Predictors of ageing-related decline across multiple cognitive functions. *Intelligence* **59**, 115–126 (2016). 10.1016/j.intell.2016.08.007 PMC5127886 27932854

[15] Conte, F. P. et al. Cognitive change before old age (11 to 70) predicts cognitive change during old age (70 to 82). *Psychol. Sci.* **33**, 1803–1817 (2022). 10.1177/09567976221100264 PMC9660354 36113037

[16] Corley, J. et al. Predictors of longitudinal cognitive ageing from age 70 to 82 including *APOE* e4 status, early-life and lifestyle factors: the Lothian Birth Cohort 1936. *Mol. Psychiatry* **28**, 1256–1271 (2023). 10.1038/s41380-022-01900-4 PMC10005946 36481934

[17] Hinkley, L. B. N. et al. The role of corpus callosum development in functional connectivity and cognitive processing. *PLoS ONE* **7**, e39804 (2012). 10.1371/journal.pone.0039804 PMC3411722 22870191

[18] Mesaros, S. et al. Corpus callosum damage and cognitive dysfunction in benign MS. *Hum. Brain Mapp.* **30**, 2656–2666 (2009). 10.1002/hbm.20692 PMC6870794 19067325

[19] Huang, X. et al. Cognitive impairments associated with corpus callosum infarction: a ten cases study. *Int. J. Clin. Exp. Med.* **8**, 21991−21998 (2015). PMC4724017 26885171

[20] Ota, M. et al. Age-related degeneration of corpus callosum measured with diffusion tensor imaging. *Neuroimage* **31**, 1445–1452 (2006). 10.1016/j.neuroimage.2006.02.008 16563802

[21] Biegon, A. et al. Human corpus callosum in aging and Alzheimer’s disease: a magnetic resonance imaging study. *Neurobiol. Aging* **15**, 393–397 (1994). 10.1016/0197-4580(94)90070-1 7969715

[22] Craig, G. A. et al. Reflective imaging of myelin integrity in the human and mouse central nervous systems. *Front. Cell. Neurosci.* **18**, 1408182 (2024). 10.3389/fncel.2024.1408182 PMC11266064 39049821

[23] Jang, S. Y. et al. Autophagic myelin destruction by Schwann cells during Wallerian degeneration and segmental demyelination. *Glia* **64**, 730–742 (2016). 10.1002/glia.22957 26712109

[24] Schäffner, E. et al. Myelin insulation as a risk factor for axonal degeneration in autoimmune demyelinating disease. *Nat. Neurosci.* **26**, 1218–1228 (2023). 10.1038/s41593-023-01366-9 PMC10322724 37386131

[25] Yong, Y., Hunter-Chang, S., Stepanova, E. & Deppmann, C. Axonal spheroids in neurodegeneration. *Mol. Cell. Neurosci.* **117**, 103679 (2021). 10.1016/j.mcn.2021.103679 PMC8742877 34678457

[26] Webster, H. Transient, focal accumulation of axonal mitochondria during the early stages of Wallerian degeneration. *J. Cell Biol.* **12**, 361–383 (1962). 10.1083/jcb.12.2.361 PMC2106025 14005486

[27] Barrientos, S. A. et al. Axonal degeneration is mediated by the mitochondrial permeability transition pore. *J. Neurosci.* **31**, 966–978 (2011). 10.1523/JNEUROSCI.4065-10.2011 PMC3245862 21248121

[28] Stahon, K. E. et al. Age-related changes in axonal and mitochondrial ultrastructure and function in white matter. *J. Neurosci.* **36**, 9990–10001 (2016). 10.1523/JNEUROSCI.1316-16.2016 PMC5039264 27683897

[29] Schultz, V. et al. Acutely damaged axons are remyelinated in multiple sclerosis and experimental models of demyelination. *Glia* **65**, 1350–1360 (2017). 10.1002/glia.23167 PMC5518437 28560740

[30] Seeker, L. A. et al. Brain matters: unveiling the distinct contributions of region, age, and sex to glia diversity and CNS function. *Acta Neuropathol. Commun.* **11**, 84 (2023). 10.1186/s40478-023-01568-z PMC10204264 37217978

[31] Jäkel, S. et al. Altered human oligodendrocyte heterogeneity in multiple sclerosis. *Nature* **566**, 543–547 (2019). 10.1038/s41586-019-0903-2 PMC6544546 30747918

[32] Tran, M. N. et al. Single-nucleus transcriptome analysis reveals cell-type-specific molecular signatures across reward circuitry in the human brain. *Neuron* **109**, 3088–3103 (2021). 10.1016/j.neuron.2021.09.001 PMC8564763 34582785

[33] Pandey, S. et al. Disease-associated oligodendrocyte responses across neurodegenerative diseases. *Cell Rep.* **40**, 111189 (2022). 10.1016/j.celrep.2022.111189 36001972

[34] Falcão, A. M. et al. Disease-specific oligodendrocyte lineage cells arise in multiple sclerosis. *Nat. Med.* **24**, 1837–1844 (2019). 10.1038/s41591-018-0236-y PMC6544508 30420755

[35] Kenigsbuch, M. et al. A shared disease-associated oligodendrocyte signature among multiple CNS pathologies. *Nat. Neurosci.* **25**, 876–886 (2022). 10.1038/s41593-022-01104-7 PMC9724210 35760863

[36] Park, H. et al. Single-cell RNA-sequencing identifies disease-associated oligodendrocytes in male APP NL-GF and 5XFAD mice. *Nat. Commun.* **14**, 802 (2023). 10.1038/s41467-023-36519-8 PMC9925742 36781874

[37] McNamara, N. B. et al. Microglia regulate central nervous system myelin growth and integrity. *Nature* **613**, 120–129 (2023). 10.1038/s41586-022-05534-y PMC9812791 36517604

[38] Sadick, J. S. et al. Astrocytes and oligodendrocytes undergo subtype-specific transcriptional changes in Alzheimer’s disease. *Neuron* **110**, 1788–1805 (2022). 10.1016/j.neuron.2022.03.008 PMC9167747 35381189

[39] Barba-Reyes, J. M. et al. Oligodendroglia vulnerability in the human dorsal striatum in Parkinson’s disease. *Acta Neuropathol.* **149**, 46 (2025). 10.1007/s00401-025-02884-5 PMC12053221 40323467

[40] Niu, R.-Z., Feng, W.-Q., Chen, L. & Bao, T.-H. Single-cell transcriptomic profiling reveals regional differences in the prefrontal and entorhinal cortex of Alzheimer’s disease brain. *Int. J. Mol. Sci.* **26**, 4841 (2025). 10.3390/ijms26104841 PMC12112128 40429980

[41] Gergues, M. M., Moiseyenko, A., Saad, S. Z., Kong, A.-N. & Wagner, G. C. Nrf2 deletion results in impaired performance in memory tasks and hyperactivity in mature and aged mice. *Brain Res.* **1701**, 103–111 (2018). 10.1016/j.brainres.2018.08.033 PMC8111504 30194014

[42] Zweig, J. A., Caruso, M., Brandes, M. S. & Gray, N. E. Loss of NRF2 leads to impaired mitochondrial function, decreased synaptic density and exacerbated age-related cognitive deficits. *Exp. Gerontol.* **131**, 110767 (2020). 10.1016/j.exger.2019.110767 PMC6953739 31843395

[43] Zweig, J. A. et al. Loss of NRF2 accelerates cognitive decline, exacerbates mitochondrial dysfunction, and is required for the cognitive enhancing effects of *Centella asiatica* during aging. *Neurobiol. Aging* **100**, 48–58 (2021). 10.1016/j.neurobiolaging.2020.11.019 PMC7920997 33486357

[44] Bahn, G. et al. NRF2/ARE pathway negatively regulates BACE1 expression and ameliorates cognitive deficits in mouse Alzheimer’s models. *Proc. Natl Acad. Sci. USA* **116**, 12516–12523 (2019). 10.1073/pnas.1819541116 PMC6589670 31164420

[45] Branca, C. et al. Genetic reduction of Nrf2 exacerbates cognitive deficits in a mouse model of Alzheimer’s disease. *Hum. Mol. Genet.* **26**, 4823–4835 (2017). 10.1093/hmg/ddx361 PMC5886066 29036636

[46] Ren, P. et al. Nrf2 ablation promotes Alzheimer’s disease-like pathology in APP/PS1 transgenic mice: the role of neuroinflammation and oxidative stress. *Oxid. Med. Cell. Longev.* **2020**, 3050971 (2020). 10.1155/2020/3050971 PMC7238335 32454936

[47] Rojo, A. I. et al. Deficiency in the transcription factor NRF2 worsens inflammatory parameters in a mouse model with combined tauopathy and amyloidopathy. *Redox Biol.* **18**, 173–180 (2018). 10.1016/j.redox.2018.07.006 PMC6052199 30029164

[48] Sigfridsson, E., Marangoni, M., Hardingham, G. E., Horsburgh, K. & Fowler, J. H. Deficiency of Nrf2 exacerbates white matter damage and microglia/macrophage levels in a mouse model of vascular cognitive impairment. *J. Neuroinflammation* **17**, 367 (2020). 10.1186/s12974-020-02038-2 PMC7709339 33261626

[49] Liu, L. et al. Nrf2 deficiency exacerbates cognitive impairment and reactive microgliosis in a lipopolysaccharide-induced neuroinflammatory mouse model. *Cell. Mol. Neurobiol.* **40**, 1185–1197 (2020). 10.1007/s10571-020-00807-4 PMC11448839 32170572

[50] Sun, L. O. et al. Spatiotemporal control of CNS myelination by oligodendrocyte programmed cell death through the TFEB-PUMA axis. *Cell* **175**, 1811–1826 (2018). 10.1016/j.cell.2018.10.044 PMC6295215 30503207

[51] Young, K. M. et al. Oligodendrocyte dynamics in the healthy adult CNS: evidence for myelin remodeling. *Neuron* **77**, 873–885 (2013). 10.1016/j.neuron.2013.01.006 PMC3842597 23473318

[52] Tripathi, R. B. et al. Remarkable stability of myelinating oligodendrocytes in mice. *Cell Rep.* **21**, 316–323 (2017). 10.1016/j.celrep.2017.09.050 PMC5643547 29020619

[53] Sánchez, C. Q. et al. Search strategy analysis of 5xFAD Alzheimer mice in the Morris water maze reveals sex-and age-specific spatial navigation deficits. *Biomedicines* **11**, 599 (2023). 10.3390/biomedicines11020599 PMC9953202 36831135

[54] Drakesmith, M. et al. Estimating axon conduction velocity in vivo from microstructural MRI. *Neuroimage* **203**, 116186 (2019). 10.1016/j.neuroimage.2019.116186 PMC6854468 31542512

[55] Sampaio-Baptista, C. et al. Motor skill learning induces changes in white matter microstructure and myelination. *J. Neurosci.* **33**, 19499–19503 (2013). 10.1523/JNEUROSCI.3048-13.2013 PMC3858622 24336716

[56] Geraghty, A. C. et al. Loss of adaptive myelination contributes to methotrexate chemotherapy-related cognitive impairment. *Neuron* **103**, 250–265 (2019). 10.1016/j.neuron.2019.04.032 PMC6697075 31122677

[57] Fernández-Castañeda, A. et al. Mild respiratory COVID can cause multi-lineage neural cell and myelin dysregulation. *Cell* **185**, 2452–2468 (2022). 10.1016/j.cell.2022.06.008 PMC9189143 35768006

[58] van den Bosch, A. M. et al. Ultrastructural axon–myelin unit alterations in multiple sclerosis correlate with inflammation. *Ann. Neurol.* **93**, 856–870 (2023). 10.1002/ana.26585 36565265

[59] Peters, A. The effects of normal aging on myelinated nerve fibers in monkey central nervous system. *Front. Neuroanat.* **3**, 657 (2009). 10.3389/neuro.05.011.2009 PMC2713738 19636385

[60] Ferreira, S. et al. Oligodendrogenesis increases in hippocampal grey and white matter prior to locomotor or memory impairment in an adult mouse model of tauopathy. *Eur. J. Neurosci.* **54**, 5762–5784 (2021). 10.1111/ejn.14726 PMC8451881 32181929

[61] Wu, Y. et al. Alterations of myelin morphology and oligodendrocyte development in early stage of Alzheimer’s disease mouse model. *Neurosci. Lett.* **642**, 102–106 (2017). 10.1016/j.neulet.2017.02.007 28174059

[62] Hu, X. et al. Bace1 modulates myelination in the central and peripheral nervous system. *Nat. Neurosci.* **9**, 1520–1525 (2006). 10.1038/nn1797 17099708

[63] Groh, J. et al. Microglia-mediated demyelination protects against CD8^+^ T cell-driven axon degeneration in mice carrying PLP defects. *Nat. Commun.* **14**, 6911 (2023). 10.1038/s41467-023-42570-2 PMC10616105 37903797

[64] Marner, L., Nyengaard, J. R., Tang, Y. & Pakkenberg, B. Marked loss of myelinated nerve fibers in the human brain with age. *J. Comp. Neurol.* **462**, 144–152 (2003). 10.1002/cne.10714 12794739

[65] Hou, J. & Pakkenberg, B. Age-related degeneration of corpus callosum in the 90+ years measured with stereology. *Neurobiol. Aging* **33**, 1009.e1–9 (2012). 10.1016/j.neurobiolaging.2011.10.017 22118947

[66] Tang, Y., Nyengaard, J. R., Pakkenberg, B. & Gundersen, H. J. G. Age-induced white matter changes in the human brain: a stereological investigation. *Neurobiol. Aging* **18**, 609–615 (1997). 10.1016/s0197-4580(97)00155-3 9461058

[67] Aber, E. R. et al. Oligodendroglial macroautophagy is essential for myelin sheath turnover to prevent neurodegeneration and death. *Cell Rep.* **41**, 111480 (2022). 10.1016/j.celrep.2022.111480 PMC9639605 36261002

[68] Ktena, N. et al. Autophagic degradation of CNS myelin maintains axon integrity. *Cell Stress* **6**, 93–107 (2022). 10.15698/cst2022.12.274 PMC9707329 36478958

[69] Ktena, N. et al. Disruption of oligodendroglial autophagy leads to myelin morphological deficits, neuronal apoptosis, and cognitive decline in aged mice. *Glia* **73**, 1383−1397 (2025). 10.1002/glia.70012 PMC12121467 40105013

[70] Dhaliwal, N., Dhaliwal, J., Singh, A. & Chopra, K. Dimethyl fumarate attenuates 2-VO-induced vascular dementia via activating the Nrf2 signaling pathway in rats. *Inflammopharmacology* **29**, 537–547 (2021). 10.1007/s10787-020-00785-5 33459879

[71] Liu, Y. et al. Dimethylfumarate alleviates early brain injury and secondary cognitive deficits after experimental subarachnoid hemorrhage via activation of Keap1-Nrf2-ARE system. *J. Neurosurg.* **123**, 915–923 (2015). 10.3171/2014.11.JNS132348 25614941

[72] Wang, T. et al. Dimethyl fumarate improves cognitive impairment and neuroinflammation in mice with Alzheimer’s disease. *J. Neuroinflammation* **21**, 55 (2024). 10.1186/s12974-024-03046-2 PMC10882778 38383481

[73] Yan, N., Xu, Z., Qu, C. & Zhang, J. Dimethyl fumarate improves cognitive deficits in chronic cerebral hypoperfusion rats by alleviating inflammation, oxidative stress, and ferroptosis via NRF2/ARE/NF-κB signal pathway. *Int. Immunopharmacol.* **98**, 107844 (2021). 10.1016/j.intimp.2021.107844 34153667

[74] Amato, M. P. et al. Effects of 2-year treatment with dimethyl fumarate on cognition and functional impairment in patients with relapsing remitting multiple sclerosis. *Neurol. Sci.* **41**, 3185–3193 (2020). 10.1007/s10072-020-04320-w 32358701

[75] Yeung, M. S. Y. et al. Dynamics of oligodendrocyte generation and myelination in the human brain. *Cell* **159**, 766–774 (2014). 10.1016/j.cell.2014.10.011 25417154

[76] Chen, J.-F. et al. Enhancing myelin renewal reverses cognitive dysfunction in a murine model of Alzheimer’s disease. *Neuron* **109**, 2292–2307 (2021). 10.1016/j.neuron.2021.05.012 PMC8298291 34102111

[77] Rajani, R. M. et al. Selective suppression of oligodendrocyte-derived amyloid beta rescues neuronal dysfunction in Alzheimer’s disease. *PLoS Biol.* **22**, e3002727 (2024). 10.1371/journal.pbio.3002727 PMC11265669 39042667

[78] Holloway, R. K. et al. Localized microglia dysregulation impairs central nervous system myelination in development. *Acta Neuropathol. Commun.* **11**, 49 (2023). 10.1186/s40478-023-01543-8 PMC10035254 36949514

[79] Fan, W. et al. NRF2 activation ameliorates blood–brain barrier injury after cerebral ischemic stroke by regulating ferroptosis and inflammation. *Sci. Rep.* **14**, 5300 (2024). 10.1038/s41598-024-53836-0 PMC10912757 38438409

[80] van Horssen, J. et al. Nrf2 and DJ1 are consistently upregulated in inflammatory multiple sclerosis lesions. *Free Radic. Biol. Med.* **49**, 1283–1289 (2010). 10.1016/j.freeradbiomed.2010.07.013 20673799

[81] Rajani, R. M. et al. Reversal of endothelial dysfunction reduces white matter vulnerability in cerebral small vessel disease in rats. *Sci. Transl. Med.* **10**, eaam9507 (2018). 10.1126/scitranslmed.aam9507 29973407

[82] Han, B. et al. Integrating spatial and single-cell transcriptomics to characterize the molecular and cellular architecture of the ischemic mouse brain. *Sci. Transl. Med.* **16**, eadg1323 (2024). 10.1126/scitranslmed.adg1323 38324639

[83] Gibson, E. M. et al. Neuronal activity promotes oligodendrogenesis and adaptive myelination in the mammalian brain. *Science* **344**, 1252304 (2014). 10.1126/science.1252304 PMC4096908 24727982

[84] Bacmeister, C. M. et al. Motor learning drives dynamic patterns of intermittent myelination on learning-activated axons. *Nat. Neurosci.* **25**, 1300–1313 (2022). 10.1038/s41593-022-01169-4 PMC9651929 36180791

[85] Zullo, J. M. et al. Regulation of lifespan by neural excitation and REST. *Nature* **574**, 359–364 (2019). 10.1038/s41586-019-1647-8 PMC6893853 31619788

[86] Knowles, J. K., Batra, A., Xu, H. & Monje, M. Adaptive and maladaptive myelination in health and disease. *Nat. Rev. Neurol.* **18**, 735–746 (2022). 10.1038/s41582-022-00737-3 36376595

[87] Gutchess, A. H. et al. Aging and the neural correlates of successful picture encoding: frontal activations compensate for decreased medial-temporal activity. *J. Cogn. Neurosci.* **17**, 84–96 (2005). 10.1162/0898929052880048 15701241

[88] Davis, S. W., Dennis, N. A., Daselaar, S. M., Fleck, M. S. & Cabeza, R. Que PASA? The posterior–anterior shift in aging. *Cereb. Cortex* **18**, 1201–1209 (2008). 10.1093/cercor/bhm155 PMC2760260 17925295

[89] Angel, L., Fay, S., Bouazzaoui, B. & Isingrini, M. Two hemispheres for better memory in old age: role of executive functioning. *J. Cogn. Neurosci.* **23**, 3767–3777 (2011). 10.1162/jocn_a_00104 21812559

[90] Deary, I. J., Pattie, A. & Starr, J. M. The stability of intelligence from age 11 to age 90 years: the Lothian Birth Cohort of 1921. *Psychol. Sci.* **24**, 2361–2368 (2013). 10.1177/0956797613486487 24084038

[91] Henstridge, C. M. et al. Post-mortem brain analyses of the Lothian Birth Cohort 1936: extending lifetime cognitive and brain phenotyping to the level of the synapse. *Acta Neuropathol. Commun.* **3**, 53 (2015). 10.1186/s40478-015-0232-0 PMC4559320 26335101

[92] Kay, K. R. et al. Studying synapses in human brain with array tomography and electron microscopy. *Nat. Protoc.* **8**, 1366–1380 (2013). 10.1038/nprot.2013.078 PMC3712649 23787894

[93] Liewald, D., Miller, R., Logothetis, N., Wagner, H.-J. & Schüz, A. Distribution of axon diameters in cortical white matter: an electron-microscopic study on three human brains and a macaque. *Biol. Cybern.* **108**, 541–557 (2014). 10.1007/s00422-014-0626-2 PMC4228120 25142940

[94] Osorio, D. & Cai, J. J. Systematic determination of the mitochondrial proportion in human and mice tissues for single-cell RNA-sequencing data quality control. *Bioinformatics* **37**, 963–967 (2021). 10.1093/bioinformatics/btaa751 PMC8599307 32840568

[95] Marques, S. et al. Oligodendrocyte heterogeneity in the mouse juvenile and adult central nervous system. *Science* **352**, 1326–1329 (2016). 10.1126/science.aaf6463 PMC5221728 27284195

[96] Doerflinger, N. H., Macklin, W. B. & Popko, B. Inducible site-specific recombination in myelinating cells. *Genesis* **35**, 63–72 (2003). 10.1002/gene.10154 12481300

[97] Reddy, N. M., Potteti, H. R., Mariani, T. J., Biswal, S. & Reddy, S. P. Conditional deletion of *Nrf2* in airway epithelium exacerbates acute lung injury and impairs the resolution of inflammation. *Am. J. Respir. Cell Mol. Biol.* **45**, 1161–1168 (2011). 10.1165/rcmb.2011-0144OC PMC3262666 21659655

