# Supplementary material for: Oligodendrocyte dysfunction in human age-related cognitive decline
Source: Nat Med. 2026 Aug 25;32(10):3661–71. doi: 10.1038/s41591-026-04608-y (PMC13668666; doi:10.1038/s41591-026-04608-y)
Supplement: Supplementary file 1 [file 41591_2026_4608_MOESM1_ESM.pdf]

nature medicine

Supplementary information https://doi.org/10.1038/s41591-026-04608-y

Oligodendrocyte dysfunction in human age-related cognitive decline

In the format provided by the authors and unedited

Supplementary Table 1. Lothian Birth Cohort Demographic data
Sex, age at death, cognition over aging, cause of death of cases, and use of tissue in this study, with range ± standard deviation.

Sample Type	N	Age	Sex	Cognition at age 70	Cognition at death	Slope of cognitive decline	Cause of death
Electron microscopy	15	78-85 (81 ± 2.2)	9M, 6F	15-22 (18 ± 1.8)	10-20 (14 ± 2.4)	-0.19- -0.48 (-0.28 ± 0.089)	Cancer (n=8), Pneumonia (n=2), Stroke (n=1), Infection (n=1), Kidney failure (n=1), Physical frailty/unknown (n=2)
Histology	21	78-85 (81 ± 2.2)	12M, 9F	14-22 (18 ± 2.1)	7-20 (14 ± 3.0)	-0.19- -0.65 (-0.31 ± 0.12)	Cancer (n=11), Pneumonia (n=4), Stroke (n=1), Infection (n=1), Kidney failure (n=1), Physical frailty/unknown (n=3)
RNA Sequencing	14	79-84 (82 ± 1.8)	8M, 6F	14 -22 (18 ± 2.4)	10-20 (14 ± 3.0)	-0.19- -0.48 (-0.28 ± 0.088)	Cancer (n=9), Pneumonia (n=3), Kidney failure (n=1), Physical frailty/unknown (n=1)
