# Supplementary material for: Oligodendrocyte dysfunction in human age-related cognitive decline
Source: Nat Med. 2026 Aug 25;32(10):3661–71. doi: 10.1038/s41591-026-04608-y (PMC13668666; doi:10.1038/s41591-026-04608-y)
Supplement: Supplementary file 2 [file 41591_2026_4608_MOESM2_ESM.pdf]

nature portfolio

Corresponding author(s): Georgina Craig
Veronique Miron

Last updated by author(s): 20 July 2026

Reporting Summary

Nature Portfolio wishes to improve the reproducibility of the work that we publish. This form provides structure for consistency and transparency in reporting. For further information on Nature Portfolio policies, see our Editorial Policies and the Editorial Policy Checklist.

Statistics

For all statistical analyses, confirm that the following items are present in the figure legend, table legend, main text, or Methods section.

n/a	Confirmed	
☐	☒	The exact sample size (*n*) for each experimental group/condition, given as a discrete number and unit of measurement
☐	☒	A statement on whether measurements were taken from distinct samples or whether the same sample was measured repeatedly
☐	☒	The statistical test(s) used AND whether they are one- or two-sided<br>*Only common tests should be described solely by name; describe more complex techniques in the Methods section.*
☐	☒	A description of all covariates tested
☐	☒	A description of any assumptions or corrections, such as tests of normality and adjustment for multiple comparisons
☐	☒	A full description of the statistical parameters including central tendency (e.g. means) or other basic estimates (e.g. regression coefficient) AND variation (e.g. standard deviation) or associated estimates of uncertainty (e.g. confidence intervals)
☐	☒	For null hypothesis testing, the test statistic (e.g. *F*, *t*, *r*) with confidence intervals, effect sizes, degrees of freedom and *P* value noted<br>*Give P values as exact values whenever suitable.*
☒	☐	For Bayesian analysis, information on the choice of priors and Markov chain Monte Carlo settings
☐	☒	For hierarchical and complex designs, identification of the appropriate level for tests and full reporting of outcomes
☐	☒	Estimates of effect sizes (e.g. Cohen's *d*, Pearson's *r*), indicating how they were calculated

Our web collection on statistics for biologists contains articles on many of the points above.

Software and code

Policy information about availability of computer code

Data collection

Human cognitive data was collected via cognitive tests (including the Moray House Test No. 12, the Weschler Memory Scale, and the Weschler Adult Intelligence Scale test), administered by trained psychologists. Reflective images were captured with a Leica Stellaris 5 confocal microscope using an RT85/15 beamsplitter. Electron micrographs were captured with a JEOL JEM 1400 Plus (human samples) or a Hitachi HT7800 (murine samples). Fluorescence images were captured with a Zeiss AxioScan.Z1 slide-scanner (human samples) or with a Zeiss LSM900 confocal microscope (murine samples). Single nuclei were sorted on a BD FACS Aria II cytometer and processed on the 10X Chromium single cell platform. cDNA libraries were assessed with an Agilent Tape Station HS D5000, an Agilent BioAnalyzer (#G2939AA), and a Qubit 2.0 Fluorometer (#Q32866). Human RNA sequences were read on an Illumina NextSeq2000, and basecall data (generated by NextSeq 1000/2000 Control Software v1.5.0.42699) was transferred to a computer running Ubuntu v20.04.2 LTS. Murine Olig2cre-Tfeb sequencing data was accessed from available data listed in Sun et al., 2018. In-situ hybridisation was performed using an ACD HyBEZ-II-Hybridization System. Behavioural data was collected using LimeLight5 software (Actimetrics; for Large Open Field) and WaterMaze6 software (Actimetrics; for Morris Water Maze).

Data analysis

Cognitive trajectory analyses: R (v4.4.2) with RStudio was used to run the code, and the lavaan (v0.6.21), psych (v2.6.5) and haven (v2.5.5) packages were used for modelling generalised cognition over time.
Imaging analyses: Fluorescence images were analysed using QuPath software (v0.5.1) and employing the cell detection and cell classification modules (human tissue), or using Fiji/ImageJ software (Fiji.sc v2.1.0/1.53c) and the cell counter plugin (murine tissue). Electron micrographs were analysed using Fiji/ImageJ (v2.1.0/1.53c) software’s polygon tracing tool. Reflection images were analysed using Fiji/ImageJ (v2.1.0/1.53c) software’s auto-threshold function.
Sequencing analyses: CellRanger (v7.1.0) software was used to generate fastq files via demultiplexing raw base call (BCL) files, and to align these to the human reference genome (GRCh38) supplied by 10X Genomics (Human 3.0.0). R (v4.4.2) was used to run the code, and the packages seuratObject (v5.1.0), Seurat (v5.2.1), limma (v3.56.2), and edgeR (v3.42.4) used for object handling, differential gene expression

analyses, and linear mixed modelling.
Pathway analysis: QIAGEN Ingenuity Pathway Analysis (IPA) software was used to identify predicted upstream regulators of DEGs.
Behavioral analyses: Behavioural data was assessed using LimeLight5 and WaterMaze6 for video tracking.
Data handling: GraphPad Prism (v10.2.3) and/or R (v4.4.2) with the tidyverse (v2.0.0), ggplot2 (v4.0.3), ggridges (v0.5.7), outliers (v0.15) and scales (v1.4.0) packages were used for data plotting and running statistical tests.
Sample Size Calculations: OpenEpi software (OpenEpi.com) was used to perform calculations of sample sizes required to be powered at >80% to detect significance.

For manuscripts utilizing custom algorithms or software that are central to the research but not yet described in published literature, software must be made available to editors and reviewers. We strongly encourage code deposition in a community repository (e.g. GitHub). See the Nature Portfolio guidelines for submitting code & software for further information.

Data

Policy information about availability of data

All manuscripts must include a data availability statement. This statement should provide the following information, where applicable:

- Accession codes, unique identifiers, or web links for publicly available datasets
- A description of any restrictions on data availability
- For clinical datasets or third party data, please ensure that the statement adheres to our policy

Data availability: statement
Individual-level Lothian Birth Cohort 1936 participant data are not publicly available because they contain potentially identifiable genomic information and are protected under participant consent agreements and UK GDPR. Access to Lothian Birth Cohort 1936 data may be requested through the Lothian Birth Cohorts at the University of Edinburgh under a Data Usage Agreement (DUA), subject to approval by the Lothian Birth Cohorts Access Committee. Raw single-nucleus RNA sequencing (snRNA-seq) data have been deposited in the European Genome-Phenome Archive (EGA) (study ID EGAS50000001978; dataset ID EGAD50000002862) and are available through the EGA following approval of a data access request via the Lothian Birth Cohorts Access Committee. Aggregated cohort-level summary data derived from the human samples, together with all data generated from the mouse experiments, are available from the corresponding authors upon reasonable request.

Code availability statement:
Cognitive modellng is publicly available at https://edwebcontent.ed.ac.uk/sites/default/files/atoms/files/r_factor_of_curves_g_txt.txt.
Sequencing analysis pipelines are not publicly available because they contain potentially identifiable genomic information and are protected under participant consent agreements and UK GDPR, and are available upon request to the corresponding authors following approval of a Data Usage Agreement (DUA) with the Lothian Birth Cohort at The University of Edinburgh, subject to approval by the Lothian Birth Cohorts Access Committee.

Research involving human participants, their data, or biological material

Policy information about studies with human participants or human data. See also policy information about sex, gender (identity/presentation), and sexual orientation and race, ethnicity and racism.

Reporting on sex and gender	This study used post-mortem tissue of human brain samples from deceased participants of the Lothian Birth Cohort 1936, and non-neurologically-impaired young adult controls from the Sudden Death Brain Bank at The University of Edinburgh. In the case of the Lothian Birth cohort, sex and gender data were collected at the time of recruitment, and data pertaining to sex was utilized in this manuscript; gender data as a variable was not requested for the purposes of this study. For adult Sudden Death post-mortem human samples, sex was included in clinical information and gender information was not included. Samples were prepared as resin-embedded blocks for electron microscopy, paraffin-embedded tissue for histology, and/or fresh-frozen blocks for single nuclei RNA sequencing, however not always for all 3. For each sample type, both sexes were included: for electron microscopic analysis, 9 males and 6 females were analyzed, with an age range of 75-85 years; for histological analyses, 12 males and 9 females were used, with an age range of 75-85 years; for sequencing, 8 males and 6 females were used, with an age range of 79-84 years. All information pertaining to these tissues is listed in Supplementary Data Table 1.
Reporting on race, ethnicity, or other socially relevant groupings	Human clinical data and biological samples utilized in this study derived from the Lothian Birth Cohort 1936, a Scotland-based cohort of majority white European descent, and from the Sudden Death Brain Bank in Edinburgh. This study did not analyze the impact of race, ethnicity, nor any other socially relevant groupings on data outcomes.
Population characteristics	Of the Lothian Birth Cohort 1936 biological samples utilized, 11 originated from individuals who had died from various cancers, 4 had a listed primary cause of death as pneumonia, 1 had a listed cause of death as kidney failure, 1 individual had sepsis secondary to achromobacter xylosoxidans infection, 1 individual had a primary cause of death as ischaemic stroke, and 3 had a primary cause of death as old age, physical frailty, or unknown. All information pertaining to these cause of deaths per sample type is listed in Supplementary Table 1. Other population characteristics of the living Lothian Birth Cohort can be found online at https://lothian-birth-cohorts.ed.ac.uk/. For the n=4 human post-mortem brain samples from the Sudden Death Brain Bank, all were male, ages ranging from 34-49 years, and causes of death were ischaemic heart disease and coronary artery atherosclerosis (2 individuals), myocardial infarction and coronary artery artherosclerosis (1 individual) and coronary artery atheroma and cardiomegaly (1 individual).
Recruitment	Individuals born in 1936 in Scotland were required in 1947 by the Scottish Council for Research in Education to undergo a 45-minute, group-administered, general intelligence quiz [the Moray House Test (MHT), No. 12] at mean age 11. In 2006, at age 70, 1,091 of these individuals consented and were recruited to participate in the Lothian Birth Cohort Studies. Ethics permission for the Lothian Birth Cohort 1936 was obtained from the Multi-Centre Research Ethics Committee for Scotland (Wave 1: MREC/01/0/56), the Lothian Research Ethics Committee (Wave 1: LREC/2003/2/29), and the Scotland A Research

	Ethics Committee (Waves 2-7: 07/MRE00/58). Consenting individuals provided written pre-mortem authorization for brain tissue donation following death. The use of post-mortem human tissue samples derived from The Lothian Birth Cohort 1936 was ethically approved by the Medical Research Council Brain Bank at The University of Edinburgh.
Ethics oversight	Cognitive data and post-mortem human tissue samples were obtained from The Lothian Birth Cohort 1936, and from the Medical Research Council Brain Bank at The University of Edinburgh. Ethics permission for the Lothian Birth Cohort 1936 was obtained from the Multi-Centre Research Ethics Committee for Scotland (Wave 1: MREC/01/0/56), the Lothian Research Ethics Committee (Wave 1: LREC/2003/2/29), and the Scotland A Research Ethics Committee (Waves 2-7: 07/MRE00/58).

Note that full information on the approval of the study protocol must also be provided in the manuscript.

Field-specific reporting

Please select the one below that is the best fit for your research. If you are not sure, read the appropriate sections before making your selection.

☒ Life sciences ☐ Behavioural & social sciences ☐ Ecological, evolutionary & environmental sciences

For a reference copy of the document with all sections, see nature.com/documents/nr-reporting-summary-flat.pdf

Life sciences study design

All studies must disclose on these points even when the disclosure is negative.

Sample size	Human: For human cognitive data, n=816-1,017 individuals were used (number varies due to drop-out over successive waves; all viable test results were included). For human biological samples, n=13-21 samples were used across analyses. We performed power calculations on our datasets by the normal approximation method using a two-sided confidence interval of 95% and noted that all statistical comparisons between mild vs severe cognitive decline groups were powered at over 80% (82-99%). This is consistent with previous studies utilizing the Lothian Birth Cohort tissue finding that n=13 is sufficient to detect differences between cognitive groupings at the histological level (King et al., 2023).<br><br>Mouse: For murine samples, OpenEpi.com was used to determine the sample size needed to reach >80% power at 0.05 significance. For histological analyses, n=5-6 mice per genotype per age were utilized. This exceeds the n=3 mice previously shown as sufficient to a) detect significant differences in myelin integrity associated with cognitive changes (McNamara et al., 2023) and b) detect differences in proteins in mice with myelin protein impairment (Depp et al., 2023). For behavioral analyses, n = 17-21 mice per sex per genotype were utilized. Sample sizes exceeded previous studies showing n=8-10 mice/group is sufficient to detect differences in behavior when myelin integrity is compromised (Depp et al., 2023).
Data exclusions	A small number of human samples had very poor tissue quality which impeded appropriate quantification and were thus excluded. Of 17 samples avaible for electron microscopic analyses, 2 had no evident intact myelinated axons when imaged and were excluded from all analysis, and 2 had severe myelin decompaction typical of fixation artefact (which would inflate myelin g ratio measurements) and were excluded from axon diameter and myelin thickness measurements. Of 21 paraffin embedded cases available, 3 cases were excluded based on extremely poor Hoechst signal. Of the 16 samples that underwent snRNAseq, 2 were removed after 1 round of sequencing due to poor library quality. At no point during the exclusion process were researchers unblinded to cognitive phenotype.<br><br>For animal histological data, 1 exclusion was applied to an animal who had a myelin thickness value that met the Grubb's criteria for being a statistical outlier. For behavioral experimentation, female transgenic animals showed significant anxiety on the large open field test compared to female non-transgenic littermates, thus all females were excluded from subsequent cognitive analyses. For the Morris Water maze behavioral tests, individual trials were excluded from the per-day average if there was a technical error during the trial which rendered the data meaningless (for example, being introduced to the incorrect quadrant).
Replication	Numbers of times each experiment were performed are as follows:<br>Cognitive data: n=816-1,017*5 testing waves<br>Single-nuclei RNA Sequencing: n = 16 individuals<br>Human fluorescence/reflectance imaging: n = 17-21 individuals*3-8 technical replicates<br>Human electron microscopy: n = 13-15 individuals*100 technical replicates<br>Murine electron microscopy: n = 9-14 individuals*200 technical replicates<br>Murine behavior: n=67 mice*6-8 technical replicates per day<br>Murine fluorescence staining and imaging: n=12 mice*3 technical replicates<br>Murine ISH: n=19 mice*3 technical replicates
Randomization	Mice were randomly allocated to assessment time points. Randomization of humans was not applicable, as all individuals and samples underwent the same experimental procedures.
Blinding	All researchers were blinded to cognitive phenotype of humans and genotype of mice.

Behavioural & social sciences study design

All studies must disclose on these points even when the disclosure is negative.

Study description	*Briefly describe the study type including whether data are quantitative, qualitative, or mixed-methods (e.g. qualitative cross-sectional, quantitative experimental, mixed-methods case study).*
Research sample	*State the research sample (e.g. Harvard university undergraduates, villagers in rural India) and provide relevant demographic information (e.g. age, sex) and indicate whether the sample is representative. Provide a rationale for the study sample chosen. For studies involving existing datasets, please describe the dataset and source.*
Sampling strategy	*Describe the sampling procedure (e.g. random, snowball, stratified, convenience). Describe the statistical methods that were used to predetermine sample size OR if no sample-size calculation was performed, describe how sample sizes were chosen and provide a rationale for why these sample sizes are sufficient. For qualitative data, please indicate whether data saturation was considered, and what criteria were used to decide that no further sampling was needed.*
Data collection	*Provide details about the data collection procedure, including the instruments or devices used to record the data (e.g. pen and paper, computer, eye tracker, video or audio equipment) whether anyone was present besides the participant(s) and the researcher, and whether the researcher was blind to experimental condition and/or the study hypothesis during data collection.*
Timing	*Indicate the start and stop dates of data collection. If there is a gap between collection periods, state the dates for each sample cohort.*
Data exclusions	*If no data were excluded from the analyses, state so OR if data were excluded, provide the exact number of exclusions and the rationale behind them, indicating whether exclusion criteria were pre-established.*
Non-participation	*State how many participants dropped out/declined participation and the reason(s) given OR provide response rate OR state that no participants dropped out/declined participation.*
Randomization	*If participants were not allocated into experimental groups, state so OR describe how participants were allocated to groups, and if allocation was not random, describe how covariates were controlled.*

Ecological, evolutionary & environmental sciences study design

All studies must disclose on these points even when the disclosure is negative.

Study description	*Briefly describe the study. For quantitative data include treatment factors and interactions, design structure (e.g. factorial, nested, hierarchical), nature and number of experimental units and replicates.*
Research sample	*Describe the research sample (e.g. a group of tagged Passer domesticus, all Stenocereus thurberi within Organ Pipe Cactus National Monument), and provide a rationale for the sample choice. When relevant, describe the organism taxa, source, sex, age range and any manipulations. State what population the sample is meant to represent when applicable. For studies involving existing datasets, describe the data and its source.*
Sampling strategy	*Note the sampling procedure. Describe the statistical methods that were used to predetermine sample size OR if no sample-size calculation was performed, describe how sample sizes were chosen and provide a rationale for why these sample sizes are sufficient.*
Data collection	*Describe the data collection procedure, including who recorded the data and how.*
Timing and spatial scale	*Indicate the start and stop dates of data collection, noting the frequency and periodicity of sampling and providing a rationale for these choices. If there is a gap between collection periods, state the dates for each sample cohort. Specify the spatial scale from which the data are taken*
Data exclusions	*If no data were excluded from the analyses, state so OR if data were excluded, describe the exclusions and the rationale behind them, indicating whether exclusion criteria were pre-established.*
Reproducibility	*Describe the measures taken to verify the reproducibility of experimental findings. For each experiment, note whether any attempts to repeat the experiment failed OR state that all attempts to repeat the experiment were successful.*
Randomization	*Describe how samples/organisms/participants were allocated into groups. If allocation was not random, describe how covariates were controlled. If this is not relevant to your study, explain why.*
Blinding	*Describe the extent of blinding used during data acquisition and analysis. If blinding was not possible, describe why OR explain why blinding was not relevant to your study.*

Did the study involve field work? ☐ Yes ☐ No

Field work, collection and transport

Field conditions: *Describe the study conditions for field work, providing relevant parameters (e.g. temperature, rainfall).*

Location: *State the location of the sampling or experiment, providing relevant parameters (e.g. latitude and longitude, elevation, water depth).*

Access & import/export: *Describe the efforts you have made to access habitats and to collect and import/export your samples in a responsible manner and in compliance with local, national and international laws, noting any permits that were obtained (give the name of the issuing authority, the date of issue, and any identifying information).*

Disturbance: *Describe any disturbance caused by the study and how it was minimized.*

Reporting for specific materials, systems and methods

We require information from authors about some types of materials, experimental systems and methods used in many studies. Here, indicate whether each material, system or method listed is relevant to your study. If you are not sure if a list item applies to your research, read the appropriate section before selecting a response.

Materials & experimental systems

n/a	Involved in the study
☐	☒ Antibodies
☒	☐ Eukaryotic cell lines
☒	☐ Palaeontology and archaeology
☐	☒ Animals and other organisms
☒	☐ Clinical data
☒	☐ Dual use research of concern
☒	☐ Plants

Methods

n/a	Involved in the study
☒	☐ ChIP-seq
☒	☐ Flow cytometry
☒	☐ MRI-based neuroimaging

Antibodies

Antibodies used:

Rabbit-anti-Olig2: Sigma-Aldrich; Cat#AB9610; lot 3857662; 1:100
Rabbit-anti-NRF2: Abcam; Cat#AB31163; lot 1030320-17; 1:100
Mouse-anti-Olig2: Sigma-Aldrich; Cat#MABN50; lot 421151; 1:100
Mouse-anti-APC[CC-1]: Abcam; Cat#AB16794; clone CC-1; lot 1075159-; 1:200
Goat-anti-PDGFRA: R&D Systems; Cat#AF1062; lot HMQ0223081; 1:200
Mouse-anti-SMI32: BioLegend; Cat#801701; lot B448462; 1:500
Chicken-anti-NFH: BioLegend; Cat#822601; lot B384954; 1:500
Horse-anti-Rabbit-HRP-ImmPress: Vector Laboratories; Cat#MP-7401; lot ZK0830; used neat
Horse-anti-Mouse-HRP-ImmPress: Vector Laboratories; Cat#MP-7402-15; lot ZL1001; used neat
Tyramide Opal-570: Akoya Biosciences; Cat#FP1488001KT; lot 231016009; 1:100
Tyramide Opal-520: Akoya Biosciences; Cat#FP1487001KT; lot 230919042; 1:100
Donkey-anti-Goat-Alexa-488: Thermo Fischer Scientific; Cat#A-11055; lot 3148294;1:100
Donkey-anti-Rabbit-Alexa-568: Thermo Fischer Scientific; Cat#A10042;lot 2941306; 1:100
Donkey-anti-Mouse-Alexa-647: Thermo Fischer Scientific; Cat#A-31571; lot 3141854; 1:100
Donkey-anti-Rabbit-Alexa-647: Thermo Fischer Scientific; Cat#A-31573; lot 3219281; 1:200
Donkey-anti-Chicken-Alexa-647: Thermo Fischer Scientific; Cat#78952; lot 2622383; 1:200
Hoechst: Thermo Fischer Scientific; Cat#H1399; lot 2936197; 1:1,000-1:10,000

Validation:

Rabbit-anti-Olig2 has been cited in 1,428 peer-reviewed publications, including to stain for oligodendrocyte-lineage cells in human epilepsy brain tissue (PMID40293058), human organoids (PMID39779941), and post-mortem human foetal cortical samples (PMID34390642). Rabbit-anti-NRF2 was validated by the supplier via knockdown and knockout strategies, and cited 510 times in studies to detect human-specific NRF2, including immunostaining of human post-mortem brain samples (PMID32051591). Mouse-anti-Olig2 has been cited 429 times in studies to detect Olig2, including in immunostaining of murine post-mortem brain tissue (PMID42334020; PMID42062598), primary murine OPC cultures in vitro (PMID42062598; PMID41825445), and in post-mortem human brain of Alzheimer’s Disease (PMID 40751749) or human infants (PMID 40023165). Mouse-anti-APC[CC-1] has been cited 204 times in studies to label mature oligodendrocytes (e.g. PMID41382275; PMID25031414) including our own work (PMID23872599; PMID40233100; PMID37291151; PMID36517604), and has been validated by the supplier for use in immunofluorescent staining. Goat-anti-PDGFRA has been cited 785 times in studies to label OPCs (e.g. PMID36280798; PMID29976621; PMID37926818) and has been tested and validated by the supplier for use in immunofluorescent staining. Mouse-anti-SMI32 has been cited in 262 studies, including to mark stressed axons in aged murine brain samples (PMID40404995; PMID37903797), human infant brain tissue (PMID40023165), human post-mortem adult cerebellar tissue (PMID40587795) and human post-mortem MS tissue (PMID41175293). Chicken-anti-NFH antibody has been verified by the supplier for use in immunofluorescence, and previously been used to label healthy axons in human iPSC-derived cells and organoids (PMID35350711; PMID33945785).

Eukaryotic cell lines

Policy information about cell lines and Sex and Gender in Research

Cell line source(s)	*State the source of each cell line used and the sex of all primary cell lines and cells derived from human participants or vertebrate models.*
Authentication	*Describe the authentication procedures for each cell line used OR declare that none of the cell lines used were authenticated.*
Mycoplasma contamination	*Confirm that all cell lines tested negative for mycoplasma contamination OR describe the results of the testing for mycoplasma contamination OR declare that the cell lines were not tested for mycoplasma contamination.*
Commonly misidentified lines (See ICLAC register)	*Name any commonly misidentified cell lines used in the study and provide a rationale for their use.*

Palaeontology and Archaeology

Specimen provenance	*Provide provenance information for specimens and describe permits that were obtained for the work (including the name of the issuing authority, the date of issue, and any identifying information). Permits should encompass collection and, where applicable, export.*
Specimen deposition	*Indicate where the specimens have been deposited to permit free access by other researchers.*
Dating methods	*If new dates are provided, describe how they were obtained (e.g. collection, storage, sample pretreatment and measurement), where they were obtained (i.e. lab name), the calibration program and the protocol for quality assurance OR state that no new dates are provided.*

☐ Tick this box to confirm that the raw and calibrated dates are available in the paper or in Supplementary Information.

Ethics oversight	*Identify the organization(s) that approved or provided guidance on the study protocol, OR state that no ethical approval or guidance was required and explain why not.*

Note that full information on the approval of the study protocol must also be provided in the manuscript.

Animals and other research organisms

Policy information about studies involving animals; ARRIVE guidelines recommended for reporting animal research, and Sex and Gender in Research

Laboratory animals	Plp-CreERT mice on the C57Bl/6J genetic background [Source: Popko laboratory at Northwestern University] were crossed with Nfe2l2fl/fl mice on a C57BL/6J genetic background [Source: the Jackson Laboratory (Stock number: 025433)] to obtain a tamoxifen-inducible conditional knockout mouse line (Plp-CreERT;Nfe2l2fl/fl) on a C57BL/6J genetic background. Plp-CreERT;Nfe2l2fl/fl animals (both sexes) were housed in pathogen-free, temperature-controlled conditions (maintained at an ambient temperature of 20-25C and 25-50% humidity) at Northwestern University, in accordance with the guidelines set forth by the Northwestern University Institutional Animal Care and Use Committee (IACUC). Animals had ad libitum access to food and water, and were on a 12hr/day light on/off cycle. At 6 months of age, animals were treated with 1 mg/day of a 4-hydroxy-tamoxifen (4-OHT) solution diluted in sunflower oil, via intraperitoneal injection for 5 consecutive days (in total, each mouse received 5 mg 4-OHT at the end of the 5 day procedure). Following recombination, animals were allowed to age. At 12 months of age, a cohort of 17-21 animals per sex per genotype (n=67 animals total) were assessed for cognitive functioning (first on the large open field test, then the morris water maze). Immediately following the end of behavioural testing, a subset (5-6 animals per sex per genotype) were collected for electron microscopy, while another subset (5-6 animals per sex per genotype) were collected for immunofluorescence. Separate cohorts of Plp-CreERT;Nfe2l2fl/fl animals (which did not receive behavioral testing) were collected at 15 months and 18.5 months for immunofluorescence and electron microscopy, respectively.<br>Animals collected for immunofluorescence were intracardially perfused with 4% paraformaldehyde, post-fixed in 4% paraformaldehyde overnight, then transferred into a sucrose (30%) solution prior to being snap-frozen in optimal cutting temperature and stored at -80C. Animals collected for electron microscopy were intracardially perfused, first with a flush of 0.1M cold PBS, then with a 4% paraformaldehyde and 2.5% glutaraldehyde solution in 0.1 M sodium cacodylate. Brains were dissected, corpus callosi sub-dissected, and tissue processed for electron microscopy.
Wild animals	No wild animals were used in this study.
Reporting on sex	Both sexes were used in this study in all mouse experiments, with the exception of the Morris Water Maze behavioural analysis, where only males were analysed after female transgenic animals triggered the exclusion criteria for confounding anxiety effects when tested in the large open field.
Field-collected samples	No field collected samples were used in this study.
Ethics oversight	Plp-CreERT;Nfe2l2fl/fl animals (both sexes) were housed in pathogen-free, temperature-controlled conditions at Northwestern University, in accordance with the guidelines set forth by the Northwestern University Institutional Animal Care and Use Committee

(IACUC).

Note that full information on the approval of the study protocol must also be provided in the manuscript.

Dual use research of concern

Policy information about dual use research of concern

Hazards

Could the accidental, deliberate or reckless misuse of agents or technologies generated in the work, or the application of information presented in the manuscript, pose a threat to:

No	Yes	
☒	☐	Public health
☒	☐	National security
☒	☐	Crops and/or livestock
☒	☐	Ecosystems
☒	☐	Any other significant area

Experiments of concern

Does the work involve any of these experiments of concern:

No	Yes	
☒	☐	Demonstrate how to render a vaccine ineffective
☒	☐	Confer resistance to therapeutically useful antibiotics or antiviral agents
☒	☐	Enhance the virulence of a pathogen or render a nonpathogen virulent
☒	☐	Increase transmissibility of a pathogen
☒	☐	Alter the host range of a pathogen
☒	☐	Enable evasion of diagnostic/detection modalities
☒	☐	Enable the weaponization of a biological agent or toxin
☒	☐	Any other potentially harmful combination of experiments and agents

Plants

Seed stocks: *Report on the source of all seed stocks or other plant material used. If applicable, state the seed stock centre and catalogue number. If plant specimens were collected from the field, describe the collection location, date and sampling procedures.*

Novel plant genotypes: *Describe the methods by which all novel plant genotypes were produced. This includes those generated by transgenic approaches, gene editing, chemical/radiation-based mutagenesis and hybridization. For transgenic lines, describe the transformation method, the number of independent lines analyzed and the generation upon which experiments were performed. For gene-edited lines, describe the editor used, the endogenous sequence targeted for editing, the targeting guide RNA sequence (if applicable) and how the editor was applied.*

Authentication: *Describe any authentication procedures for each seed stock used or novel genotype generated. Describe any experiments used to assess the effect of a mutation and, where applicable, how potential secondary effects (e.g. second site T-DNA insertions, mosiacism, off-target gene editing) were examined.*

ChIP-seq

Data deposition

☐ Confirm that both raw and final processed data have been deposited in a public database such as GEO.

☐ Confirm that you have deposited or provided access to graph files (e.g. BED files) for the called peaks.

Data access links
May remain private before publication.: *For "Initial submission" or "Revised version" documents, provide reviewer access links. For your "Final submission" document, provide a link to the deposited data.*

Files in database submission: *Provide a list of all files available in the database submission.*

Genome browser session
(e.g. UCSC): *Provide a link to an anonymized genome browser session for "Initial submission" and "Revised version" documents only, to enable peer review. Write "no longer applicable" for "Final submission" documents.*

Methodology

Replicates

Describe the experimental replicates, specifying number, type and replicate agreement.

Sequencing depth

Describe the sequencing depth for each experiment, providing the total number of reads, uniquely mapped reads, length of reads and whether they were paired- or single-end.

Antibodies

Describe the antibodies used for the ChIP-seq experiments; as applicable, provide supplier name, catalog number, clone name, and lot number.

Peak calling parameters

Specify the command line program and parameters used for read mapping and peak calling, including the ChIP, control and index files used.

Data quality

Describe the methods used to ensure data quality in full detail, including how many peaks are at FDR 5% and above 5-fold enrichment.

Software

Describe the software used to collect and analyze the ChIP-seq data. For custom code that has been deposited into a community repository, provide accession details.

Flow Cytometry

Plots

Confirm that:

- [ ] The axis labels state the marker and fluorochrome used (e.g. CD4-FITC).
- [ ] The axis scales are clearly visible. Include numbers along axes only for bottom left plot of group (a 'group' is an analysis of identical markers).
- [ ] All plots are contour plots with outliers or pseudocolor plots.
- [ ] A numerical value for number of cells or percentage (with statistics) is provided.

Methodology

Sample preparation

Describe the sample preparation, detailing the biological source of the cells and any tissue processing steps used.

Instrument

Identify the instrument used for data collection, specifying make and model number.

Software

Describe the software used to collect and analyze the flow cytometry data. For custom code that has been deposited into a community repository, provide accession details.

Cell population abundance

Describe the abundance of the relevant cell populations within post-sort fractions, providing details on the purity of the samples and how it was determined.

Gating strategy

Describe the gating strategy used for all relevant experiments, specifying the preliminary FSC/SSC gates of the starting cell population, indicating where boundaries between "positive" and "negative" staining cell populations are defined.

- [ ] Tick this box to confirm that a figure exemplifying the gating strategy is provided in the Supplementary Information.

Magnetic resonance imaging

Experimental design

Design type

Indicate task or resting state; event-related or block design.

Design specifications

Specify the number of blocks, trials or experimental units per session and/or subject, and specify the length of each trial or block (if trials are blocked) and interval between trials.

Behavioral performance measures

State number and/or type of variables recorded (e.g. correct button press, response time) and what statistics were used to establish that the subjects were performing the task as expected (e.g. mean, range, and/or standard deviation across subjects).

Acquisition

Imaging type(s) | *Specify: functional, structural, diffusion, perfusion.*

Field strength | *Specify in Tesla*

Sequence & imaging parameters | *Specify the pulse sequence type (gradient echo, spin echo, etc.), imaging type (EPI, spiral, etc.), field of view, matrix size, slice thickness, orientation and TE/TR/flip angle.*

Area of acquisition | *State whether a whole brain scan was used OR define the area of acquisition, describing how the region was determined.*

Diffusion MRI ☐ Used ☐ Not used

Preprocessing

Preprocessing software | *Provide detail on software version and revision number and on specific parameters (model/functions, brain extraction, segmentation, smoothing kernel size, etc.).*

Normalization | *If data were normalized/standardized, describe the approach(es): specify linear or non-linear and define image types used for transformation OR indicate that data were not normalized and explain rationale for lack of normalization.*

Normalization template | *Describe the template used for normalization/transformation, specifying subject space or group standardized space (e.g. original Talairach, MNI305, ICBM152) OR indicate that the data were not normalized.*

Noise and artifact removal | *Describe your procedure(s) for artifact and structured noise removal, specifying motion parameters, tissue signals and physiological signals (heart rate, respiration).*

Volume censoring | *Define your software and/or method and criteria for volume censoring, and state the extent of such censoring.*

Statistical modeling & inference

Model type and settings | *Specify type (mass univariate, multivariate, RSA, predictive, etc.) and describe essential details of the model at the first and second levels (e.g. fixed, random or mixed effects; drift or auto-correlation).*

Effect(s) tested | *Define precise effect in terms of the task or stimulus conditions instead of psychological concepts and indicate whether ANOVA or factorial designs were used.*

Specify type of analysis: ☐ Whole brain ☐ ROI-based ☐ Both

Statistic type for inference | *Specify voxel-wise or cluster-wise and report all relevant parameters for cluster-wise methods.*

(See Eklund et al. 2016)

Correction | *Describe the type of correction and how it is obtained for multiple comparisons (e.g. FWE, FDR, permutation or Monte Carlo).*

Models & analysis

n/a	Involved in the study
☒	☐ Functional and/or effective connectivity
☒	☐ Graph analysis
☒	☐ Multivariate modeling or predictive analysis
