# Supplementary material for: Oligodendrocyte dysfunction in human age-related cognitive decline
Source: Nat Med. 2026 Aug 25;32(10):3661–71. doi: 10.1038/s41591-026-04608-y (PMC13668666; doi:10.1038/s41591-026-04608-y)
Supplement: Supplementary file 3 [file 41591_2026_4608_MOESM3_ESM.pdf]

nature portfolio

Peer Review File

Oligodendrocyte dysfunction in human age-related cognitive decline

Corresponding Author: Professor Veronique Miron

This file contains all reviewer reports in order by version, followed by all author rebuttals in order by version.

Version 0:

Reviewer comments:

Reviewer #1

(Remarks to the Author)
This is an original and timely paper linking longitudinal cognition from the Lothian Birth Cohort to human white-matter ultrastructure and oligodendroglial states, complemented by an oligodendrocyte-specific Nrf2 knockout in aged mice. The triangulation across EM, snRNA-seq, and genetics is a strength.

The authors propose that steeper cognitive decline is associated with greater oligodendrocyte numbers, enrichment of specific oligodendrocyte states (Oligo3/Oligo5), and reduced NRF2 activity; that an oligodendrocyte-specific Nrf2 knockout in mice recapitulates the human ultrastructural phenotype; and that oligodendrocyte dysfunction "drives" the neuropathology of cognitive decline with NRF2 as a therapeutic target.

The multimodal approach is notable, but in its current form the interpretation of the data is not convincing and the translational relevance remains underdeveloped.

Alternative interpretation:
An equally plausible interpretation is an axon-first model. The human readouts that correlate most strongly with cognitive decline are axonal: (i) a left-shift in axon calibre distributions, and (ii) increased frequency of degenerating/empty profiles. Both are canonical features of a large-axon-predominant axonopathy. The lower g-ratio observed only in >1µm fibres is also consistent with axonal shrinkage outpacing myelin remodelling: surviving large axons reduce in calibre while their pre-existing sheaths fail to fully adapt, giving the appearance of relatively thick myelin.

The snRNA-seq signal (more oligodendrocytes overall, with enrichment of Oligo3/5 states characterised by stress, lipid, and autophagy signatures) can be read as reactive expansion and phenotypic shift of glia responding to axonal injury, rather than proof that oligodendrocytes are the initiating lesion. Similarly, the reduction in NRF2 positivity in an expanded oligodendrocyte pool is consistent with an exhaustion model in chronically activated glia, not necessarily primary NRF2 failure.

The oligodendrocyte-specific Nrf2 cKO in aged mice demonstrates that oligodendroglial stress is sufficient to reproduce the morphological end-state (smaller axons, lower g-ratio) without altering oligodendrocyte numbers. This keeps an oligodendrocyte-first mechanism on the table, but does not resolve directionality in humans: the human data remain equally compatible with large-axon degeneration as the primary event that drives secondary oligodendroglial responses.

Additional major comments:

1) Fig. 2C and 2G-H. These plots relate raw nuclei counts per donor to cognitive slope. Counts are heavily influenced by capture efficiency, sequencing depth, and day-to-day processing variability, and therefore cannot be interpreted as biological abundance. Covariates were included in the DEG models for subclusters, but not in these abundance analyses. Without expressing data as proportions and modelling compositional structure with batch covariates, it remains uncertain whether the reported increases in oligodendrocytes and Oligo3/Oligo5 states reflect genuine biology or technical variability. The IF validation supports the direction of effect, but the sequencing results on their own are not statistically secure.

2) Fig. 1G; Fig. 3H. Axon-diameter and g-ratio distributions were compared using Kolmogorov–Smirnov tests. However, the

biological units are donors/animals, not the hundreds of individual axons traced within each case. Pooling axons inflates sample size and risks false significance, so the reported p-values may overstate the strength of the distributional differences. In addition, the effect attributed to "large axons" relies on an arbitrary >1 µm cut-off; without modelling diameter continuously, it is unclear whether the association is genuinely restricted to large fibres or an artefact of binning. As a result, the claim that large-axon g-ratio specifically tracks decline is not convincingly supported by the current analysis.

3) Some key associations (mean axon diameter, g-ratio of large axons, degenerating profiles, oligodendrocyte counts) rest on very small samples with effect sizes around $r \sim 0.6$ and marginal P values (0.02–0.04). As presented, these correlations are shown without confidence intervals, covariate adjustment, or correction for multiple comparisons across related outcomes. Without these safeguards, the robustness of the associations is uncertain and they may not survive adjustment for age at death, sex, post-mortem interval, or co-pathologies. Given the small n, the risk that one or two donors drive the effects is high, leaving the strength of these correlations open to question.

4) The evidence for NRF2 involvement is weaker than implied. The upstream "NRF2 inhibition" claim depends on IPA analyses in which the DEG threshold was relaxed to $P < 0.1$ to generate sufficient input genes. The protein-level data quantify the proportion of oligodendrocytes positive for NRF2, which demonstrates correlation but not pathway activity. Taken together, these findings indicate that reduced NRF2 activity may be a feature of the oligodendrocyte response, but they do not establish it as the initiating lesion. The aged oligodendrocyte-specific Nrf2 knockout demonstrates sufficiency for the morphological phenotype, but without conduction or behavioural readouts, it does not link NRF2 loss to cognition. As presented, the claim that oligodendrocytes "drive" cognitive decline is therefore stronger than the data support.

5) The authors state that the work "overturns dogma". Given the associative nature of the human data and absence of any functional and/or behavioural readouts, this language overstates the strength of the evidence.

Minor points:

6) The manuscript would benefit from more analysis of potential neuronal contributions to the observed oligodendroglial changes. The finding of apparently thicker myelin on large axons raises the possibility that neuronal stress or degeneration could cue oligodendrocyte responses. If neuronal nuclei were captured in sufficient numbers, even exploratory DEG analyses might help situate the oligodendrocyte changes within the broader neuron-glia context. This would also help to evaluate alternative models, such as neuron-first/axon-first mechanisms.

7) It is not clear to me whether g-ratios were calculated from circular approximations or area measurements, or whether resin shrinkage was corrected. Without this information the quantitative accuracy of the EM data is uncertain. Similarly, classification of "degenerating" profiles appears subjective.

8) The text does not state whether post-mortem interval, fixation quality, or neuropathological covariates were included in the donor-level correlations (they appear not to be). In their absence, the possibility of confounding remains.

9) The donor-level regression slopes and intercepts are not linked to tissue metrics in a supplementary table. Without this, the reproducibility and transparency of the analyses are limited.

10) Image quantification thresholds were optimised per case. While this may have been necessary due to staining variability, it raises questions about comparability across donors. Without demonstrating robustness to threshold choice or applying normalised fixed thresholds, the reproducibility of these measurements is uncertain.

11) "Central white matter" was analysed in humans whereas corpus callosum was analysed in mice; It is not clear to what extent findings from these two regions can be directly generalised across species.

Reviewer #2

(Remarks to the Author)
The present study leverages a remarkable longitudinal cohort of human subjects tested for cognitive function across their lifespans, together with postmortem brain analyses (EM, snRNA-seq) from a subset of the same individuals. This reveals dysmyelination and increased oligodendrocyte numbers correlated to cognitive decline in aging. These human studies highlighted NRF2 as a mechanism that could account for the poor cognition-associated oligodendroglial and myelin phenotype. Mouse modeling revealed that conditional knockout of NRF2 in oligodendrocytes recapitulated the oligodendroglial and myelin phenotype seen in humans with cognitive decline.

This is a beautiful and important study that strongly links dysmyelination in aging with cognitive decline and identifies a molecular mechanism to account for this observation. The data from the human subjects work, from cognitive assessments throughout life to postmortem EM and snRNA seq are exceptionally strong, and the mouse modeling work to validate the NRF2 hypothesis gleaned from human tissue studies is convincing. I have the following suggestions to improve this already excellent manuscript.

Comments:
1. Figure 1 J shows myelin decompaction (or split artifact) - were g-ratio measurements affected by this splitting between wraps? Is the myelin really "thicker", or is it decompacted?

2. Do the oligodendrocyte-specific NFR2-KO mice have cognitive deficits?

Minor suggestions
3. PMID 24727982 should be included in the references for de novo myelin plasticity

Reviewer #4

(Remarks to the Author)
This is a very interesting and potentially very important study. Bringing together cognitive information on aging humans, neuropathology, and a level of mechanistic insight is a courageous undertaking of the sort that is necessary to reveal a genuinely meaningful understanding of the ageing brain. While the authors are to be congratulated on this undertaking and having pulled off a truly impressive study, there are a number of provisos regarding technical aspects of the study that render emphatic conclusions difficult to make.
The analysis of myelinated axons in postmortal human tissue is an heroic endeavour and one that is necessary to address the study's central questions. It is however an undertaking that is fraught with technical difficulty: how confident can one be in distinguishing events that took place during life from those that took place afterwards (tissue deterioration, fixation and processing artefacts etc). These are, of course, not issues that the authors can address with additional experiments, but I think it would be appropriate for the conclusions drawn to be couched inappropriate caveats – even in the results section. Related to this issue is the concept of 'thicker myelin' – a term which could be construed to me to mean that the myelin sheath has increased in diameter. It is more likely that the axon has atrophied and the myelin sheath has stayed the same as it was as it ever was. The authors should perhaps use a more nuanced way describing the changes that they are observing. If I have understood correctly, what the authors are describing in the first section of the results is a generalised primary axonal atrophy which is more advanced in larger diameter fibres. Does it not then imply that the key correlation with cognitive decline is loss of axons – in which case would this be a particularly novel observation, especially if the axonal atrophy is as a result of neuronal loss/grey matter atrophy? I suppose to some extent this question is answered in the oligodendrocyte NRF2 deletion studies in mice. Nevertheless, this is an important issue that the authors will need to address.
Given these caveats I'm not sure that the authors can really make the claim that oligodendrocytes are a driver of neuropathology. This is perhaps overstating it somewhat and it may be better for them to talk about oligodendrocytes being a key contributor to neuropathology.
Minor issues
The increased density of oligodendrocytes does not imply de novo generation of new cells. Density readouts will be in part due to absolute numbers of cells but also due to changes in other cell types and parenchymal changes. This should be acknowledged
In the introduction, I wonder whether references 8 to 11 really are the most appropriate ones to support the claim that has been made. Can I suggest the authors look carefully at this and perhaps make some revisions?
I am not sure that the term central white matter is one that would be widely understood (for my part, I'm not exactly certain what is meant by this term). Perhaps the authors could be more explicit about which anatomical region(s) within the brain they are referring to with this term.

Version 1:

Reviewer comments:

Reviewer #1

(Remarks to the Author)
The authors have addressed my main concerns in a satisfactory manner. In particular, they have added appropriate analyses to test the alternative axon first interpretation, have strengthened the treatment of technical and statistical confounds, and have included new behavioural data in the oligodendrocyte specific Nrf2 conditional knockout mice. The human data necessarily remain associative and do not definitively establish directionality, but the revised manuscript now acknowledges this limitation more appropriately and the language has been sufficiently tempered. I therefore have no further major concerns. I have only two minor suggestions. First, the authors should ensure that any probability distribution analyses are presented descriptively and are not used as the primary basis for statistical inference. Second, the raw nuclei count correlations in Fig. 2C should either be clearly labelled as exploratory or the associated significance values should be removed, given the authors' own acknowledgement that sequencing based abundance estimates are not statistically secure on their own.

Reviewer #2

(Remarks to the Author)
The authors have comprehensively addressed my comments. This is a beautiful and important paper that represents a landmark contribution to the field.

Reviewer #4

(Remarks to the Author)
My apologies to the authors for my slow response to their resubmission. All the points I raised have been addressed in a thorough and scholarly way and I thank the authors for the evident care they have taken in considering each of them. I have no further comments to make on this excellent study and manuscript.

Open Access This Peer Review File is licensed under a Creative Commons Attribution 4.0 International License, which permits use, sharing, adaptation, distribution and reproduction in any medium or format, as long as you give appropriate credit to the original author(s) and the source, provide a link to the Creative Commons license, and indicate if changes were made.
In cases where reviewers are anonymous, credit should be given to 'Anonymous Referee' and the source.
The images or other third party material in this Peer Review File are included in the article's Creative Commons license, unless indicated otherwise in a credit line to the material. If material is not included in the article's Creative Commons license and your intended use is not permitted by statutory regulation or exceeds the permitted use, you will need to obtain permission directly from the copyright holder.
To view a copy of this license, visit https://creativecommons.org/licenses/by/4.0/

We appreciate positive comments from all 3 reviewers that our manuscript is '*truly impressive*', '*original and timely*', and '*reveal[s] a genuinely meaningful understanding of the ageing brain*', that the multimodal approach is '*notable*' and '*a strength*', and that the data is '*exceptionally strong*' and '*convincing*'. We have now addressed the reviewers' comments with a substantial amount of new data which have significantly improved the translational relevance and supported the interpretations drawn from our study, with **15 new experiments resulting in 38 new figure panels**. A summary of new data is found below, followed by a point-by-point response to individual reviewer comments. Changes in the manuscript have been highlighted in yellow to facilitate review.

<u>Summary of new data:</u>

1) **No indications of overt primary axonopathy in human cognitive decline in aging:**
To address the reviewer suggestions that oligodendrocyte/myelin changes in cognitive decline may be driven by *primary* axonopathy (either degeneration or shrinkage), we assessed 4 well-established features that precede primary axonopathy by additional ultrastructural and immunofluorescent analyses of aged human white matter. Indications of severe axon damage/degeneration (axonal spheroids) or shrinkage (dark axoplasm from condensation of cytoskeletal elements) were not detected in any case. Indications of axon stress (increased mitochondrial size) or damage (increased SMI32 expression levels) did not significantly differ with severity of cognitive decline. Therefore, our data does not suggest primary axonopathy, supported by our prior data indicating no differentially expressed genes in neuronal clusters based on cognitive decline severity.

2) **Oligodendrocyte NRF2 regulates cognitive ability over time in aging:**
We carried out cognitive tests on aged oligodendrocyte-specific NRF2 conditional knockout mice (cKO). We used the Morris Water Maze given its sensitivity to detect cognitive changes consequent to aging or white matter alterations, and the capacity to look at changes in cognition *over time* as done in the human cohort. Control mice showed a 57% improvement in percentage of time spent in the target quadrant over 4 days of testing. In contrast, this was significantly blunted to 27% in cKOs, with a relative decrease in absolute percentage of time in the target quadrant in late test trial days vs controls. This indicates that loss of NRF2 in oligodendrocytes is sufficient to attenuate cognitive improvement over time in aging. We observed considerable variation in cognitive performance across genotypes, and significantly associated worse performance with the neuropathological features of severe cognitive decline in humans (reduced myelinated axon size, thicker myelin); these features were aggravated in late aging in the cKO vs controls.

3) **Ruling out technical variability in oligodendrocyte/myelin associations with severity of cognitive decline:**
We carried out new analyses to rule out the potential contribution of technical variability to our conclusions. First, we now provide a matrix which shows no significant correlations between neuropathology outcomes and post-mortem interval, brain weight, brain pH, age at death, sex, or post-mortem evidence of neurodegenerative disease (Small Vessel Disease or Alzheimer's Disease). For electron microscopy analysis, we rule out potential electron microscopy artefacts of myelin decompaction due to tissue processing, by complementing this with label-free imaging of compact myelin using SCoRe microscopy. For snRNAseq validation, we now include correlations between clinical and technical covariates and oligodendrocyte/NRF2+ oligodendrocyte abundance. For immunofluorescence, we performed quality control experiments to validate our approach in accounting for differing background fluorescence between cases.

4) **Strengthened statistical analyses:**
We provide objective reasoning for the binning thresholds used in axon size or cognitive performance (human and mouse), provide data as both grouped averages and as a continuous variable, and include power calculations that indicate the robust statistical power of our sample sizes. We now clearly state confidence intervals, correction for multiple comparisons, and covariate adjustments in the methods.

__Response to reviewer comments:__

Reviewer #1:

This is an original and timely paper linking longitudinal cognition from the Lothian Birth Cohort to human white-matter ultrastructure and oligodendroglial states, complemented by an oligodendrocyte-specific Nrf2 knockout in aged mice. The triangulation across EM, snRNA-seq, and genetics is a strength. The authors propose that steeper cognitive decline is associated with greater oligodendrocyte numbers, enrichment of specific oligodendrocyte states, and reduced NRF2 activity; that an oligodendrocyte-specific Nrf2 knockout in mice recapitulates the human ultrastructural phenotype; and that oligodendrocyte dysfunction "drives" the neuropathology of cognitive decline with NRF2 as a therapeutic target. The multimodal approach is notable, but in its current form the interpretation of the data is not convincing and the translational relevance remains underdeveloped.

1.1 Alternative interpretation: An equally plausible interpretation is an axon-first model. The human readouts that correlate most strongly with cognitive decline are axonal: (i) a left-shift in axon calibre distributions, and (ii) increased frequency of degenerating/empty profiles. Both are canonical features of a large-axon-predominant axonopathy. The lower *g*-ratio observed only in >1 µm fibres is also consistent with axonal shrinkage outpacing myelin remodelling: surviving large axons reduce in calibre while their pre-existing sheaths fail to fully adapt, giving the appearance of relatively thick myelin.

We agree with the reviewer that the left-shift in axon calibre distributions, increased axon degenerating profiles, and lower *g*-ratio (increased myelin thickness) are features that could be consistent with a primary axonopathy. To investigate this possibility, we conducted several new analyses of the human white matter.

First, we assessed ultrastructural features known to precede axonopathy: increased mitochondrial size, axonal spheroids, and dark axoplasm - the latter resulting from axon shrinkage-induced condensation of cytoskeletal elements (*Schaffner et al, 2023 Nature Neurosci; Yong et al 2022, Mol Cell Neurosci; Kiryu-Seo et al, 2016, Sci Rep; Barrientos et al, 2011, J Neurosci; Webster, 1962, J Cell Biol; Ferreirinha et al, 2004, JCI; Sasaki et al, 2004, Acta Neuropathol*). Importantly, these features are not influenced by inter-sample variations in tissue quality nor stochasticity of sampling along the axon, and represent hallmarks of axon pathology which occur early (mitochondrial size) and late (spheroids) in degeneration. We did not detect axonal spheroids nor dark axoplasm in any sample regardless of cognitive decline severity; this contrasts the abundant axonal spheroids we previously detected in human white matter of the disorder ALSP (*McNamara et al, 2023, Nature*). Axonal mitochondrial size did not significantly differ with severity of cognitive decline (**new Fig.1S,T**). As a control for our capacity to detect increased mitochondrial size, middle-aged adult brain samples were included as a comparison, which confirmed that aging in general causes an increase (**new Fig.1T**) as expected in aging white matter (*Stahon et al, 2016, J Neurosci*). Second, we used immunofluorescence to assess expression levels of SMI-32, commonly used as a readout of axonal pathology in diseased human brain (e.g. *Schultz et al, 2017, Glia*). As SMI-32 can also pick up healthy neuronal cell bodies (*Campbell and Morrison, 1989, J Comp Neurol*), staining surrounding Hoechst+ nuclei was excluded to specifically quantify axon pathology (please note that all ovoid SMI-32 staining was associated with a Hoechst+ nucleus, and therefore did not represent axonal spheroids). Less than 10% of tissue area was positive for SMI-32 across cases, and there was no significant difference based on degree of cognitive decline (**new Fig.1U,V**).

These findings suggest that there is no overt primary axonopathy/stress in corpus callosum in association with severity of human cognitive decline in aging. This is consistent with data included in our first submission, where thicker myelin was not observed on the smaller axons (**Fig.1N,O**); if axon shrinkage was occurring in large axons thereby causing the increase in small axons, we would expect thicker myelin on the small axons as well. Notably, although thicker myelin can cause axonal stress, axonal stress/pathology has been shown *not* to lead to thicker myelin (*Ineichen et al, 2021, J Neurosci Res*). In addition, we had noted no significant changes in neuronal gene expression in association with severity of cognitive decline (**Fig.S3D**). The neuronal clusters were of equivalent size to those in other human brain sequencing studies (*Jakel et al, 2019, Nature*; *Tran et al, 2021, Neuron*), including where pathology

was seen to influence neuronal gene expression (*Tran et al, 2021, Neuron*), indicating that the capture of neuronal nuclei would have been sufficient to detect significant changes associated with stress. Although this does not rule out gene expression changes in neuronal (or other cell type) nuclei in other brain regions - which we now acknowledge in the Discussion - together with our neuropathological analyses, it does suggest that readouts of potential primary neuronal changes in corpus callosum do not appear to associate with severity of cognitive decline.

Furthermore, our findings are in contrast with those in human primary axonopathies and their associated mouse models. For instance, in Giant Axonal Neuropathy (GAN), axons have spheroids, thin myelin sheaths, and undergo robust demyelination (reviewed in *Johnson-Kerner et al, 2014, Muscle Nerve*), none of which are evident in our cohort. Increased mitochondrial size occurs prior to axonal shrinkage and degeneration in the primary axonopathies Hereditary Spastic Paraplegia and Motor Neuron Disease (*Ferreirinha et al, 2004, JCI; Sasaki et al, 2004, Acta Neuropathol*), yet this was not evident in the corpus callosum with severe cognitive decline. Altogether, analysis of aged human brain at the ultrastructural, histopathological, and transcriptional levels do not provide evidence for primary axonopathy in the corpus callosum in association with severity of cognitive decline.

1.2 The snRNA-seq signal (more oligodendrocytes overall, with enrichment of Oligo states characterised by stress, lipid, and autophagy signatures) can be read as reactive expansion and phenotypic shift of glia responding to axonal injury, rather than proof that oligodendrocytes are the initiating lesion. Similarly, the reduction in NRF2 positivity in an expanded oligodendrocyte pool is consistent with an exhaustion model in chronically activated glia, not necessarily primary NRF2 failure.

We appreciate the alternative explanation for our observed oligodendrocyte and myelin changes. As post-mortem tissue provides only a snapshot in time, it is not possible with human samples to understand when oligodendrocytes were generated, and whether the NRF2 reduction represents a primary failure or not. However, in the absence of evidence of primary axonal pathology (new Fig.1R-U) together with oligodendrocyte production known to be limited in aging, we believe it unlikely that our results reflect reactive oligodendrocyte expansion due to primary axonal injury. Moreover, prior work indicates that primary axon pathology in mice leads to a loss of oligodendrocytes, rather than an increase (*Sun et al, 2010, Glia; Barres et al, 1993, Curr Biol*).

Although a timeline of causative pathologies is impossible to decipher in post-mortem human tissue, particularly in a cohort with a small age differential (due to the individuals being born in the same year), the functional testing of this timeline in mice robustly supports our conclusions. Modeling a primary NRF2 failure in oligodendrocytes by conditional knockout is sufficient to induce the myelin and myelinated axon abnormalities associated with worse cognitive decline in human aging (**Fig.3L-O**), indicating that oligodendroglial NRF2 downregulation (therefore 'primary failure') is at least capable of contributing to this pathology. We now complement this data with cognitive assays indicating an attenuated capacity of cKOs to improve cognitive performance over time (**new Fig.3D-K**), functionally associating oligodendrocyte reduction in NRF2 with worse cognitive function in aging.

1.3 The oligodendrocyte-specific Nrf2 cKO in aged mice demonstrates that oligodendroglial stress is sufficient to reproduce the morphological end-state (smaller axons, lower *g*-ratio) without altering oligodendrocyte numbers. This keeps an oligodendrocyte-first mechanism on the table, but does not resolve directionality in humans: the human data remain equally compatible with large-axon degeneration as the primary event that drives secondary oligodendroglial responses.

Please see points 1.1 and 1.2 for our new data that does not support primary axon pathology (**new Fig.1S-V**). Importantly, we are not claiming that primary axon pathology does not happen in aging human brain. Rather, we are not seeing evidence that there is *differential* early axon stress in corpus callosum in association with severity of cognitive decline.

1.4 Fig. 2C and 2G-H [*note 2G-H now removed*]. These plots relate raw nuclei counts per donor to cognitive slope. Counts are heavily influenced by capture efficiency, sequencing depth, and day-to-day processing variability, and therefore cannot be interpreted as biological abundance. Covariates were included in the DEG models for subclusters, but not in these abundance analyses. Without expressing data as proportions and

modelling compositional structure with batch covariates, it remains uncertain whether the reported increases in oligodendrocytes and Oligo states reflect genuine biology or technical variability. The IF validation supports the direction of effect, but the sequencing results on their own are not statistically secure.

We agree with the reviewer that raw nuclei counts per donor can be biased by technical artefacts and cannot *on their own* be used to draw conclusions, and appreciate the acknowledgement that our immunofluorescence data supported the interpretation of increased oligodendrocytes in association with severe cognitive decline. Nonetheless, we have removed significance values from **Fig.2C** as our interpretation of this data is hypothesis-generating, and emphasize the validation of increased oligodendrocytes by immunofluorescence in the text. In addition, we have de-emphasized the importance of Oligo state abundance correlations with cognitive decline severity. Rather, we now focus on DEGs within each oligodendrocyte state when comparing mild vs severe cognitive decline, and found that only Oligo4 and Oligo5 clusters had a significant number of DEGs (167 and 113, respectively) (**new Fig.2G,H**), whereas all other oligodendrocyte clusters had 0-2). IPA analysis of the DEGs altered with cognitive decline severity in Oligo4 or 5 indicated that the *only shared significant upstream regulator* was NRF2, predicted to be downregulated in both clusters (**new Supplemental Sheet 1**). This is consistent with our validation data at the protein level showing statistically significant downregulation of NRF2+ oligodendrocytes with more severe cognitive decline (**Fig.2M,N**).

To understand if technical variance could indeed explain the effect of increased oligodendrocytes we saw by sequencing, we revisited our original fastq data and sequencing experimental design. Importantly, we found that the original number of total clusters passing the filter from our raw fastq files was not significantly different between mild versus severe cognitive decline (P=0.23, Student's t-test), nor was this significantly different between our two sequencing batches (P =0.5; Student's t-test). The average saturation was 82.1%, and the number of total reads per sample ranged from 233-345 million, showing high coverage across all samples. There were no changes in nuclei quality associated with cognitive decline status, as assessed by average feature counts, UMIs, and mitochondrial gene percentage (**Fig.S2G-I**), and over 97% of the nuclei passed QC thresholds. Furthermore, we noted that samples processed sequentially on the same day and sequenced on the same 10X chip in the same batch yielded both the second-lowest and the highest number of clusters passing filter, highlighting that day-to-day variation, timing, and batch were unlikely to have significantly altered nuclei number. We took care to limit batch variability during the experimental design by ensuring that each sequencing batch contained 8 individuals as evenly distributed as possible across cognitive decline status, sex, and age at death, and the nuclei isolation protocol for both batches was run over 48 hours for all samples to limit day-to-day variation. Buffers were made fresh prior to the experiment and the same buffer batch was used for all samples. All 16 original samples were prepared into the same library and sequenced together, limiting batch variability which can occur from sequencing on different Illumina P3 flow cells. We also note that when we performed integration of our Seurat objects, we utilised the Canonical Correlation Analysis method of batch correction to further reduce any technical variation. Alongside increased raw counts of oligodendrocytes, we also observed a similar correlation between oligodendrocyte proportions (of total detected nuclei) and severity of cognitive decline (**Fig.S2K**).

We also appreciate the reviewer's important point that there could be other factors which are impacting dysfunctional oligodendrocyte numbers which we did not account for in the sequencing data. To assess this, we correlated oligodendrocyte densities from protein validation (given this is the more statistically secure result) against technical (post-mortem interval, brain weight, brain pH) and biological (age, sex, absolute cognition at age 70, AD and SVD pathology) covariates, and noted no other variables significantly associated with the changes in oligodendrocyte densities or NRF2+ oligodendrocyte proportions (**new Fig.2O**).

1.5 Fig. 1G; Fig. 3H [*note now Fig.1K, 3M*]. Axon-diameter and *g*-ratio distributions were compared using Kolmogorov–Smirnov tests. However, the biological units are donors/animals, not the hundreds of individual axons traced within each case. Pooling axons inflates sample size and risks false significance, so the reported p-values may overstate the strength of the distributional differences. In addition, the effect attributed to "large axons" relies on an arbitrary >1 µm cut-off; without modelling diameter continuously, it is unclear whether the

association is genuinely restricted to large fibres or an artefact of binning. As a result, the claim that large-axon *g*-ratio specifically tracks decline is not convincingly supported by the current analysis.
We agree with the reviewer that running statistical comparisons when including multiple myelinated axons amplifies the risk of false significance, and agree that statistics on the average axon size and *g*-ratio per sample are required. We now provide the data as the average per sample, both as a grouped plot based on cognitive performance for humans, and cognitive performance or genotype for mice (**Fig.1L,N; new Fig.3J,K,N,O**), and as a continuous variable of these averages across the spectrum of possible cognitive scores for humans (**Fig.1M,O**); this is from analysis of >100 myelinated axons quantified/human and >200 myelinated axons/mouse. We do however believe that the probability density distribution is visually effective for noting the shift in myelinated axon size, yet to address these points we have removed the statistical analysis from these plots. Importantly, the significant relationships between cognitive decline and smaller axon size or *g*-ratio remain significant when plotted as averages, and when binned or plotted as a continuous variable, indicating that these are robust effects.

With regards to binning axons using a 1 μm threshold, we note that this threshold is not arbitrary but rather functionally relevant; it is the threshold at which axons in the human corpus callosum have the thickest myelin under homeostasis (*Liewald et al, 2014, Biol Cybern*) and at which axons are more vulnerable to pathological increases in myelin thickness in human disease (*McNamara et al, 2023, Nature*). It is a commonly used threshold for large axons in the myelin field. We have now indicated the rationale for this threshold in the methods.

1.6 Some key associations (mean axon diameter, *g*-ratio of large axons, degenerating profiles, oligodendrocyte counts) rest on very small samples with effect sizes around r ~ 0.6 and marginal P values (0.02–0.04). As presented, these correlations are shown without confidence intervals, covariate adjustment, or correction for multiple comparisons across related outcomes. Without these safeguards, the robustness of the associations is uncertain and they may not survive adjustment for age at death, sex, post-mortem interval, or co-pathologies. Given the small n, the risk that one or two donors drive the effects is high, leaving the strength of these correlations open to question.
We appreciate the reviewer's comment on the sample sizes, which is out of our control given that the Lothian Birth Cohort study is ongoing and the samples available depend on the stochasticity of timing of individuals dying. However, to address the reviewer's concern about the robustness of observations, we performed power calculations on our datasets by the normal approximation method using a two-sided confidence interval of 95% (now included in the quantification and statistics section of the methods). We noted that all statistical comparisons mentioned between mild vs severe cognitive decline groups were powered at over 80% (82-99%) - the established threshold for confidence in statistical analyses.

We note we had included confidence intervals in all our correlations in the original submission (indicated by the pink shading in the correlation plots **Fig.1C,D**; grey shading in **Fig.1F,M,O,R; 2C**), and have now made the reference to these more explicit in figure legends. We also note we had included covariate adjustment for age at death and sex, and correction for multiple comparisons across outcomes in our original sequencing analysis. To further safeguard that the neuropathological features associated with cognitive decline were not associated with other variables, we have now assessed the strength and significance of correlations between our neuropathological results and additional covariates such as technical variables (post-mortem interval, brain weight, brain pH), co-pathologies (post-mortem features of Alzheimer's disease or Small Vessel Disease), clinical features (sex, age at death), and other cognitive features (age 11 IQ, absolute cognition at age 70, and extrapolated cognition at death as testing is only every 3 years) (**new Fig.2O; new Fig.S1B**). We note no significant correlations between these covariates and oligodendrocyte densities, NRF2+ oligodendrocyte densities, degenerating axon profiles, *g*-ratio, and myelinated axon density or proportion. We did see an association between Braak stage and axon diameter, suggesting that smaller axons may be a shared pathological feature between age-associated cognitive decline and Alzheimer's Disease pathology. We also saw significant associations between average axon mitochondrial size and age at death, absolute cognition at age 70, and extrapolated cognition at death, suggesting mitochondrial stress may be a readout of absolute cognitive ability – but importantly not associated with the degree of cognitive decline (**new Fig.2O; new Fig.S1B**).

1.7 The evidence for NRF2 involvement is weaker than implied. The upstream "NRF2 inhibition" claim depends on IPA analyses in which the DEG threshold was relaxed to P < 0.1 to generate sufficient input genes. The protein-level data quantify the proportion of oligodendrocytes positive for NRF2, which demonstrates correlation but not pathway activity. Taken together, these findings indicate that reduced NRF2 activity may be a feature of the oligodendrocyte response, but they do not establish it as the initiating lesion. The aged oligodendrocyte-specific Nrf2 knockout demonstrates sufficiency for the morphological phenotype, but without conduction or behavioural readouts, it does not link NRF2 loss to cognition. As presented, the claim that oligodendrocytes "drive" cognitive decline is therefore stronger than the data support.

We clarify that the low number of DEGs acquired in our statistical analyses was a result of several robust covariates being included to identify novel causes of cognitive decline, as we had explained in the results text. Nevertheless, we agree that validation of transcriptomic/bioinformatic predictions is important, and immunofluorescent analysis of NRF2 protein-expressing oligodendrocytes confirmed a decreased proportion in more severe cognitive decline (**Fig.2M,N**). With regards to NRF2 activity, this is challenging to assess because it is a transcription factor for hundreds of genes, which are typically also regulated by other factors. As such, we consider it outside the scope of this study to assess all downstream targets of NRF2 to assess its activity. We agree that human analyses indicate association but cannot test causation. Rather, we consider the oligodendrocyte-specific conditional knockout of NRF2 the appropriate test of causation for the activity of NRF2.

As requested, we assessed the causation of oligodendrocyte downregulation of NRF2 in impaired cognitive performance in aging, using the oligodendrocyte-specific conditional knockout. This was done at 12 months of age, when cognition begins to be impaired in wildtype mice (e.g. *Wang et al, 2020, Nature Neurosci*). We first performed the Open Field test to screen for major motor or anxiety effects that might confound behavioural readouts, which indicated that females had confounding altered anxiety (**new Fig.S4A,B**), and were thus excluded from further tests. We used the Morris Water Maze (**new Fig.3D**) to test for cognitive performance *over time*, facilitating comparison to cognitive measures in the human cohort. This platform is highly sensitive to detecting cognitive impairment in aging (*Wang et al, 2020, Nature Neurosci*) or alterations to corpus callosum oligodendrocytes (*Chen et al, 2021, Neuron; Steadman et al, 2020, Neuron*). While it is a hippocampus-based task, white matter connections through the corpus callosum (e.g. to prefrontal cortex) are required to retrieve spatial information and to support search strategy (*Woolley et al, 2013, PNAS; Sang Jo et al, 2007, J Neurosci; Rossato et al, 2015, Neurobiol Learn Mem*).

Over the course of the testing phase, all mice showed an increase in the percentage of time spent in the target quadrant containing the platform, indicating spatial learning and memory encoding (**new Fig.3E**). As expected, controls showed a robust improvement in the percentage of time spent in the target quadrant over test trial days, increasing by 57% by day 4 vs day 1. In contrast, conditional knockouts showed an attenuated increase in percentage of time spent in the target quadrant over time, with only a 27% improvement by day 4, which was significantly less than controls (**new Fig.3E-F**). Importantly, blunted improvement was not due to the conditional knockouts reaching the maximum possible percentage time in the target quadrant in prior days, as the absolute percentage of time spent in the target quadrant was lower than controls on days 3 and 4 (**new Fig.3E**).

Relatively worsened learning over time in the conditional knockouts was observed despite showing a surprisingly shorter latency to reach the target quadrant vs controls over all test days (**new Fig.S4E**). As this did not reflect faster swim speed in the testing phase (**new Fig.S4F**), we next investigated the directness of paths taken, i.e. search strategy. To quantify search strategy, we applied the approach established by *Quintanilla Sanchez et al, 2023, Biomedicine* which allocates search paths in the Morris Water Maze into 8 categories to calculate a 'score' - where more direct spatial strategies correspond to a higher score vs less direct non-spatial strategies (**new Fig.S4G**). This revealed that conditional knockouts engaged a more directed strategy (higher average score/day) from day 1 (even though they should not be aware of the location of the platform yet, as the platform location moved during the acclimatisation phase), however conditional knockouts did not improve their strategy over time as much as the controls (**new Fig.S4H,I**). Again, this was not due to the conditional knockouts already having reached the maximum 'directness' in search strategy: i) the maximum score possible is 6 and by the last test day the conditional knockouts had an average score of 4, and ii) both conditional knockouts and controls only ever reached the max. score in ~10%

of test trials (**new Fig.S4G**). In summary, these findings indicate that aged oligodendrocyte NRF2 conditional knockout mice show a blunted capacity to improve cognitive performance over time. This directly links NRF2 loss in oligodendrocytes to cognitive performance for the first time.

While conditional knockouts showed attenuated cognitive improvement over time, we noted a large degree of individual variation in performance across both genotypes. To correlate individual cognitive performance with white matter correlates, as was done in the human, we binned animals based on whether performance over time was better or poorer than average (**new Fig.3G**). When tissue was assessed by electron microscopy, there was clear variability in ultrastructural features of myelinated axons between better and poorer performers (**new Fig.3I**). Both axon diameter and *g*-ratio were significantly reduced in poorer performers (**new Fig.3J,K**), indicating that smaller axons and thicker myelin are correlates of worse cognitive performance in aging mice. In late aging (18 months), myelinated axon diameter and *g*-ratio were significantly reduced in conditional knockouts (**Fig.3L-O**), suggesting that loss of NRF2 in oligodendrocytes exacerbates the neuropathological features associated with age-related cognitive decline in both mouse and human.

1.8 The authors state that the work "overturns dogma". Given the associative nature of the human data and absence of any functional and/or behavioural readouts, this language overstates the strength of the evidence.
We appreciate the suggestion to temper our language. Although we believe our new data with functional/ behavioural readouts in the mouse supports that dysfunctional oligodendrocytes directly contribute to cognitive impairment with aging (**new Fig.3B-K**), we have softened our statements throughout the manuscript.

Minor points:
1.9 The manuscript would benefit from more analysis of potential neuronal contributions to the observed oligodendroglial changes. The finding of apparently thicker myelin on large axons raises the possibility that neuronal stress or degeneration could cue oligodendrocyte responses. If neuronal nuclei were captured in sufficient numbers, even exploratory DEG analyses might help situate the oligodendrocyte changes within the broader neuron-glia context. This would also help to evaluate alternative models, such as neuron-first/axon-first mechanisms.
Our prior submission included DEG analysis on neuronal subclusters and found none between mild and severe cognitive decline, even when lowering adjusted p value threshold to 0.1, as was done for the pathway analysis with oligodendrocytes (**Fig.S3E**). We also note that the average sequencing saturation was 82.1% across samples, and the number of total reads per sample ranged from 233-345 million, indicating the lack of neuronal DEGs is unlikely to have resulted from insufficient sequencing depth. Indeed, our capture of neuronal nuclei was in an appropriate range to sufficiently identify DEGs in neurons between groups, in line with detected nuclei frequencies in a prior human brain snRNAseq study (*Tran et al, 2021, Neuron*), as indicated in response 1.1. This complements our new data where we did not detect differential abundance of features known to precede axonopathy between these groups (see response 1.1, **new Fig.1S-V**). Importantly, we are not suggesting that neurons do not change their gene expression with aging, but rather that neuronal gene expression in the corpus callosum does not associate with severity of cognitive decline with aging. We do acknowledge in the Discussion the possible involvement of other regional changes in neuronal populations, or changes in neuronal activity, that might be co-occurring in cognitive decline.

1.10 It is not clear to me whether *g*-ratios were calculated from circular approximations or area measurements, or whether resin shrinkage was corrected. Without this information the quantitative accuracy of the EM data is uncertain. Similarly, classification of "degenerating" profiles appears subjective.
We apologize for the lack of clarity in our methods. We now indicate in the methods that *g*-ratios were calculated by dividing the inner diameter of the axon (assessed by tracing the axon in Fiji, measuring this area, and converting it to the diameter of a circle assuming sphericity, with the equation $2 * \sqrt{\frac{Area}{\pi}}$), by the outer diameter of the myelinated axon (assessed by tracing the outer edge of the myelinated axon and converting it to a diameter, as above).

With regards to the potential for resin shrinkage, the tissue was embedded in Durcupan, which is an epoxy-based resin known for having virtually no shrinkage during the curing process. In contrast to methacrylate-based resins, Durcupan allows uniform hardening due to lack of volatile solvents and non-reactive diluents that can evaporate to cause shrinkage. In addition to this, we would consider it highly unlikely that resin shrinkage would have preferentially occurred in the samples with more severe cognitive decline, given that these samples did not have poorer quality (as indicated by quality control analysis for snRNAseq, **Fig.S2G-I**). Moreover, in our new statistical analyses, we did not find significant correlations between post-mortem interval or brain pH with neuropathological features of severity of cognitive decline (**new Fig.2O; new Fig.S1B**).

We now clarify our criteria for how degenerating profiles were classified in the methods.

1.11 The text does not state whether post-mortem interval, fixation quality, or neuropathological covariates were included in the donor-level correlations (they appear not to be). In their absence, the possibility of confounding remains.

As mentioned above, we have now included new statistical comparisons between clinical and technical variables (age, sex, brain weight, brain pH, post-mortem interval, and co-morbidities) and neuropathological features (**new Fig.S1B**) and see no significant relationships with post-mortem interval or a biochemical marker of tissue quality (brain pH). Tissue quality was also objectively assessed by QC for snRNAseq which showed no donor-level difference in genes/nucleus, UMI/ nucleus, or % mitochondrial genes (**Fig.S2G-I**). We are not able to present data on individual covariates with individual donor information due to GDPR restrictions in place by the Lothian Birth Cohort brain bank, and have therefore presented all data as group level analyses as done previously with this cohort (e.g. *Deary et al, 2025, Mol Psychiatry*); we now indicate this in the methods. We can share the data with the reviewers or other researchers if requested, under a Data Transfer Agreement.

1.12 The donor-level regression slopes and intercepts are not linked to tissue metrics in a supplementary table. Without this, the reproducibility and transparency of the analyses are limited.

We appreciate the suggestion to present the data at the donor level. As indicated above, due to GDPR restrictions with this cohort, we are not allowed to present the data in this way. Rather, we have presented the data as aggregated clinical/donor information and experimental data, done previously for this cohort (e.g. *Deary et al, 2025, Mol Psychiatry*), and have, for all significant results, showed that these do not correlate with any other variables such as post-mortem interval, age, cognition at death, etc.

1.13 Image quantification thresholds were optimised per case. While this may have been necessary due to staining variability, it raises questions about comparability across donors. Without demonstrating robustness to threshold choice or applying normalised fixed thresholds, the reproducibility of these measurements is uncertain.

Image quantification thresholds were optimized per case due to confounding variability in background fluorescence. In the figure below (next page), we show how applying a fixed threshold leads to false positives and missed positives, and now indicate in the methods that we performed this test. We obtained the same significant increase in Olig2+ cells whether quantified with QuPath using variable thresholds, or by blinded manual analysis of 8 technical replicates.

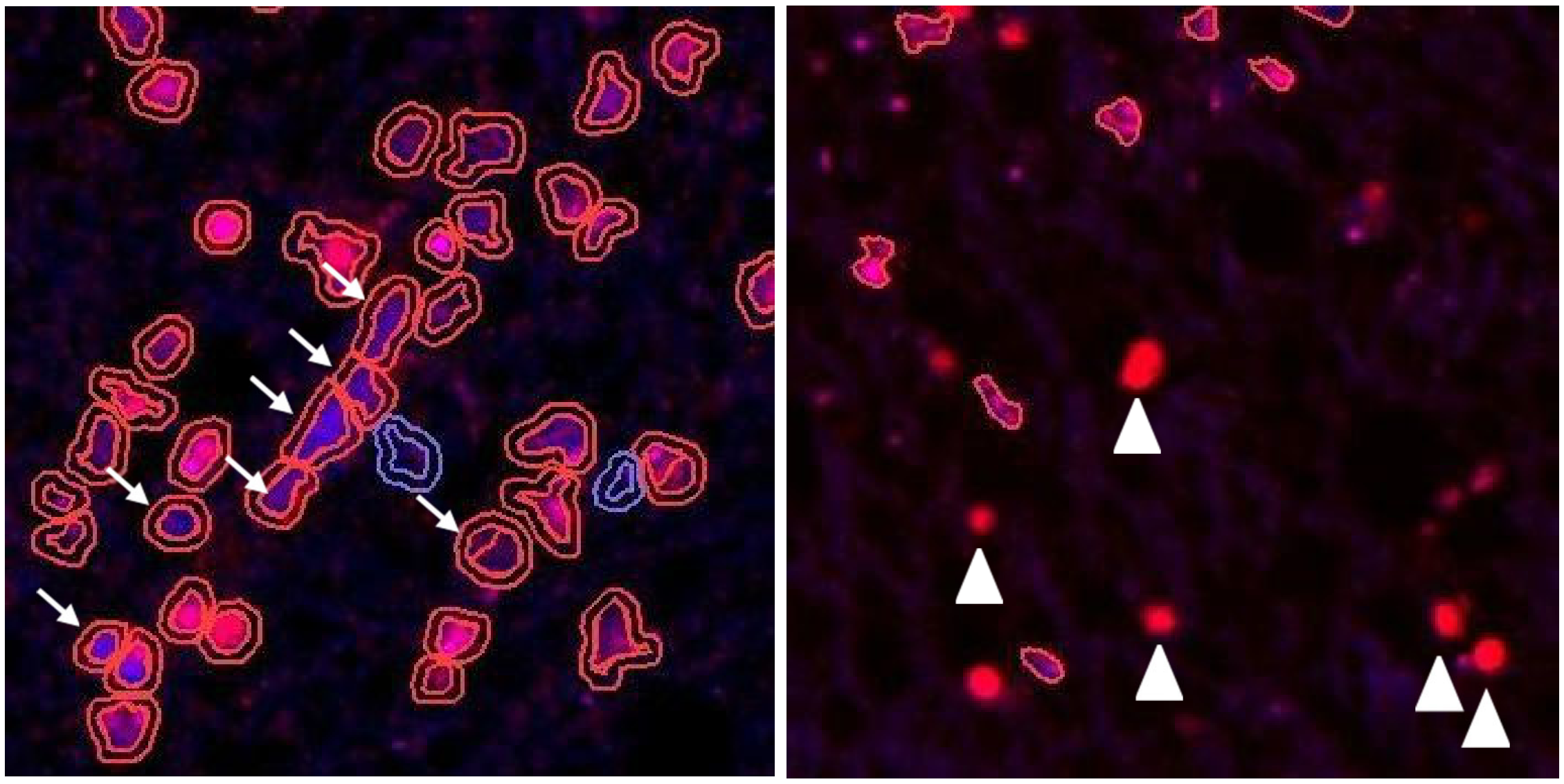

Olig2 (red) and Hoechst (blue) from 2 individuals, quantified using the same fixed threshold. Cells classified as 'detected' are shown outlined in red. Left image shows false positive signal (arrows), while the right image fails to detect Olig2+ cells (triangles).

1.14 "Central white matter" was analysed in humans whereas corpus callosum was analysed in mice; It is not clear to what extent findings from these two regions can be directly generalised across species.

We apologize for not clarifying the terminology for brain region analysed in our human cohort. We had adopted the terminology 'central white matter' based on the original study outlining the capture of brain regions in this cohort (*Henstridge et al, 2015, Acta Neuropathologica Commun*). We now clarify in the methods and results that this refers to (lateral) corpus callosum.

Reviewer #2

The present study leverages a remarkable longitudinal cohort of human subjects tested for cognitive function across their lifespans, together with postmortem brain analyses (EM, snRNA-seq) from a subset of the same individuals. This reveals dysmyelination and increased oligodendrocyte numbers correlated to cognitive decline in aging. These human studies highlighted NRF2 as a mechanism that could account for the poor cognition-associated oligodendroglial and myelin phenotype. Mouse modeling revealed that conditional knockout of NRF2 in oligodendrocytes recapitulated the oligodendroglial and myelin phenotype seen in humans with cognitive decline. This is a beautiful and important study that strongly links dysmyelination in aging with cognitive decline and identifies a molecular mechanism to account for this observation. The data from the human subjects work, from cognitive assessments throughout life to postmortem EM and snRNA seq are exceptionally strong, and the mouse modeling work to validate the NRF2 hypothesis gleaned from human tissue studies is convincing. I have the following suggestions to improve this already excellent manuscript.

2.1 Figure 1J [*now Fig.1H*] shows myelin decompaction (or split artifact) - were *g*-ratio measurements affected by this splitting between wraps? Is the myelin really "thicker", or is it decompacted?

We thank the reviewer for this interesting question. While did see myelin decompaction by electron microscopy, any myelinated axons with extensive myelin decompaction were not included for the myelin thickness analysis due to the potential confounder. Nevertheless, we have now assessed whether small sheath decompactions confounded our *g*-ratio assessments by conducting three new analyses. First, we re-imaged resin samples at higher magnification (20,000X) and found that decompactions were largely localized to small areas rather than being consistent throughout the circumference of the myelin sheath (**Fig.S1C**). When the incidences of these local decompactions were quantified per myelinating axon and plotted as an average per individual, we did see a significant association between the frequency of decompactions and severity of cognitive decline (**new Fig.S1D**), however this did not correlate with *g*-ratio (For all axons: R=-0.36; P=0.22; For large (>1 μm) axons: R=-0.45; P=0.12). Together, this suggests that both decompactions and thicker myelin may be co-occurring in cognitive decline. We are, however, cautious of overinterpreting myelin decompactions on human electron micrographs, as these are the most common artefacts resulting from the standard tissue processing protocol for resin embedding (*Weil et al, 2019, Methods in Molecular Biology; Mobius et al, 2016, Brain Res*). To overcome this and confirm if loss of myelin integrity occurs, we performed SCoRe imaging that allows label-free assessment of compact myelin, which we recently validated for use in human white matter (*Craig et al, 2024, Front Cell Neurosci*). We found a significant decrease in SCoRe signal with worse cognitive decline (**new Fig.1E-G**); this was not confounded by demyelination as there was no significant decrease in total myelinated axon density or proportion (**new Fig.1I-J**). Altogether, these findings suggest that there is both myelin decompaction, and thicker myelin, in individuals with severe cognitive decline.

2.2 Do the oligodendrocyte-specific NFR2-KO mice have cognitive deficits?

As requested, we assessed cognitive performance in oligodendrocyte-specific NRF2 conditional knockout mice. This was done at 12 months of age, when cognition begins to be impaired in wildtype mice. We first performed the Open Field test to screen for major motor or anxiety effects that might confound behavioural readouts, which indicated that females had confounding altered anxiety (**new Fig.S4A,B**), and were thus excluded from further tests. We used the Morris Water Maze to test for cognitive performance over time (diagram in **new Fig.3D**), facilitating comparison to temporal changes in cognition in the human cohort. This platform is highly sensitive to detecting cognitive impairment due to aging (*Wang et al, 2020, Nature Neurosci*) or alterations to corpus callosum oligodendrocytes (*Chen et al, 2021, Neuron; Steadman et al, 2020, Neuron*). While it is a hippocampus-based task, white matter connections through the corpus callosum (e.g. to prefrontal cortex) are required to retrieve spatial information and to support search strategy (*Woolley et al, 2013, PNAS; Sang Jo et al, 2007, J Neurosci; Rossato et al, 2015, Neurobiol Learn Mem*).

Over the course of the testing phase, all mice showed an increase in the percentage of time spent in the target quadrant containing the platform, indicating spatial learning and memory encoding (**new Fig.3E**). As expected,

controls showed a robust improvement in the percentage of time spent in the target quadrant over test trial days, increasing by 57% by day 4 vs day 1. In contrast, conditional knockouts showed an attenuated increase in percentage of time spent in the target quadrant over time, with only a 27% improvement by day 4, which was significantly less than controls (**new Fig.3E-F**). Importantly, blunted improvement was not due to the conditional knockouts reaching the maximum possible percentage time in the target quadrant in prior days, as the absolute percentage of time spent in the target quadrant was lower than controls on days 3 and 4 (**new Fig.3E**).

Relatively worsened learning over time in the conditional knockouts was observed despite showing a surprisingly shorter latency to reach the target quadrant vs controls over all test days (**new Fig.S4E**). As this did not reflect faster swim speed (**new Fig.S4F**), we next investigated search strategy. To quantify search strategy, we applied the approach established by *Quintanilla Sanchez et al, 2023, Biomedicine* which bins search paths in the Morris Water Maze into 8 categories to calculate a 'score' - where more direct spatial strategies correspond to a higher score vs less direct non-spatial strategies (**new Fig.S4G**). This revealed that conditional knockouts engaged a more directed strategy (higher average score/ day) from day 1 (even though they should not be aware of the location of the platform yet, as the platform location moved during the acclimatisation phase), however the conditional knockouts did not improve their strategy over time as much as the controls (**new Fig.S4H,I**). Again, this was not due to the conditional knockouts already having reached the maximum 'directness' in search strategy: i) the maximum score possible is 6 and by the last test day the conditional knockouts had an average score of 4, and ii) both conditional knockouts and controls only ever reached the max. score in ~10% of test trials (**new Fig.S4G**). In summary, these findings indicate that oligodendrocyte NRF2 conditional knockout mice show a blunted capacity to improve cognitive performance over time. This directly links NRF2 loss in oligodendrocytes to cognition for the first time.

While conditional knockouts showed attenuated cognitive improvement over time, we noted a large degree of individual variation in performance across both genotypes. To correlate individual cognitive performance with white matter correlates, as was done in the human, we binned animals based on whether performance over time was better or poorer than average (**new Fig.3G**). There was no significant difference in oligodendrocyte densities between better versus poorer performers (**new Fig.3H**). However, when tissue was assessed by electron microscopy, there was clear variability in ultrastructural features of myelinated axons between better and poorer performers (**new Fig.3I**). Both axon diameter and *g*-ratio were significantly reduced in poorer performers (**new Fig.J,K**), indicating that smaller axons and thicker myelin are correlates of worse cognitive performance in aging mice. In late aging (15-18 months), oligodendrocyte density was unchanged in the conditional knockouts versus controls (**Fig.S4L-N**), however axon diameter and *g*-ratio were significantly reduced (**Fig.3L-O**), suggesting that loss of NRF2 in oligodendrocytes exacerbates the neuropathological features associated with age-related cognitive decline.

Minor suggestions:

2.3 PMID 24727982 should be included in the references for de novo myelin plasticity.

We thank the reviewer for this suggestion and have now referenced this important study in the Discussion.

Reviewer #4:

This is a very interesting and potentially very important study. Bringing together cognitive information on aging humans, neuropathology, and a level of mechanistic insight is a courageous undertaking of the sort that is necessary to reveal a genuinely meaningful understanding of the ageing brain. While the authors are to be congratulated on this undertaking and having pulled off a truly impressive study, there are a number of provisos regarding technical aspects of the study that render emphatic conclusions difficult to make. The analysis of myelinated axons in postmortal human tissue is an heroic endeavour and one that is necessary to address the study's central questions. It is however an undertaking that is fraught with technical difficulty: how confident can one be in distinguishing events that took place during life from those that took place afterwards (tissue deterioration, fixation and processing artefacts etc). These are, of course, not issues that the authors can address with additional experiments, but I think it would be appropriate for the conclusions drawn to be couched inappropriate caveats – even in the results section.

We appreciate the reviewer's caution, and suggestion to take into account the caveats of potential varying tissue quality and processing artefacts in our conclusions. To address this, we provide new correlation matrices which show no statistical relationship between technical confounders or indices of tissue quality (i.e. post-mortem delay, brain pH, brain weight) and oligodendrocyte densities, NRF2+ oligodendrocyte densities, myelin thickness, density of degenerating myelinated axon profiles and myelinated axon density/proportion. We did see a negative association between Braak stage and axon diameter, suggesting that smaller axons may be a shared pathological feature between age-associated cognitive decline and Alzheimer's Disease pathology. We also saw significant associations between average axon mitochondrial size and age at death, absolute cognition at age 70, and extrapolated cognition at death, suggesting that mitochondrial stress may be a readout of absolute cognitive ability but not severity of cognitive decline **(new Fig.2O, new Fig.S1B**). We had also objectively assessed tissue quality in QC checks for snRNAseq, which showed no donor-level difference in features expected to be altered by poor tissue quality (genes/nucleus, UMI/ nucleus, or % mitochondrial genes; **Fig.S2G-I**). Nonetheless, we have now included the following sentence in the results to suggest the potential impact of tissue quality or processing: '*We acknowledge that many clinical parameters and technical artefacts from tissue processing are often confounding factors in human tissue analyses*.'

4.1 Related to this issue is the concept of 'thicker myelin' – a term which could be construed to me to mean that the myelin sheath has increased in diameter. It is more likely that the axon has atrophied and the myelin sheath has stayed the same as it was as it ever was. The authors should perhaps use a more nuanced way describing the changes that they are observing.

We thank the reviewer for putting forward the alternative hypothesis that thicker myelin resulted from primary axonal pathology. We agree that this could have been a possibility, given that the left-shift in axon calibre distributions, increased degenerating profiles, and lower *g*-ratio (increased myelin thickness) are features that could be consistent with a primary axonopathy. To investigate this possibility, we conducted several new analyses of the human white matter.

First, we assessed ultrastructural features known to precede axonopathy: increased mitochondrial size, axonal spheroids, and dark axoplasm - the latter resulting from axon shrinkage-induced condensation of cytoskeletal elements (*Schaffner et al, 2023 Nature Neurosci; Yong et al 2022, Mol Cell Neurosci; Kiryu-Seo et al, 2016, Sci Rep; Barrientos et al, 2011, J Neurosci; Webster, 1962, J Cell Biol*). Importantly, these features are not influenced by inter-sample variations in tissue quality nor stochasticity of sampling along the axon, and represent hallmarks of axon pathology which occur early (mitochondrial size) and late (spheroids) in degeneration. We did not detect any axonal spheroids and dark axoplasm in any sample regardless of cognitive decline severity; this contrasts the abundant axonal spheroids we previously detected in human white matter of the disorder ALSP (*McNamara et al, 2023, Nature*). Axonal mitochondrial size did not significantly differ with severity of cognitive decline (**new Fig.1S,T**). As a control for our capacity to detect increased mitochondrial size, middle-aged adult brain samples were included as a comparison, which confirmed that aging in general causes an increase (**new Fig.1T**) as expected in aging white matter (*Stahon et al, 2016,*

J Neurosci). Second, we used immunofluorescence to assess expression levels of SMI-32, commonly used as a readout of axonal pathology in diseased human brain (e.g. *Schultz et al, 2017, Glia*). As SMI-32 can also pick up healthy neuronal cell bodies (*Campbell and Morrison, 1989, J Comp Neurol*), staining surrounding Hoechst+ nuclei was excluded to specifically quantify axon pathology (please note that all ovoid SMI-32 staining was associated with a nuclei, and therefore did not represent axonal spheroids). Less than 10% of tissue area was positive for SMI-32 across cases, and there was no significant difference based on degree of cognitive decline (**new Fig.1U,V**).

These findings suggest that there is no overt primary axonopathy or differential degree of axonal stress in white matter in association with severity of human cognitive decline in aging. This is consistent with data included in our first submission, where thicker myelin was not observed on the smaller axons (**Fig.1N,O**); if axon shrinkage was occurring in large axons thereby causing the relative increase in small axons, we would expect relatively thicker myelin on the small axons as well. Notably, although thicker myelin can cause axonal stress, axonal stress/pathology does not lead to thicker myelin (*Ineichen et al, 2021, J Neurosci Res*). In addition, we had noted no significant changes in neuronal gene expression in association with severity of cognitive decline (**Fig.S3E**). The neuronal cluster was of equivalent size to that in other human brain sequencing studies (*Jakel et al, 2019, Nature*; *Tran et al, 2021, Neuron*), including where pathology was seen to influence neuronal gene expression (*Tran et al, 2021, Neuron*), indicating that the capture of neuronal nuclei would have been sufficient to detect significant changes associated with stress. Although this does not rule out gene expression changes in neuronal nuclei in other brain regions - which we now acknowledge in the Discussion - together with our neuropathological analyses, it does suggest that readouts of primary neuronal changes in corpus callosum do not appear to associate with severity of cognitive decline.

Furthermore, our findings are in contrast with those in human primary axonopathies and their associated mouse models. For instance, in Giant Axonal Neuropathy (GAN), axons have spheroids, thin myelin sheaths, and undergo robust demyelination (reviewed in *Johnson-Kerner et al, 2014, Muscle Nerve*), none of which are evident in our cohort. Increased mitochondrial size occurs prior to axonal shrinkage and degeneration in the primary axonopathies Hereditary Spastic Paraplegia and Motor Neuron Disease (*Ferreirinha et al, 2004, JCI; Sasaki et al, 2004, Acta Neuropathol*), yet this was not evident in the corpus callosum with severe cognitive decline. Altogether, analysis of aged human brain at the ultrastructural, histopathological, and transcriptional levels do not provide evidence supporting primary axonopathy in the corpus callosum in association with severity of cognitive decline.

4.2 If I have understood correctly, what the authors are describing in the first section of the results is a generalised primary axonal atrophy which is more advanced in larger diameter fibres. Does it not then imply that the key correlation with cognitive decline is loss of axons – in which case would this be a particularly novel observation, especially if the axonal atrophy is as a result of neuronal loss/grey matter atrophy? I suppose to some extent this question is answered in the oligodendrocyte NRF2 deletion studies in mice. Nevertheless, this is an important issue that the authors will need to address.

As summarized in point 4.1, our new data does not support a primary axonopathy in association with severity of cognitive decline in aging. With regards to novelty, we reveal the vulnerability of large myelinated axons in human aging in association with a functional outcome. The literature indicates a preferential loss of small axons with human aging (*Hou et al, 2012, Neurobiol Aging; Marner et al, 2003, J Comp Neurol; Tang et al, 1997, Neurobiol Aging*), and one would have therefore predicted that loss of small axons would associate with severity of cognitive decline. However, our data do not support this postulate (at least in the corpus callosum), and instead show a surprising loss of large axons in association with cognitive decline severity. This suggests that small axon loss is equivalent in aging regardless of cognitive status, and the loss of the large axons in the white matter may have a more robust functional consequence on cognitive decline. Although global MRI-derived measures of brain atrophy have been associated with cognitive decline in LBC1936 and other ageing cohorts, these measures are highly non-specific and cannot identify the cellular processes or axonal properties driving decline. Our study provides a higher biological resolution by showing that oligodendrocyte ageing is linked specifically to vulnerability of large myelinated axons, rather than to a general primary axonopathy or undifferentiated tissue loss. Thus, the novelty lies not in showing that brain tissue loss

relates to cognition, but in identifying a selective white-matter cellular and axonal phenotype associated with cognitive decline severity.

Two additional points of novelty of our study relate to our explanation of this pathology in part through oligodendrocyte dysfunction. We are the first to associate an increase in (dysfunctional) oligodendrocytes with cognitive impairment. Loss of function in the CNS is typically associated with loss of neural cells, e.g. loss of oligodendrocytes in the common white matter disorder multiple sclerosis. Here, we surprisingly show that worse cognition is associated with a counter-intuitive increase in oligodendrocytes. This is unexpected, as increased oligodendrocytes and myelin in adult mice is needed for learning and memory. We show that these responses can be maladaptive, that the oligodendrocyte profile has an influence on myelin and axon integrity, and this is associated with cognitive decline. Indeed, another point of novelty is that we reveal a role for NRF2 in oligodendrocytes in regulating myelin integrity, myelinated axon health, and cognition in aging. Aged oligodendrocyte specific NRF2 conditional knockouts were able to exactly mirror the myelin and axonal pathology of severe human cognitive decline. We now bolster these findings by including new data from cognitive assays in the mice indicating that loss of NRF2 in oligodendrocytes is sufficient to blunt cognitive improvement over time in aging (**new Fig.3D-F**). Moreover, the neuropathological correlates of cognitive decline identified in humans (smaller myelinated axons and thicker myelin) correlated to poorer cognitive performance across aging mice (**new Fig.3G,J,K**), and this pathology is exacerbated with NRF2 loss in oligodendrocytes (**Fig.3L-O**). This is the first indication that NRF2 in oligodendrocytes directly regulates cognitive ability.

4.3 Given these caveats I'm not sure that the authors can really make the claim that oligodendrocytes are a driver of neuropathology. This is perhaps overstating it somewhat and it may be better for them to talk about oligodendrocytes being a key contributor to neuropathology.

We appreciate the reviewer's suggestion to soften our language in interpreting data. Though we believe that our new data indicating that conditional knockout of NRF2 in oligodendrocytes is sufficient to attenuate cognitive improvement over time in aging provides strong evidence for a primary effect, we also acknowledge that other cell types and processes likely contribute to cognitive decline in human aging. We have therefore softened our language throughout the manuscript, e.g. removing the term 'driver' and replacing it with 'contributor', and in the Discussion we now acknowledge the potential contribution of other cell types, processes, and brain regions, to cognitive decline in aging.

Minor issues

4.4 The increased density of oligodendrocytes does not imply de novo generation of new cells. Density readouts will be in part due to absolute numbers of cells but also due to changes in other cell types and parenchymal changes. This should be acknowledged.

We agree that changes in other cell types and parenchymal changes may influence oligodendrocyte densities. We considered the former in the snRNAseq analysis by complementing absolute nuclei counts with proportions of each cell type from the total nuclei detected, which also showed increased oligodendrocytes (**Fig.S2K**). We captured other cell types at frequencies similar to those documented in previous human brain studies, which were previously shown to be sufficient to detect frequency changes in small clusters (e.g. of 22 cells) (*Jakel et al 2019, Nature; Tran et al, 2021, Neuron*), suggesting that we would have been able to detect robust changes in densities of other cell types. Nonetheless, we acknowledge that the lack of change of density/proportions of other cell types requires validation, and we now incorporate the possibility of changes in other cell types into the Discussion.

With respect to parenchymal changes, we now acknowledge in the Discussion that changes associated with the aging brain, such as inflammation and vascular pathology, might also contribute to cognitive decline. We also acknowledge the possibility of maladaptive changes in neuronal activity with aging as a potential contributor. Lastly, we mention the importance of investigating other brain regions in future, which are known to undergo changes in activity in aging in response to cognitive tasks, and may involve other cell types and processes. Nonetheless, we consider it unlikely that there was an increase in oligodendrocytes due to technical artefact. For example, tissue

shrinkage specifically in severely declined cases would have likely resulted in an increase in all cell types, and increased total myelinated axons, which we did not observe (**Fig.2C; new Fig.1I,J**). Even if this were the case and there was attrition of some cells to cancel out an increase, it would be surprising if this was occurring equally across all cell types except oligodendrocytes. Conversely, an increase in oligodendrocytes due to robust tissue atrophy would have likely been associated with a decrease in total myelinated axons, which we also did not observe (**new Fig.1H-J**).

4.5 In the introduction, I wonder whether references 8 to 11 really are the most appropriate ones to support the claim that has been made. Can I suggest the authors look carefully at this and perhaps make some revisions?
We thank the reviewer for this suggestion and have updated the references to be more accurate (*Wang et al, 2020, Nature Neuroscience; Phillips et al, 2019, Am J Primatol*).

4.6 I am not sure that the term central white matter is one that would be widely understood (for my part, I'm not exactly certain what is meant by this term). Perhaps the authors could be more explicit about which anatomical region(s) within the brain they are referring to with this term.
We apologize for not clarifying the terminology for brain region analysed in our human cohort. We had adopted the terminology 'central white matter' based on the original study outlining the capture of brain regions in this cohort (*Henstridge et al, 2015, Acta Neuropathologica Commun*). We now clarify in the methods and results that 'central white matter' refers to (lateral) corpus callosum.

NMED-A144335A

Response to reviewers

We are pleased that our manuscript has been accepted in principle and are grateful for the positive final comments from the reviewers. Regarding Reviewer 1's minor suggestions, we provide a response below. Please note the other reviewers had no suggestions to address.

Reviewer 1:

The authors have addressed my main concerns in a satisfactory manner. In particular, they have added appropriate analyses to test the alternative axon first interpretation, have strengthened the treatment of technical and statistical confounds, and have included new behavioural data in the oligodendrocyte specific Nrf2 conditional knockout mice. The human data necessarily remain associative and do not definitively establish directionality, but the revised manuscript now acknowledges this limitation more appropriately and the language has been sufficiently tempered. I therefore have no further major concerns. I have only two minor suggestions. First, the authors should ensure that any probability distribution analyses are presented descriptively and are not used as the primary basis for statistical inference. Second, the raw nuclei count correlations in Fig. 2C should either be clearly labelled as exploratory or the associated significance values should be removed, given the authors' own acknowledgement that sequencing based abundance estimates are not statistically secure on their own.

We appreciate the reviewer's positive comments on our revisions.

Regarding the probability distribution analyses, no statistical tests have been included on these to avoid statistical inference from these plots.

With regards to the raw nuclei correlations, we have now removed the statistical values from this data (Fig.2C).
